# Implementation research on noncommunicable disease prevention and control interventions in low- and middle-income countries: A systematic review

**DOI:** 10.1371/journal.pmed.1004055

**Published:** 2022-07-25

**Authors:** Celestin Hategeka, Prince Adu, Allissa Desloge, Robert Marten, Ruitai Shao, Maoyi Tian, Ting Wei, Margaret E. Kruk

**Affiliations:** 1 Department of Global Health and Population, Harvard TH Chan School of Public Health, Boston, Massachusetts, United States of America; 2 School of Population and Public Health, University of British Columbia, Vancouver, British Columbia, Canada; 3 School of Public Health, University of Illinois Chicago, Chicago, Illinois, United States of America; 4 Alliance for Health Policy and Systems Research, WHO, Geneva, Switzerland; 5 Department of NCD, WHO, Geneva, Switzerland; 6 The George Institute for Global Health, Faculty of Medicine and Health, University of New South Wales, Sydney, Australia; 7 School of Public Health, Harbin Medical University, Harbin, China; Washington University in St Louis School of Medicine, UNITED STATES

## Abstract

**Background:**

While the evidence for the clinical effectiveness of most noncommunicable disease (NCD) prevention and treatment interventions is well established, care delivery models and means of scaling these up in a variety of resource-constrained health systems are not. The objective of this review was to synthesize evidence on the current state of implementation research on priority NCD prevention and control interventions provided by health systems in low- and middle-income countries (LMICs).

**Methods and findings:**

On January 20, 2021, we searched MEDLINE and EMBASE databases from 1990 through 2020 to identify implementation research studies that focused on the World Health Organization (WHO) priority NCD prevention and control interventions targeting cardiovascular disease, cancer, diabetes, and chronic respiratory disease and provided within health systems in LMICs. Any empirical and peer-reviewed studies that focused on these interventions and reported implementation outcomes were eligible for inclusion. Given the focus on this review and the heterogeneity in aims and methodologies of included studies, risk of bias assessment to understand how effect size may have been compromised by bias is not applicable. We instead commented on the distribution of research designs and discussed about stronger/weaker designs. We synthesized extracted data using descriptive statistics and following the review protocol registered in PROSPERO (CRD42021252969). Of 9,683 potential studies and 7,419 unique records screened for inclusion, 222 eligible studies evaluated 265 priority NCD prevention and control interventions implemented in 62 countries (6% in low-income countries and 90% in middle-income countries). The number of studies published has been increasing over time. Nearly 40% of all the studies were on cervical cancer. With regards to intervention type, screening accounted for 49%, treatment for 39%, while prevention for 12% (with 80% of the latter focusing on prevention of the NCD behavior risk factors). Feasibility (38%) was the most studied implementation outcome followed by adoption (23%); few studies addressed sustainability. The implementation strategies were not specified well enough. Most studies used quantitative methods (86%). The weakest study design, preexperimental, and the strongest study design, experimental, were respectively employed in 25% and 24% of included studies. Approximately 72% of studies reported funding, with international funding being the predominant source. The majority of studies were proof of concept or pilot (88%) and targeted the micro level of health system (79%). Less than 5% of studies report using implementation research framework.

**Conclusions:**

Despite growth in implementation research on NCDs in LMICs, we found major gaps in the science. Future studies should prioritize implementation at scale, target higher levels health systems (meso and macro levels), and test sustainability of NCD programs. They should employ designs with stronger internal validity, be more conceptually driven, and use mixed methods to understand mechanisms. To maximize impact of the research under limited resources, adding implementation science outcomes to effectiveness research and regional collaborations are promising.

## Abstract 2

Celestin Hategeka and colleagues evaluate the current state of implementation research on Non-Communicable Disease prevention and control interventions in low-and middle-income countries (LMICs).

## Author summary

**Why was the study done?:**

- While the evidence for the clinical effectiveness of most noncommunicable disease (NCD) prevention and treatment interventions is well established, care delivery models and means of scaling these up to entire populations in need in heterogeneous and resource-constrained health systems are not.
- Implementation research on NCD program delivery can illuminate what does and does not work in preventing NCDs or achieving NCD control. This can promote faster, more efficient, and more effective scale-up of life-saving and health-preserving health system strategies.
- Evidence needed on the current state of implementation research on World Health Organization (WHO) priority NCD prevention and control interventions to help inform research priority.

**What did the researchers do and find?:**

- We performed a systematic review search in MEDLINE and EMBASE databases from 1990 through 2020 to identify implementation research studies that focused on the WHO priority NCD prevention and control interventions targeting cardiovascular disease, cancer, diabetes, and chronic respiratory disease and provided within health systems in low- and middle-income countries (LMICs).
- We identified 222 eligible studies that evaluated 265 priority NCD prevention and control interventions implemented in 62 countries (6% in low-income countries and 90% in middle-income countries). The number of studies published has been increasing over time. The majority of interventions were focused on either screening (49%) or treatment (39%), while prevention accounted for only 12%.
- Reviewed studies emphasized a few health areas, such as cervical cancer, with many other high-burden conditions little researched. The majority of studies were proof of concept or pilot, quantitative using weaker study designs and targeted the micro level of health system.

**What do these findings mean?:**

- While implementation research on priority NCDs has grown substantially, from under 10 studies per year in early 2000s to 51 studies in 2020, this is still vastly incommensurate with the health burden of NCDs.
- Future studies should prioritize implementation at scale, target higher levels health systems (meso and macro levels), and test sustainability of NCD programs. They should employ designs with stronger internal validity, be more conceptually driven, and use mixed methods to understand mechanisms.
- To maximize impact of the research under limited resources, adding implementation science outcomes to effectiveness research and regional collaborations are promising.

## Introduction

Noncommunicable diseases (NCDs) have become the leading contributors to morbidity and mortality worldwide. They are now responsible for 74% of all global deaths, 77% of which occur in low- and middle-income countries (LMICs) [1,2]. Approximately 85% of NCD deaths among people aged 30 and 69 years occur in LMICs [1]. Cardiovascular diseases are the leading causes of NCD mortality, followed by cancers, respiratory diseases, and diabetes [1]. Together, these 4 NCDs are responsible of over 80% of all premature NCD deaths [1]. Risk factors such as tobacco and alcohol use, physical inactivity, and unhealthy diets result in significantly greater risk of dying from NCDs. Primary, secondary, and tertiary prevention strategies are vital in addressing NCD burden [1]. Sustainable Development Goal (SDG) target 3.4 commits countries to reduce premature mortality from NCDs by a third by 2030 relative to 2015 levels. Recent analysis shows that no LMIC is on track to meet this target for both men and women if they maintain their 2010 to 2016 average rates of decline [3].

NCD prevention and control should not be regarded as a vertical issue separated from other health conditions. The ongoing Coronavirus Disease 2019 (COVID-19) pandemic has put a spotlight on NCDs, as these increased the risk of death for people with COVID infection. Similarly, NCDs increase mortality risk among people with other infectious diseases such as tuberculosis and HIV. It further highlighted the economic and social inequities in who is afflicted with NCDs, in both high-income countries and LMICs. While primary prevention relies on public health, taxation, and other public policy measures, mitigating the health consequences of NCDs also requires strong health systems. Health systems that recognize this challenge and address modifiable risk factors and prioritize the management of NCDs will be better positioned to promote and maintain health. Data from the 2019 World Health Organization (WHO) NCD Country Capacity surveys reveal that only half of 160 countries have national guidelines for NCDs, half have the 6 essential technologies for early detection, diagnosis, and monitoring of NCDs available in primary care facilities of the public health sector, and 20% of countries have 6 (or fewer) of the 11 essential medicines available [4]. Greater prioritization of NCDs within health systems and high-quality care are essential to achieving SDG 3.4 [3]. Beyond this lies an important agenda for tackling the cumulatively large group of rarer NCDs that afflict the world’s poorest people [5].

To support countries in crafting effective NCD strategies, the WHO Assembly endorsed the Global Action Plan for the Prevention and Control of Noncommunicable Diseases 2013–2020 (GAP-NCD) in May 2013 together with a set of evidence-based interventions (best-buys) and policy options in its appendix 3 that was updated in 2016 and provides 84 interventions or policy options [6,7]. Furthermore, WHO has developed a compendium including all available health interventions. The list and compendium aim to assist Member States, as appropriate in specific national contexts, in implementing measures to achieve the 9 global voluntary targets for NCDs and Target 3.4 of the SDGs. Despite recent calls for a new commitment to implementation research for NCDs, a mid-point evaluation of the WHO NCD Global Action Plan 2013–2030 (NCD-GAP) found that “research has been the weakest NCD-GAP objective in terms of implementation and that progress in implementing research linked to the NCD-GAP has been slow and incremental” [8,9].

While the evidence for the clinical effectiveness of most NCD prevention and treatment interventions is well established, care delivery models and means of scaling these up to entire populations in need in heterogeneous and resource-constrained health systems are not. Implementation research on NCD program delivery, including cost effectiveness in various regions, can illuminate what does and does not work in achieving NCD control [8,10–12]. This can promote faster, more efficient, and more effective scale-up of life-saving and health-preserving health system strategies [13,14]. In this systematic review, we aim to synthesize evidence on the current state of implementation research on WHO priority NCD prevention and control interventions provided within health systems in LMICs [6,7,15–17].

## Methods

This systematic review was conducted according to a study protocol registered in PROSPERO (#CRD42021252969) [18].

### Search strategy

Following the Systematic Reviews and Meta-Analyses (PRISMA) checklist [19], we searched for implementation research studies that focused on relevant NCD prevention and control interventions (Table A in S1 Appendix) provided within health systems in LMICs and were published in peer-reviewed journals indexed in MEDLINE and EMBASE databases from 1990 to 2020. The databases were last searched on January 20, 2021. Our search terms included medical subject heading (MeSH) terms and/or key words for 4 key themes (implementation research; NCDs; NCD interventions; LMICs) that were adjusted for each database:

- Implementation research (e.g., implementation research, implementation science, diffusion of innovations, implementation strategies, dissemination science, implementation outcomes).
- NCDs (e.g., cardiovascular disease, cancer, diabetes, chronic respiratory disease).
- Interventions (e.g., smoking cessation, management of hypertension, treatment of acute myocardial infarction, cervical and colorectal cancer screening).
- LMICs as defined by the World Bank in 2019 (Table C in S1 Appendix).

Language restrictions were not applied. Full details of the search strategy are provided in Table B in S1 Appendix.

## Inclusion and exclusion criteria

Table 1 summarizes our review’s specific eligibility criteria. This review includes peer-reviewed, empirical quantitative, qualitative, and mixed method study designs conducted in LMICs that described the implementation of relevant NCD preventive and/or control interventions provided within health systems. Using the updated Appendix 3 of the WHO Global NCD Action Plan 2013–2020, we identified the WHO priority NCD prevention and control interventions [6]. Of these interventions, we selected those that are specifically provided by health systems. This was achieved through discussions and consensus. Table 2 summarizes the intervention categories across eligible NCD risk factors (i.e., tobacco and alcohol use, physical inactivity, and unhealthy diets) and NCDs (i.e., cardiovascular disease, diabetes, cancer, and chronic respiratory disease), and full details are provided in Table A in S1 Appendix. While our search in databases was not restricted to any language, during study screening/review processes, we only retained eligible studies that were in 6 official languages of the United Nations (i.e., Arabic, Chinese, English, French, Russian, and Spanish). We drew on Proctor and colleagues and Glasgow and colleagues to define implementation outcomes eligible for inclusion [20,21]. Nonempirical/primary research studies are not eligible for inclusion (Table 1).

**Table 1:** Inclusion and exclusion criteria.

	Inclusion criteria	Exclusion criteria
Population	Human beings with or without NCDs. Human beings with or without NCD risk factors.	Subjects are not human beings.
Intervention	NCD prevention and/or control interventions that are provided within health systems (see Table A in S1 Appendix).	Interventions that are not specified in the inclusion criteria.
Outcome	Implementation outcomes as defined by Proctor and colleagues and Glasgow and colleagues [20,21] • Acceptability • Adoption • Appropriateness • Feasibility • Fidelity • Penetration • Sustainability • Implementation costs • Reach • Implementation • Maintenance	Outcomes other than those specified in the inclusion criteria.
Study design	Quantitative, qualitative, or mixed method. Quantitative study designs included experimental and observational. • Experimental designs: ○ Randomized controlled trial, ○ Cluster randomized trial, ○ Randomized step wedge, • Observational designs: ○ Quasi-experimental designs: ▪ Single interrupted time series, ▪ Controlled interrupted time series, ▪ Pre-post with comparison group, ▪ Regression discontinuity, ▪ Nonrandomized stepped wedge ○ Preexperimental designs (no control group or no repeated measures): ▪ Pre-post ▪ Post-only design ○ Other observational designs include: ▪ Cohort studies ▪ Cross-sectional studies ▪ Case-control studies	Nonempirical/primary research including: • Review • Meta-analysis • Editorial • Commentary • Letter to editor • Opinion paper • Newspaper • Protocols • Case report • Epidemiological/descriptive studies (e.g., nonintervention association studies including knowledge, attitude, discrete choice experiment, awareness, willingness, and perception (including perceived barriers) studies) and not in the context of implementation of NCD interventions. • Instrument/screening or diagnostic test validation studies • Call to action • Sharing experience/lessons learned on the field if not resulting from research • (Descriptive) cost-effectiveness studies based on modeling (and not in the context of implementation of NCD interventions)
Geographic Scope	LMICs (see Table C in S1 Appendix)	Areas other than LMICs
Time frame	1990–2020	Studies published before 1990

LMIC, low- and middle-income country; NCD, noncommunicable disease.

**Table 2:** Summary of eligible NCD preventive and control interventions.

Conditions	Intervention categories
**NCD risk factors**	
Tobacco use	Individual smoking cessation
	Mass media campaign smoking cessation
Harmful use of alcohol	Alcohol reduction counseling for at risk individuals
	Treatment for alcohol use disorder
Unhealthy diet	Mass media or other behavior change program to reduce salt intake
	Nutrition education in institutions
	Salt reduction public institutions
	Interventions to promote exclusive breastfeeding
Physical inactivity	Community environmental program increase physical activity
	Mass media campaign promote physical activity
	Physical activity counseling
**NCDs**	
Cardiovascular disease	Treatment of hypertension
	Rehabilitation of post-acute CVD event (myocardial infarction, stroke)
	Treatment of high-risk CVD event
	Treatment of acute ischemic stroke
	Treatment of acute myocardial infarction
	Treatment of heart failure
	Antibiotic treatment of streptococcal pharyngitis (rheumatic fever prevention)
	Treatment for secondary prevention of stroke (e.g., anticoagulation for atrial fibrillation, aspirin)
Diabetes	Glycemic control among people with diabetes
	Screening to prevent complications among people with diabetes
	Treatment of diabetes
	Preconception care for women with diabetes
	Influenza vaccination for people with diabetes
Cancer	Breast cancer screening
	Cervical cancer screening
	HPV vaccination for teen girls
	Colorectal cancer screening
	Treatment of breast and colorectal cancer
	Hepatitis B immunization for liver cancer prevention
	Screening for oral cancer in high-risk groups
Chronic respiratory disease	Treatment of asthma and COPD
	Influenza vaccination for patients with COPD

COPD, chronic obstructive pulmonary disease; CVD, cardiovascular disease; HPV, human papilloma virus; NCD, noncommunicable disease.

### Data extraction and analysis

The titles and abstracts of unique results from the databases were reviewed independently by 2 researchers for potential inclusion using COVIDENCE review software [22]. The full texts of studies retained at the title and abstract screening stage were retrieved and independently assessed for inclusion. Any discrepancies were resolved through discussion and consensus. Data extraction on each included study was conducted by a single researcher using a data extraction tool, developed and piloted a priori (Table D in S1 Appendix). Data elements included study characteristics (e.g., publication year, country of implementation, study funding), NCD conditions (risk factors and disease), intervention details (e.g., type of intervention, level of health system), methods (e.g., research approach, study design), implementation outcomes (e.g., fidelity, feasibility), and equity lens (e.g., disaggregated by key SES stratifiers, targeted vulnerable population). We also extracted data on implementation strategies including actor (i.e., who delivered the intervention), action target, and recipients; details of other implementation strategies were not sufficiently described to permit extraction [23]. The recipients of the action/strategy were further aggregated by demographic subgroup (e.g., people eligible for cancer screening including cervical and colorectal), disease risk subgroup (e.g., patients with myocardial infarction, patients with diabetes or hypertension, people who smoke), general population, healthcare workers (e.g., physicians, nurses, pharmacists, and midwives), and community health workers. We synthesize extracted data using descriptive statistics and following the review protocol registered in PROSPERO. Specifically, we provide an overview of NCD priority intervention implementation study characteristics across NCD conditions to shed light on the current state of implementation research of priority NCD prevention and control interventions in LMICs. Given this review does not focus on effect size of NCD interventions, we did not perform a meta-analysis.

### Risk of bias assessment

This review focuses on implementation of multiple interventions across various NCDs, rather than effectiveness of any single set of interventions. Further, studies with heterogenous aims and methodologies (including qualitative methodology) were included. Therefore, risk of bias assessment to understand how effect size may have been compromised by bias is not applicable in this review. We instead commented on the distribution of research designs and discussed about stronger/weaker designs.

## Results

**Fig 1:**
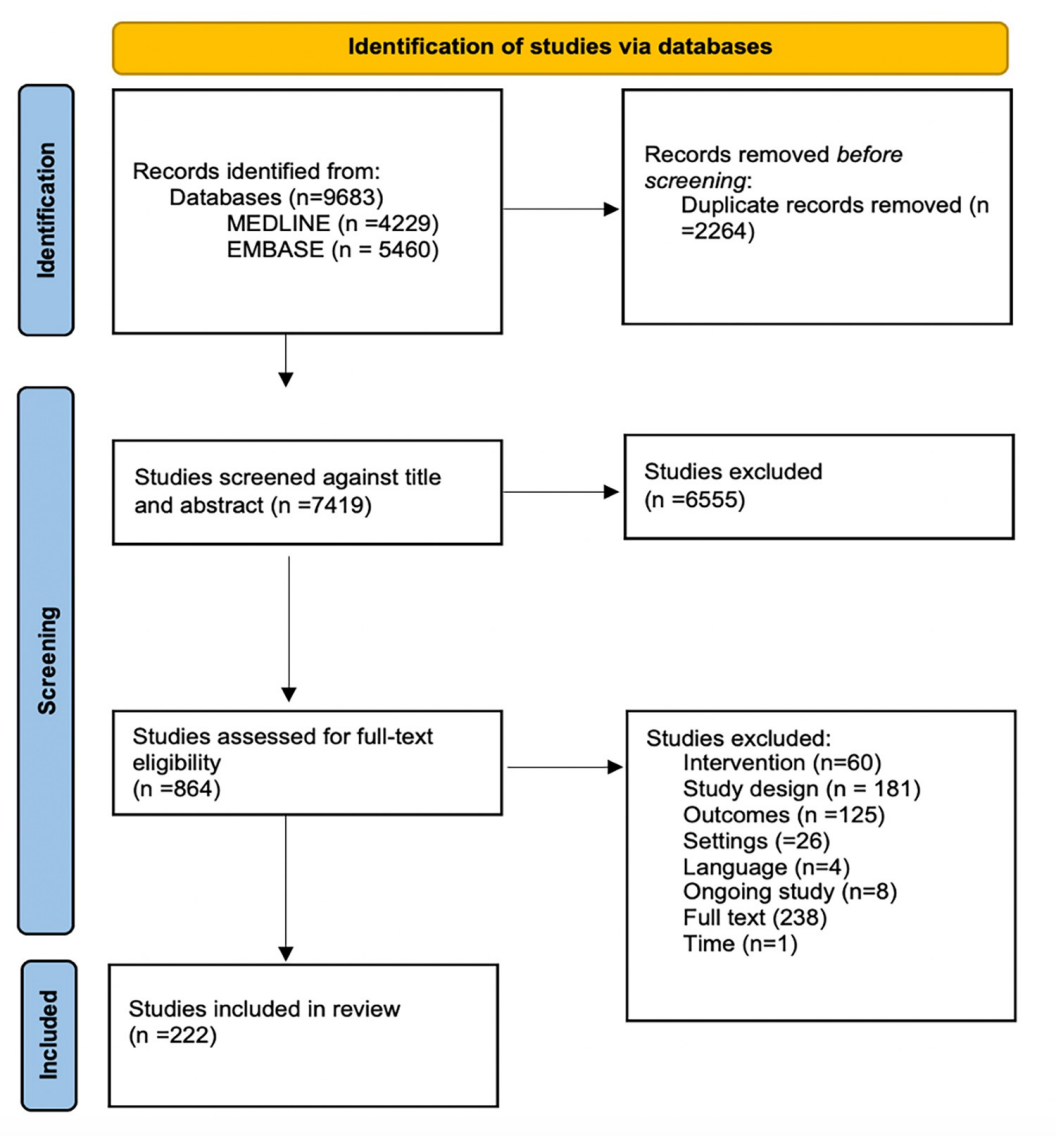
PRISMA flow chart. Intervention refers to studies excluded because they studied the implementation of interventions that did not meet the eligible criteria. Study design refers to studies excluded because they used study designs that did not meet eligibility criteria (e.g., nonempirical studies including reviews and commentaries). Outcomes refer to studies excluded based on not having focused on relevant implementation outcomes. Settings refer to studies excluded because they were not conducted in LMICs. Full text means that studies were excluded because full text was not available. Time refers to studies that were excluded because they were published before/conducted before 1990.

Our search strategy implemented in MEDLINE and EMBASE identified 9,683 publications, of which 7,419 unique records were screened for inclusion. Abstract and full-text screening identified 222 studies that met our inclusion criteria (Tables 1 and 2) [24–245]. A summary of this process is presented in the PRISMA flow diagram in Fig 1.

**Fig 2:**
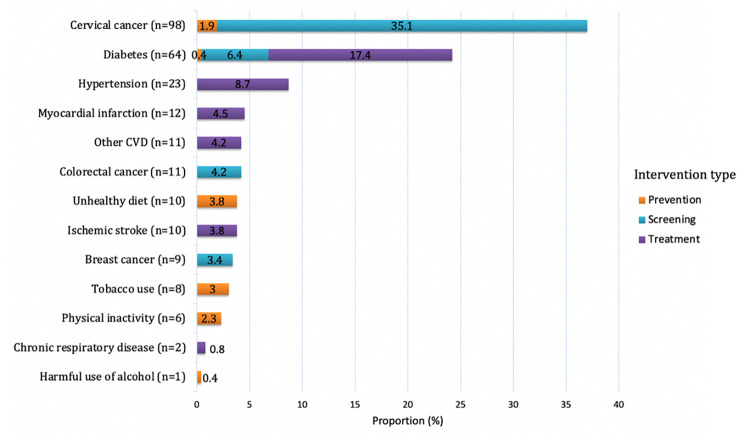
Distribution of priority NCD prevention and control interventions by type of NCD and their risk factors (*N* = 265).

**Fig 3:**
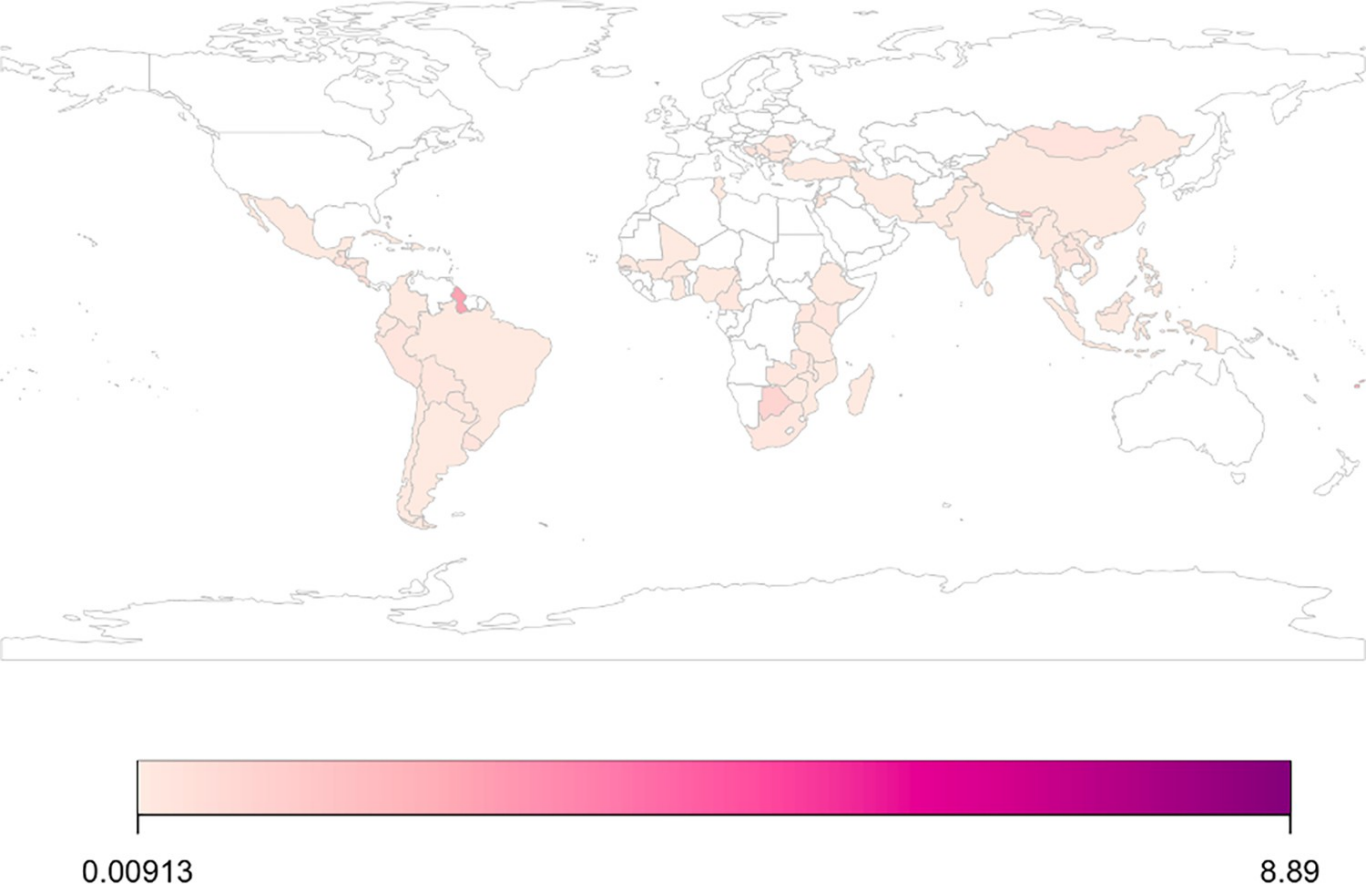
Distribution of studies per 1 million population by country of implementation. We used country population size in 2020 (https://data.worldbank.org/indicator/SP.POP.TOTL) to standardized estimates expressed as number of studies per 1 million population. We used “rworldmap” package (https://cran.r-project.org/web/packages/rworldmap/rworldmap.pdf) available in R software to present these standardized estimates across countries where interventions were implemented. Country borders in this package are derived from Natural Earth data. Table E in S1 Appendix shows number of included studies per country.

**Fig 4:**
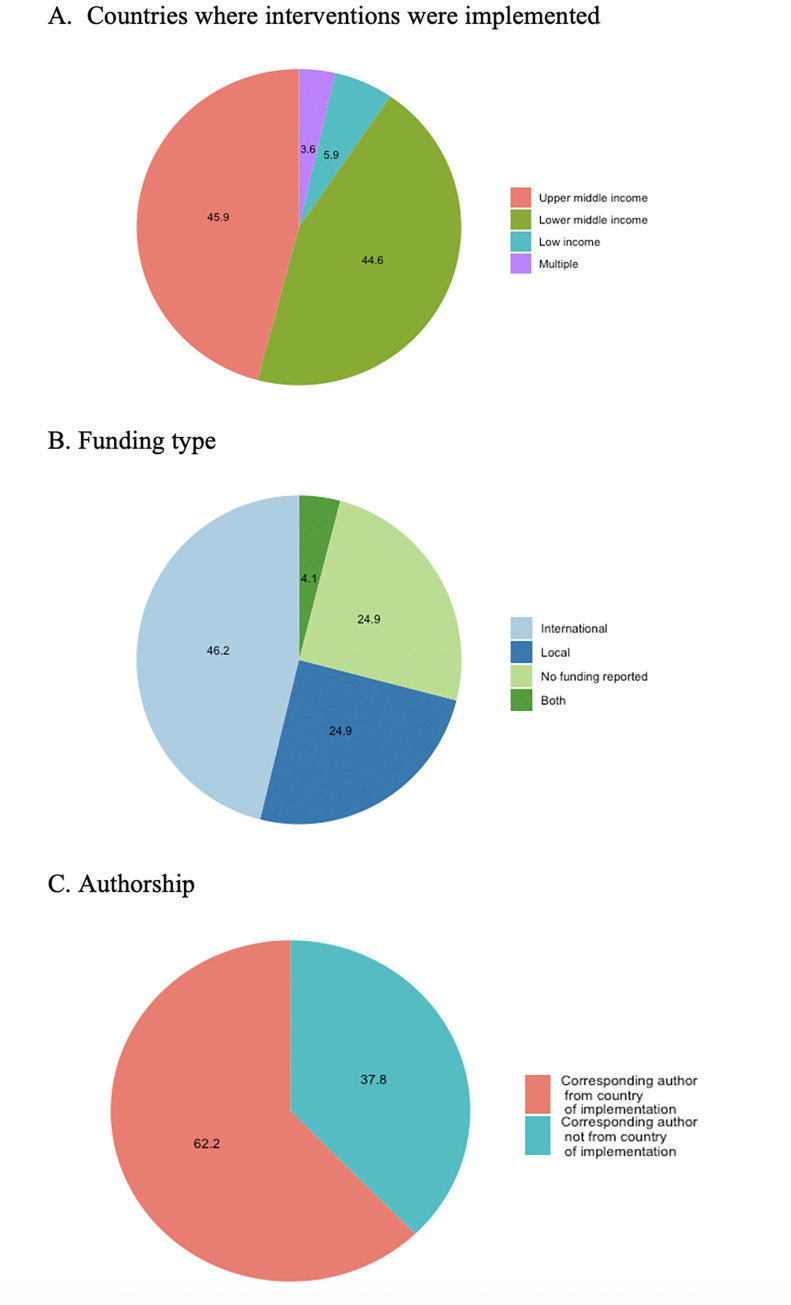
Distribution of study countries, funding, and authorship (*N* = 222).

**Fig 5:**
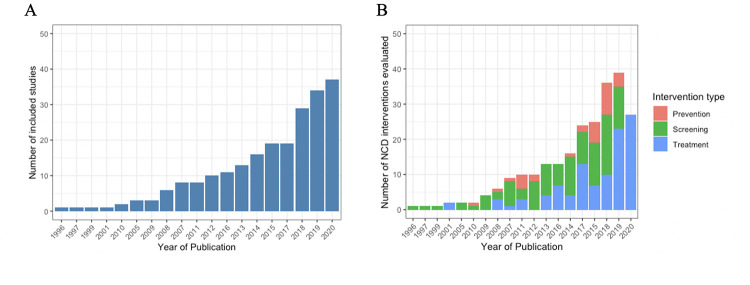
**Growth of research over time (A) and distributions of NCD interventions by type (B).** Fig 5A shows number of studies published each year (*N* = 222 studies); Fig 5B shows distributions by type of interventions (*N* = 265 NCD interventions evaluated in studied included in the review).

**Fig 6:**
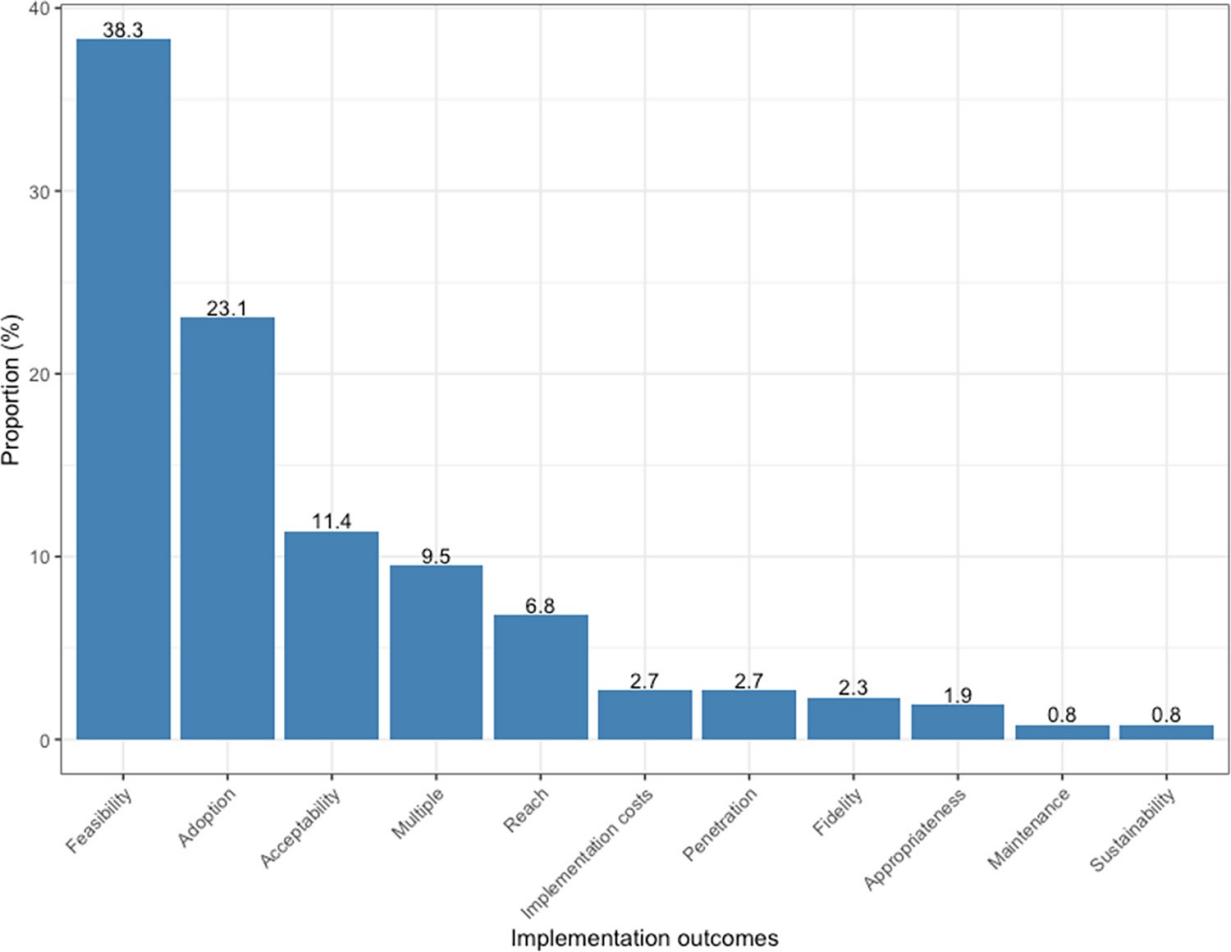
Distribution of implementation outcomes.

The 222 studies included in this review evaluated 265 priority NCD prevention and control interventions implemented in 62 countries, of which 6% were in low-income countries (LICs), 45% in LMICs, and 46% in upper middle-income countries (UMICs) (Table 3 and Figs 2, 3, and 4A and Table E in S1 Appendix). The NCD conditions targeted varied by income groups of countries (Fig A in S1 Appendix). Eight of the included studies were multicountry studies. The number of studies published has been increasing over time (Fig 5A). Overall, the majority of interventions were focused on either screening (49%) or treatment (39%), while prevention accounted for only 12%, with nearly 80% of these tackling prevention of the shared NCD behavioral risk factors—tobacco use, unhealthy diet, physical inactivity, and harmful use of alcohol. The NCD interventions varied by conditions and type (prevention, screening, and treatment) (Figs 2, B, and C in S1 Appendix). Notably, over one-third of the interventions studied (37%) were for cervical cancer (Fig 2), which accounts for 0.35% of DALYs lost and 0.5% of deaths globally, with similar figures for LMICs (https://vizhub.healthdata.org/gbd-compare/). Diabetes was the focus of nearly one-quarter of the research with hypertension the topic of another 9% (Fig 2). Each of the other recommended interventions represented 5% or less of the implementation research output. Chronic respiratory disease was understudied relative to its prevalence: less than 1% of the studies examined chronic respiratory disease treatment and only 3% smoking cessation programs. The intervention focus appears to vary by income groups of countries (Fig D in S1 Appendix). Feasibility was the most studied implementation outcome followed by adoption (Fig 6). Most of the actors were researchers, which accounted for 58%; whereas government/ministry of health, providers, and NGOs accounted for 18%, 10%, and 6%, respectively. The majority of intervention targeted improvement in health outcomes (45%) followed by change in behavior (34%).

**Table 3:** Overview of study characteristics.

NCDs and risk factors	Intervention categories	N	Distribution of priority NCD interventions (*N* = 265)									
			Country’s income classification, N	Methods approach, N	Major study design, N	Health system level*, N	Level of scale-up, N	Implementation outcomes, N	Considered equity†, N	Implementation strategies		
										Actor, N	Action target, N	Recipients, N
Tobacco use	Individual smoking cessation	6	LMICs = 5 UMICs = 1	Quantitative = 5 Mixed = 1	Experimental = 2 Multiple = 1 Preexperimental = 2 Other Observational = 1	Micro = 4 Meso = 2	Pilot† = 5 Scale-up = 1	Adoption = 1 Appropriateness = 1 Feasibility = 3 Multiple = 1	4	Researchers = 4 Providers = 2	Behavior = 6	Disease risk subgroup = 6
	Mass media campaign smoking cessation	2	UMICs = 2	Quantitative = 2	Experimental = 1 Observational = 1	Macro = 2	Scale-up = 2	Adoption = 1 Penetration = 1	1	Researchers = 1 MOH = 1	Behavior = 2	Disease risk subgroup = 2
Harmful use of alcohol	Alcohol reduction	1	LMICs = 1	Quantitative = 1	Experimental = 1	Micro = 1	Pilot = 1	Multiple = 1	0	Researchers = 1	Behavior = 1	Disease risk subgroup = 1
Unhealthy diet	Mass media or other behavior change program to reduce salt intake	3	LMICs = 2 UMICs = 1	Quantitative = 3	Experimental = 2 Other observational = 1	Micro = 1 Meso = 1 Macro = 1	Pilot = 1 Scale-up = 2	Adoption = 1 Penetration = 1 Multiple = 1	2	Researchers = 1 MOH = 2	Behavior = 3	General population = 2 Disease risk subgroup = 1
	Nutrition education in institutions	5	LMICs = 1 UMICs = 3 Multiple = 1	Quantitative = 4 Mixed method = 1	Quasi-experimental designs = 3 Preexperimental = 1 Other observational = 1	Micro = 1 Meso = 3 Macro = 1	Pilot = 2 Scale-up = 3	Acceptability = 1 Adoption = 2 Feasibility = 1 Penetration = 1	3	Researchers = 4 MOH = 1	Behavior = 3 Behavior, health outcomes = 2	Demographic subgroup = 2 Disease risk subgroup = 3
	Salt reduction public institutions	2	UMICs = 2	Quantitative = 2	Other observational = 2	Macro = 2	Pilot = 1 Scale-up = 1	Adoption = 1 Penetration = 1	1	Researchers = 1 MOH = 1	Behavior = 2	Demographic subgroup = 2
Physical inactivity	Community environmental program increase physical activity	4	LMICs = 3 UMICs = 1	Quantitative = 4	Experimental = 2 Preexperimental = 1 Other observational = 1	Micro = 1 Meso = 1 Macro = 2	Pilot = 1 Scale-up = 3	Feasibility = 1 Penetration = 1 Multiple = 2	2	Researchers = 3 MOH = 1	Behavior = 2 Behavior and knowledge = 2	Demographic subgroup = 3 Disease risk subgroup = 1
	Mass media campaign promote physical activity	2	UMCIs = 2	Quantitative = 2	Experimental = 1 Other observational = 1	Macro = 2	Scale-up = 2	Adoption = 1 Penetration = 1	1	Researchers = 1 MOH = 1	Behavior = 1 Behavior and knowledge = 1	Demographic subgroup = 1 Disease risk subgroup = 1
CVD	Rehabilitation post-acute CVD event	1	UMICs = 1	Quantitative = 1	Experimental = 1	Micro = 1	Pilot = 1	Feasibility = 1	0	Researchers = 1	Health outcomes = 1	Disease risk subgroup = 1
	Treatment of high-risk CVD event	5	LMICs = 2 UMICs = 3	Quantitative = 5	Experimental = 2 Quasi-experimental designs = 2 Other observational = 1	Micro = 5	Pilot = 5	Acceptability = 1 Adoption = 2 Feasibility = 1 Maintenance = 1	3	Researchers = 4 Providers = 1	Behavior = 3 Health outcomes = 1	Demographic subgroup = 1 Disease risk subgroup = 3 HCWs = 1
	Treatment of acute ischemic stroke	10	LMICs = 5 UMICs = 5	Quantitative = 10	Experimental = 2 Preexperimental = 7 Other observational = 1	Micro = 6 Meso = 4	Pilot = 8 Scale-up = 2	Adoption = 4 Feasibility = 4 Fidelity = 1	1	Researchers = 5 MOH = 4 Providers = 1	Health outcomes = 10	Disease risk subgroup = 10
	Treatment of acute myocardial infarction	12	LMICs = 2 UMICs = 10	Quantitative = 11 Qualitative = 1	Experimental = 3 Quasi-experimental designs = 2 Preexperimental = 4 Other observational = 3	Micro = 6 Macro = 6	Pilot = 10 Scale-up = 1	Adoption = 5 Feasibility = 5 Fidelity = 1 Penetration = 1	3	Researchers = 4 MOH = 4 Providers = 4	Health outcomes = 11 Behavior = 1	Disease risk subgroup = 11 HCWs = 1
	Treatment of heart failure	5	LMICs = 2 UMICs = 3	Quantitative = 5	Experimental = 2 Quasi-experimental designs = 1 Preexperimental = 2	Micro = 5	Pilot = 5	Adoption = 2 Feasibility = 3	1	Researchers = 3 Providers = 2	Health outcomes = 5	Disease risk subgroup = 5
	Treatment of hypertension	23	LMICs = 10 Multiple = 2 UMICs = 11	Quantitative = 16 Qualitative = 1 Mixed method = 6	Experimental = 7 Quasi-experimental designs = 4 Preexperimental = 2 Other observational = 5 Multiple = 5	Micro = 20 Meso = 2 Macro = 1	Pilot = 22 Scale-up = 1	Adoption = 1 Feasibility = 18 Fidelity = 3 Multiple = 1	10	Researchers = 10 MOH = 5 NGO = 1 Providers = 6 NC = 1	Behavior = 10 Health outcomes = 8 Behavior and health outcomes = 5	Demographic subgroup = 1 Disease risk subgroup = 22
Diabetes	Glycemic control among people with diabetes	7	LMICs = 3 UMICs = 2 Multiple = 2	Quantitative = 5 Mixed method = 2	Experimental = 2 Quasi-experimental designs = 4 Multiple = 1	Micro = 4 Meso = 2 Macro = 1	Pilot = 6 Scale-up = 1	Adoption = 1 Appropriateness = 2 Feasibility = 2 Multiple = 2	4	Researchers = 5 MOH = 1 NGO = 1	Behavior = 4 Health outcomes = 2 Behavior and health outcomes = 1	Demographic subgroup = 1 Disease risk subgroup = 6
	Screening to prevent complications among people with diabetes	17	LMICs = 10 UMICs = 7	Quantitative = 16 Mixed method = 1	Experimental = 1 Quasi-experimental designs = 1 Preexperimental = 6 Other observational = 8 Multiple = 1	Micro = 16 Meso = 1	Pilot = 1	Acceptability = 1 Adoption = 2 Feasibility = 10 Multiple = 1 Reach = 3	6	Researchers = 8 MOH = 1 NGO = 3 Providers = 4 NC = 1	Behavior = 5 Health outcomes = 12	Disease risk subgroup = 16 CHWs = 1
	Diabetes management	39	LMICs = 22 UMICs = 14 Multiple = 3	Quantitative = 33 Qualitative = 1 Mixed method = 5	Experimental = 8 Quasi-experimental designs = 6 Preexperimental = 9 Other observational = 12 Multiple = 4	Micro = 34 Meso = 3 Macro = 2	Pilot = 38 Scale-up = 1	Acceptability Adoption = 1 Appropriateness = 2 Feasibility = 21 Fidelity = 1 Reach = 3 Multiple = 4	16	Researchers = 22 MOH = 6 NGO = 4 Providers = 6 NC = 1	Behavior = 11 Behavior and knowledge = 3 Health outcomes = 20 Behavior and health outcomes = 4 Knowledge and health outcomes = 1	Demographic subgroup = 3 Disease risk subgroup = 35 CHWs = 1
	Influenza vaccination for people with diabetes	1	UMICs = 1	Quantitative = 1	Other observational = 1	Micro = 1	Pilot = 1	Adoption = 1	0	Researchers = 1	Health outcomes = 1	Disease risk subgroup = 1
Cancer	Breast cancer screening	9	LMICs = 5 UMICs = 4	Quantitative = 9	Experimental = 1 Quasi-experimental designs = 2 Preexperimental = 2 Other observational = 4	Micro = 6 Macro = 9	Pilot = 7 Scale-up = 1	Acceptability = 1 Adoption = 1 Feasibility = 4 Implementation cost = 1 Reach = 1 Multiple = 1	7	Researchers = 4 MOH = 4 NC/NA = 1	Behavior = 4 Health outcomes = 5	Demographic subgroup = 9
	Cervical cancer screening	93	LICs = 13 LMICs = 42 UMICs = 34 Multiple = 4	Quantitative = 78 Qualitative = 8 Mixed method = 7	Experimental = 16 Quasi-experimental designs = 4 Preexperimental = 20 Other observational = 47 Multiple = 6	Micro = 78 Meso = 10 Macro = 5	Pilot = 78 Scale-up = 12	Acceptability = 22 Adoption = 23 Feasibility = 22 Implementation cost = 4 Maintenance = 1 Reach = 10 Sustainability = 2 Multiple = 9	40	Researchers = 56 MOH = 13 NGO = 10 Providers = 2 NC = 6 NA = 6	Behavior = 30 Health outcomes = 41 Knowledge = 5 Knowledge and behavior = 4 Knowledge, behavior, health outcome = 2 Knowledge and health outcomes = 3 NC/NA = 8	Demographic subgroup = 80 Disease risk subgroup = 2 HCWs = 3 CHWs = 2
	HPV vaccination for teen girls	5	LMICs = 2 UMICs = 2 Multiple = 1	Quantitative = 4 Qualitative = 1	Preexperimental = 2 Other observational = 3	Micro = 3 Macro = 2	Pilot = 3 Scale-up = 2	Adoption = 3 Feasibility = 1 Multiple = 1	1	Researchers = 3 MOH = 1 NGO = 1	Behavior = 2 Health outcomes = 3	Demographic subgroup = 5
	Colorectal cancer screening	11	LMICs = 1 UMICs = 10	Quantitative = 10 Mixed method = 1	Experimental = 2 Quasi-experimental designs = 1 Preexperimental = 1 Other observational = 6 Multiple = 1	Micro = 7 Meso = 4	Pilot = 9 Scale-up = 2	Acceptability = 1 Adoption = 4 Feasibility = 3 Implementation cost = 1 Reach = 1 Multiple = 1	4	Researchers = 7 MOH = 4	Behavior = 5 Health outcomes = 5 Knowledge and behavior = 1	Demographic subgroup = 10 Disease risk subgroup = 1
Chronic respiratory disease	Treatment of asthma	2	LMICs = 2	Mixed method = 2	Multiple = 2	Micro = 1 Macro = 1	Pilot = 1 Scale-up = 1	Acceptability = 1	1	Researchers = 1 MOH = 1	Health outcomes = 2	Disease risk subgroup = 2

*Micro level refers to the point where the care providers interact with the patient; micro-level interventions aim to directly influence the performance of the staff or the operations of a facility [11,264]. Meso level refers to the level responsible for service areas/clinical programs providing care for a similar group of patients, typically part of a larger organization (e.g., subnational intervention targeting improvement of a network of facilities and communities) [11,264]. Macro level is the highest (strategic) level of the system, an umbrella including all intersecting areas, departments, providers, and staff (e.g., boards, healthcare network, integrated health system that includes several organizations); macro-level interventions are best able to directly tackle the social, political, economic, and organizational structures that shape a health system [11,264].

†Equity lens used if studies disaggregated by SES stratifiers (e.g., age, sex, education, income, and rural vs. urban) and/or targeted vulnerable population.

CHW, community health workers include ASHAs in India; CVD, cardiovascular disease; HCW, healthcare worker; HPV, human papilloma virus; LIC, low-income country; LMIC, lower middle-income country; MOH, Ministry of Health/Government; N, number of NCD interventions; NC/NA, not clear/not applicable; NCDs, noncommunicable disease; NGO, nongovernmental organization; UMIC, upper middle-income country.

**Fig 7:**
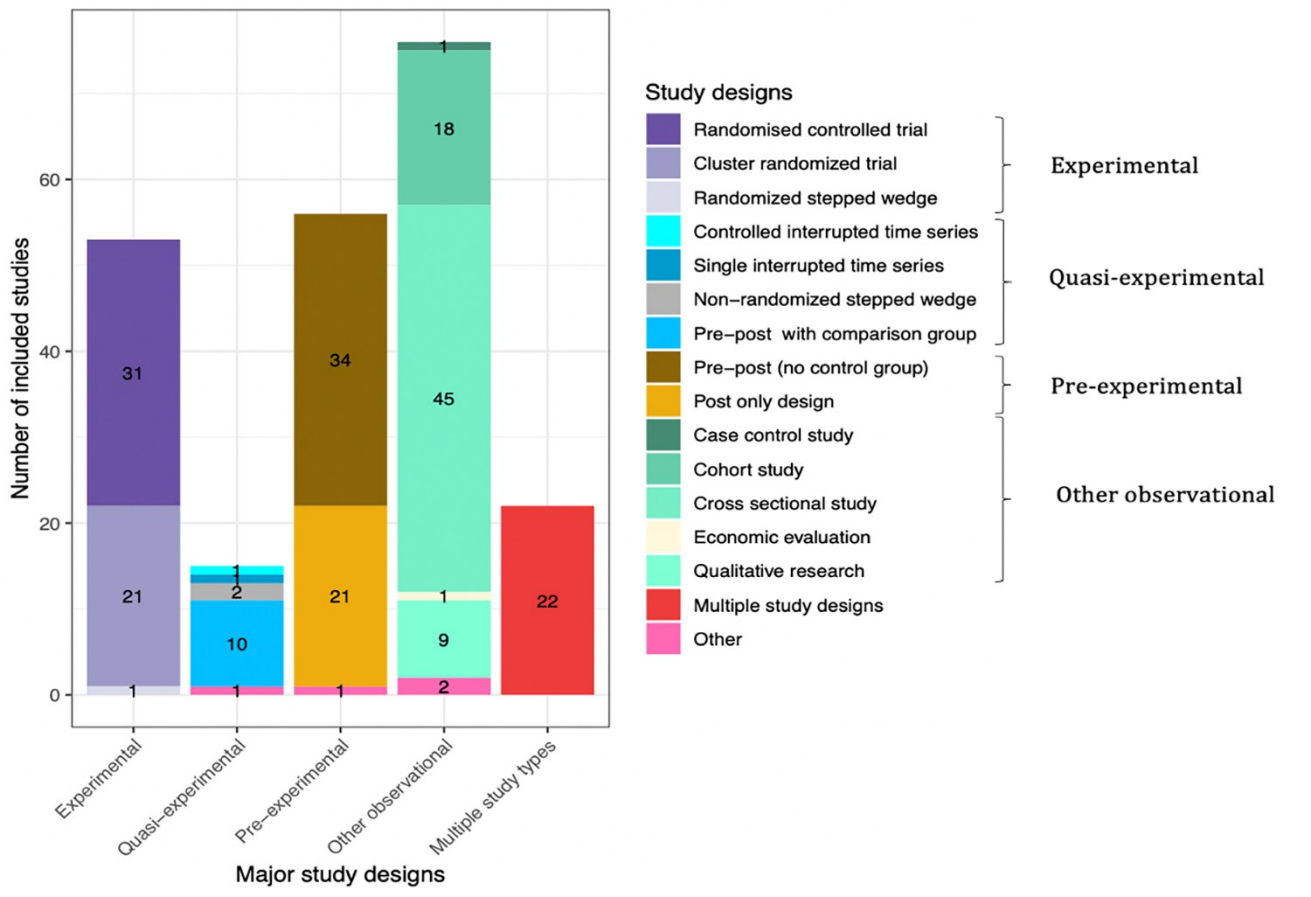
Study designs.

Most studies used quantitative methods, which accounted for 86%, whereas mixed methods and qualitative methods accounted for 9% and 5%, respectively (Table 2). The majority of studies used observational designs, with cross-sectional designs used in 45 studies. Among evaluations, preexperimental studies (such as pre-post without a comparison group or post-only) was the most frequently employed (*n* = 56 or 25% of all studies); experimental designs were used in a quarter of studies (*n* = 53 or 24% of all studies); quasi-experimental evaluation designs (such as pre-post comparison group or time series) were used in 15 papers (7% of all studies) (Fig 7). Study designs also appear to vary by NCD conditions targeted (Fig E in S1 Appendix). The sample size among included studies varied, ranging from 11 to 350,581, with median of 658. Most studies were standalone implementation studies (85%), with some variations by NCD conditions (Fig F in S1 Appendix). Hybrid implementation and effectiveness studies accounted only for 15%. Less than 5% of studies reported they were guided by widely known implementation science framework. Majority of studies were proof of concept or pilot versus scale-up studies (88% versus 12%), with variations by NCD conditions (Fig G in S1 Appendix). The level of health system targeted most often was micro level, accounting for 79% of studies, with variations by NCD conditions. The meso and macro levels of health systems were targeted by 14% and 7% of studies, respectively (Fig H in S1 Appendix). Approximately 42% of studies employed an equity lens—i.e., studies disaggregated by SES stratifiers (e.g., age, sex, education, income, and rural versus urban) and/or targeted vulnerable population.

A majority of studies (72%) reported funding, with international funding being the predominant source (Fig 4B). There seems to be some variations by NCD conditions (Figs I–K in S1 Appendix). For example, while 78% of studies focused on cervical cancer reported funding, of which 77% were from international sources, those focused on colorectal cancer and treatment of acute myocardial infarction received most of their funding from the countries where implementation research was conducted (Fig K in S1 Appendix). Majority of reported funding was provided by government/universities (43.6%), 35% reported multiple funders, 16% were foundations/NGOs, and 6% were private funders (e.g., pharmaceutical companies, professional associations) (Fig L in S1 Appendix). Approximately 62% of corresponding authors were from the country of implementation (Fig 4C); however, this varied by funding sources, with studies funded by international funders having the highest number of international corresponding authors.

## Discussion

We conducted a systematic review of implementation research studies on NCD prevention and control strategies in LMICs published between 1990 and 2020. We focused our analysis on WHO-recommended NCD interventions carried out by the health system rather than through policy, legislation, or public health approaches [6,7]. These studies therefore represent the state of the implementation science in prevention and control of NCDs by health systems in the countries bearing the bulk of disease burden from noncommunicable conditions.

Of the 222 implementation science studies included in this review, 94% were conducted in middle-income countries (evenly split between lower- and upper-middle) and 6% in LICs. UMICs were slightly overrepresented compared to their share of the LMIC population (approximately 40%). Only 8 of the studies were multicountry studies, suggesting that cross-national generalizability is not the primary motivation for this type of research. India and China, with 43% of the population of LMICs, comprised one-third of the studies. South Africa, Brazil, Iran, Kenya, and Nigeria, were well represented, each contributing more than 3% of the research.

The studies described 265 different NCD interventions, ranging from screening to prevention to treatment and palliation. Conditions studied varied substantially by region. All 13 of the interventions studied in LICs were for cervical cancer screening. In low-middle income countries, cervical cancer accounted for 37%, diabetes for 29%, and hypertension for 8% of interventions. There was a larger variety of conditions studied in UMICs: while cervical cancer and diabetes comprised half the studies, hypertension, myocardial infarction, colorectal cancer, other cardiovascular diseases, and unhealthy diet each comprised more than 5% of studies. The 2 countries with the largest research output and populations, China and India, differed substantially in focus. In India over 70% of studies were on 2 conditions: diabetes (51%) and cervical cancer (19%), whereas the research was more evenly distributed across the NCDs in China.

Half of all studied interventions in this review evaluated screening for disease, nearly 40% treatment and 12% prevention. Over 70% of all screening studies were for cervical cancer, with less research on other conditions for which screening can be cost effective, such as diabetes, colorectal cancer, and breast cancer. Primary and secondary prevention can reduce incidence of disease and forestall disease progression and disability. We found that only 31 (12%) of the studied interventions addressed prevention with nearly 80% of these tackling prevention of the NCD behavior risk factors (e.g., tobacco use, inactivity, unhealthy diet). Less than 10% of the interventions evaluated in this review focused on management of hypertension (the leading metabolic risk factor worldwide, accounting for approximately 19% of global deaths) [246]. This suggests a substantial implementation research gap in secondary prevention, a critical function of primary care and other levels of health systems. Primary care services such as hypertension management and glucose control play a major role in reducing mortality, thus insufficient research on their optimal implementation is a major missed opportunity. Recent work shows that treatment and control rates for hypertension were below 25% and 10%, respectively, in many countries in South Asia and sub-Saharan Africa. These countries also showed the slowest rates of improvement from 1990 [247].

The preponderance of interventions studied was in pilot phase, with fewer than 15% studying large-scale implementation. Along the same lines, feasibility and adoption were the most studied implementation outcomes, suggesting the research is focused on introduction of new approaches. While proof of concept studies is vital with new implementation strategies, arguably WHO-proposed interventions are well established and evidence on (clinical) effectiveness abound. To provide useful guidance to health system planners and realize population health gains, there needs to be a greater investment in large-scale NCD implementation research to promote sustainability of evidence-based interventions. To best scale scarce research resources and accelerate impact, countries could join regional consortia to study interventions and undertake factorial designs that compare locally adapted implementation approaches.

Over three-quarters of the studies were situated at the micro level of the health system—targeting patient, provider, or clinic levels. Nearly 1 in 5 tested a new technology, despite evidence that technology adoption without substantial integration into policies, data, and workflows is typically ineffective in transforming care [248,249]. Education was another common target featuring in 3 of 10 studies; researchers accounted for the majority of the actors. While micro-level approaches are the most “researchable,” as they are easiest to implement and analyze; positive results are difficult to scale and sustain in the absence of systemic health system change. The Lancet Commission on High Quality Health Systems notes that high-quality care results from structures that align system aims and policies with strong governance, management, and appropriately trained workforce [250]. In this context, micro-level innovation cannot raise quality system wide and is only effective if undertaken as part of a learning health system that can determine whether it offers sufficient benefit over current practice in complexity, cost, and health benefit, and if so, how to best integrate into the health system [251].

Nearly 9 in 10 studies were stand-alone implementation research. This also points to an opportunity to add implementation research to ongoing effectiveness trials. Integrated or hybrid effectiveness-implementation studies are increasingly being used in high-income countries to shed light on both the outcome and extent and quality of service/program delivery [252]. Notably, fewer than 5% of studies cited use of an implementation science framework consistent with prior research showing that the use of implementation science framework is substantially lower in LMICs compared with high-income countries [253]. The use of a tested conceptual framework can improve the rigor of the research and promote comparability of results. Of the studies that reported a funding source, 60% was from international sources, 33% from the country of the research, and the remaining from both local and international sources. This reflects the low spending for health research and especially for health systems and implementation research in LMICs. The lack of domestic support is unlikely to be offset by global funding going forward; a recent analysis showed that NCDs were under prioritized in bilateral agency portfolios relative to their health impacts [254]. Over 40% of development assistance for health in LICs for NCDs came from NGOs and philanthropies, which are less inclined to support research than operations [254]. Indeed, we found that only 16% of studies with funding information reviewed were supported by philanthropies or NGOs, while the other remaining studies reported funding sources from government, private, and/or multiple sources.

Scarcity of funding for research is a key constraint to needed implementation research for NCDs. While there are proposals for coordinating and increasing global support, it is unrealistic to expect this to meet the scale of needed research without a substantial increase in countries’ investment in research [255]. Such an investment is likely to pay off in better health and higher quality, more efficient service delivery [256]. To make best use of research funds, implementation science should strive to be as generalizable as possible—at minimum at a regional level where health systems share similarities. International and regional institutions can play an important role in supporting research consortia and partnerships to promote efficiency of and accelerate the pace of research and, ultimately its uptake into routine care at scale.

Over 50 of the 222 included studies used an experimental research design. While this is the strongest design to yield causal inference, it is not always feasible to implement. Quasi-experimental designs, such as pretest, posttest comparison group designs, and interrupted time series, which can offer robust information were used in only 15 studies. Preexperimental designs that do not include a comparison group or tracking over time, comprised nearly a quarter of the studies. These designs have very low internal validity and should generally be avoided. The remainder of the studies used cross-sectional descriptions, cohort studies, and qualitative research or multiple study types. Given the disproportionate health harms of NCDs among the poor and other vulnerable groups within countries, disaggregated or stratified analysis is crucial. Forty percent of the assessed studies included stratification by age, sex, education, or urbanicity. Going forward, greater use of quasi-experimental designs, hybrid implementation studies and mixed methods approaches, would benefit the field. An expanded focus on equity of implementation outcomes is also needed.

### Strengths and limitations

Our study had several strengths, notably the extensive scope for the search that covered LMICs, a wide range of outcomes and study types, and a large contingent of conditions and health services. We had no language restrictions permitting a comprehensive assessment of the published literature. The review also had several limitations. We focused on WHO-recommended interventions, which at present do not include guidance for some prevalent conditions such as mental health problems and kidney disease [6,12]. Mental health is a major contributor to the global burden of disease and future work should assess the implementation science for the growing range of mental health interventions that appear to be effective in lower-income settings [257,258]. The studies we assessed used differing definitions of implementation outcomes (e.g., acceptability was measured in some studies by self-report and in others by behavior change). This limits direct comparison of study outcomes. Greater use of implementation science frameworks can promote coherence in the research approaches and terminology used to the benefit of end users. Similarly, given the implementation strategies were not specified well enough in the included studies, we elected to focus on actors, action target, and recipients in our description of implementation strategies. Clearly, reporting empirical implementation studies using existing framework to describe implementation strategies would help bolster uptake of implementation research in NCDs.

We also did not search the gray literature and as such, some relevant studies may have been missed. However, studies in gray literature that were not peer reviewed would have not have been eligible for inclusion in this review. Despite using rigorous search strategies without language restrictions, studies published in journals not indexed in MEDLINE and EMBASE were not captured [259–263]. Given the focus on this review and the heterogeneity in aims and methodologies of included studies, risk of bias assessment to understand how effect size may have been compromised by bias is not applicable. As such, we only commented on the distribution of research designs and discussed about stronger/weaker designs. Lastly, we reported year of publication and not time of when study/implementation was conducted.

## Conclusions

High-quality implementation science can play a key role in informing effective delivery of health system interventions to mitigate the burden of NCDs and avoiding expensive mistakes. While implementation research on priority NCDs has grown substantially, from under 10 studies per year in early 2000s to 51 studies in 2020, this is still vastly incommensurate with the health importance of the topic. Further, the concentration of studies in a few geographies and a few health areas, such as cervical cancer, highlights the dearth of research for other key conditions. We found a major gap in research on secondary prevention, i.e., management of risk factors or early disease to prevent disease progression and premature death. Research on ways in which health systems can be strengthened, including primary care levels, to provide optimal care for NCDs is critically needed. Future studies should use implementation science frameworks, and, when testing interventions, strong research designs with strong internal validity, including well-designed quasi-experimental studies. Opportunities exist for adding implementation science studies to planned effectiveness research.

## Supporting information

S1 PRISMA Checklist PRISMA 2020 checklist. (DOCX)

S1 Appendix Appendix tables and figures. Table A in S1 Appendix. Interventions provided within health systems. Table B in S1 Appendix. Sample of the search strategy used in the MEDLINE database. Table C in S1 Appendix. List of low- and middle-income countries. Table D in S1 Appendix. Data extraction tool. Table E in S1 Appendix. Distribution of studies by countries where they were implemented. Fig A in S1 Appendix. Variation of conditions evaluated by income group. Fig B in S1 Appendix. Priority NCD interventions (*n* = 265) identified in 222 studies included in the review. Fig C in S1 Appendix. Distribution of included studies by NCD. Fig D in S1 Appendix. Distribution of intervention type by income group. Fig E in S1 Appendix. Distributions by research designs. Fig F in S1 Appendix. Distributions by standalone implementation studies vs. embedded or hybrid effectiveness-implementation studies. Fig G in S1 Appendix. Distributions by pilot vs. scale-up project. Fig H in S1 Appendix. Variation by level of health system. Fig I in S1 Appendix. Studies that reported funding (vs. those that did not) by NCD conditions. Fig J in S1 Appendix. Distributions by funding type. Fig K in S1 Appendix. Distribution of funding sources by NCDs and their risk factors. Fig L in S1 Appendix. Types of reported funding sources (*N* = 222 included studies).
(DOCX)

## Abbreviation

COVID-19: Coronavirus Disease 2019
LIC: low-income country
LMIC: low- and middle-income country
NCD: noncommunicable disease
SDG: Sustainable Development Goal
UMIC: upper middle-income country

## References

[1] World Health Organization. Noncommunicable diseases: World Health Organization. 2021 [cited 2021 Sep 7]. Available from: https://www.who.int/news-room/fact-sheets/detail/noncommunicable-diseases.

[2] World Health Organization. Global health estimates. 2020.

[3] Countdown NCD. NCD Countdown 2030: pathways to achieving Sustainable Development Goal target 3.4. Lancet. 2020. doi: 10.1016/S0140-6736(20)31761-X 32891217 PMC7470795

[4] World Health Organization. Assessing national capacity for the prevention and control of noncommunicable diseases. Report of 2019 Global Survey WHO. 2020.

[5] Bukhman G, Mocumbi AO, Atun R, Becker AE, Bhutta Z, Binagwaho A, et al. The Lancet LancetNCDI Poverty Commission: bridging a gap in universal health coverage for the poorest billion. Lancet. 2020;396(10256):991–1044. Epub 2020/09/18. doi: 10.1016/S0140-6736(20)31907-3 ; PubMed Central PMCID: PMC7489932. 32941823 PMC7489932

[6] World Health Organization. Updated appendix 3 of the who global Ncd action plan 2013–2020. Technical Annex 2017.

[7] World Health Organization. Global action plan for the prevention and control of noncommunicable diseases 2013–2020: World Health Organization. 2013.

[8] Marten R, Mikkelsen B, Shao R, Dal Zennaro L, Berdzuli N, Fernando T, et al. Committing to implementation research for health systems to manage and control non-communicable diseases. Lancet Glob Health. 2021;9(2):e108–e109. doi: 10.1016/S2214-109X(20)30485-X 33357502 PMC7815625

[9] World Health Organization. Mid-point evaluation of the implementation of the WHO global action plan for the prevention and control of noncommunicable diseases 2013–2020 (NCD-GAP). 2020.

[10] Alonge O, Rodriguez DC, Brandes N, Geng E, Reveiz L, Peters DH. How is implementation research applied to advance health in low-income and middle-income countries? BMJ Glob Health. 2019;4(2):e001257. doi: 10.1136/bmjgh-2018-001257 30997169 PMC6441291

[11] Kruk ME, Gage AD, Arsenault C, Jordan K, Leslie HH, Roder-DeWan S, et al. High-quality health systems in the Sustainable Development Goals era: time for a revolution. Lancet Glob Health. 2018;6(11):e1196–e1252. doi: 10.1016/S2214-109X(18)30386-3 30196093 PMC7734391

[12] Isaranuwatchai W, Teerawattananon Y, Archer RA, Luz A, Sharma M, Rattanavipapong W, et al. Prevention of non-communicable disease: best buys, wasted buys, and contestable buys. BMJ. 2020;368. 10.1136/bmj.m141 PMC7190374 31992592

[13] Madon T, Hofman KJ, Kupfer L, Glass RI. Public Health: Implementation Science. Science. 2007;318(5857):1728–1729. doi: 10.1126/science.1150009 18079386

[14] Geng EH, Peiris D, Kruk ME. Implementation science: Relevance in the real world without sacrificing rigor. PLoS Med. 2017;14(4):e1002288. Epub 2017/04/26. doi: 10.1371/journal.pmed.1002288 ; PubMed Central PMCID: PMC5404833. 28441435 PMC5404833

[15] Allen LN, Pullar J, Wickramasinghe KK, Williams J, Roberts N, Mikkelsen B, et al. Evaluation of research on interventions aligned to WHO ‘Best Buys’ for NCDs in low-income and lower-middle-income countries: a systematic review from 1990 to 2015. BMJ Glob Health. 2018;3(1):e000535. doi: 10.1136/bmjgh-2017-000535 29527342 PMC5841523

[16] Allen LN, Nicholson BD, Yeung BY, Goiana-da-Silva F. Implementation of non-communicable disease policies: a geopolitical analysis of 151 countries. Lancet Glob Health. 2020;8(1):e50–e58. doi: 10.1016/S2214-109X(19)30446-2 31813787 PMC7024987

[17] Allen LN, Wigley S, Holmer H. Implementation of non-communicable disease policies from 2015 to 2020: a geopolitical analysis of 194 countries. Lancet Glob Health. 2021;9(11):e1528–e1538. doi: 10.1016/S2214-109X(21)00359-4 34678197

[18] Systematic Review of NCD Implementation Research in Low- and Middle-Income Countries. [Internet]. PROSPERO 2021. [cited 2021 Oct 2]. Available from: https://www.crd.york.ac.uk/prospero/display_record.php?ID=CRD42021252969.

[19] Moher D, Liberati A, Tetzlaff J, Altman DG. Preferred reporting items for systematic reviews and meta-analyses: the PRISMA statement. Ann Intern Med. 2009;151(4):264–269. doi: 10.7326/0003-4819-151-4-200908180-00135 19622511

[20] Proctor E, Silmere H, Raghavan R, Hovmand P, Aarons G, Bunger A, et al. Outcomes for implementation research: conceptual distinctions, measurement challenges, and research agenda. Adm Policy Ment Health. 2011;38(2):65–76. doi: 10.1007/s10488-010-0319-7 20957426 PMC3068522

[21] Glasgow RE, Vogt TM, Boles SM. Evaluating the public health impact of health promotion interventions: the RE-AIM framework. Am J Public Health. 1999;89(9):1322–1327. doi: 10.2105/ajph.89.9.1322 10474547 PMC1508772

[22] Veritas Health Innovation. Covidence systematic review software. 2020 [cited 2021 Nov 5]. Available from: https://get.covidence.org/systematic-review?campaignid=11343712044&adgroupid=114130876511&gclid=Cj0KCQjwtrSLBhCLARIsACh6RmjH6RwcJalTVCaL76kpSCeqErC2sP8uX4Vj8wZYdEQWQxdXWVwD0eMaAsz1EALw_wcB.

[23] Proctor EK, Powell BJ, McMillen JC. Implementation strategies: recommendations for specifying and reporting. Implement Sci. 2013;8(1):1–11. doi: 10.1186/1748-5908-8-139 24289295 PMC3882890

[24] Abdullah F, Su TT. Enhancement of the cervical cancer screening program in Malaysia: a qualitative study. Asian Pac J Cancer Prev. 2010;11(5):1359–66. . 21198293

[25] Abdullah NN, Daud S, Wang SM, Mahmud Z, Mohd Kornain NK, Al-Kubaisy W. Human Papilloma Virus (HPV) self-sampling: do women accept it? J Obstet Gynaecol. 2018;38(3):402–7. doi: 10.1080/01443615.2017.1379061 . 29385850

[26] Abu SH, Woldehanna BT, Nida ET, Tilahun AW, Gebremariam MY, Sisay MM. The role of health education on cervical cancer screening uptake at selected health centers in Addis Ababa. PLoS ONE [Electronic Resource]. 2020;15(10):e0239580. doi: 10.1371/journal.pone.0239580 . 33027267 PMC7540882

[27] Abuadas FH, Petro-Nustas WJ, Abuadas MH. The Effect of a Health Education Intervention on Jordanian Participants’ Colorectal Cancer Knowledge, Health Perceptions, and Screening Practices. Cancer Nurs. 2018;41(3):226–37. doi: 10.1097/NCC.0000000000000480 . 28252461

[28] Abuelo CE, Levinson KL, Salmeron J, Sologuren CV, Fernandez MJ, Belinson JL. The Peru Cervical Cancer Screening Study (PERCAPS): the design and implementation of a mother/daughter screen, treat, and vaccinate program in the Peruvian jungle. J Community Health. 2014;39(3):409–15. doi: 10.1007/s10900-013-9786-6 . 24276617 PMC4543313

[29] Adefuye PO, Dada OA, Adefuye BO, Shorunmu TO, Akinyemi BO, Idowu-Ajiboye BO. Feasibility, acceptability, and effectiveness of visual inspection of the cervix with acetic acid and cryotherapy for dysplasia in Nigeria. Int J Gynaecol Obstet. 2015;129(1):62–6. doi: 10.1016/j.ijgo.2014.10.032 . 25593107

[30] Adsul P, Srinivas V, Gowda S, Nayaka S, Pramathesh R, Chandrappa K, et al. A community-based, cross-sectional study of hrHPV DNA self-sampling-based cervical cancer screening in rural Karnataka, India. Int J Gynaecol Obstet. 2019;146(2):170–6. doi: 10.1002/ijgo.12859 . 31074835 PMC6620767

[31] Ajay VS, Jindal D, Roy A, Venugopal V, Sharma R, Pawar A, et al. Development of a Smartphone-Enabled Hypertension and Diabetes Mellitus Management Package to Facilitate Evidence-Based Care Delivery in Primary Healthcare Facilities in India: The mPower Heart Project. J Am Heart Assoc. 2016;5(12). doi: 10.1161/JAHA.116.004343 . 28003248 PMC5210443

[32] Alexander T, Mullasari AS, Joseph G, Kannan K, Veerasekar G, Victor SM, et al. A system of care for patients with ST-segment elevation myocardial infarction in India: The Tamil Nadu-ST-segment elevation myocardial infarction program. JAMA Cardiol. 2017;2(5):498–505. doi: 10.1001/jamacardio.2016.5977 . 28273293 PMC5814984

[33] Alfaro K, Maza M, Felix JC, Gage JC, Castle PE, Alonzo TA, et al. Outcomes for Step-Wise Implementation of a Human Papillomavirus Testing-Based Cervical Screen-and-Treat Program in El Salvador. JCO Glob Oncol. 2020;6:1519–30. doi: 10.1200/GO.20.00206 . 33064628 PMC7605377

[34] Allende G, Surriabre P, Caceres L, Bellot D, Ovando N, Torrico A, et al. Evaluation of the self-sampling for cervical cancer screening in Bolivia. BMC Public Health. 2019;19(1):80. doi: 10.1186/s12889-019-6401-5 . 30654774 PMC6337790

[35] Allerton J, Mash R. The impact of intensified clinical care on glycaemic control in patients with type 2 diabetes at Khayelitsha Community Health Centre, South Africa: Quasi-experimental study. Prim Care Diabetes. 2020;14(2):97–103. doi: 10.1016/j.pcd.2019.08.006 . 31564516

[36] Amir Khan M, Ahmar Khan M, Walley JD, Khan N, Imtiaz Sheikh F, Ali S, et al. Feasibility of delivering integrated COPD-asthma care at primary and secondary level public healthcare facilities in Pakistan: a process evaluation. BJGP Open. 2019;3(1):bjgpopen18X101632. doi: 10.3399/bjgpopen18X101632 . 31049412 PMC6480853

[37] Arief M, Harika B, Nilugal K, Syed IA. Clinical pharmacist interventions in managing cardiovascular risk factors. Int J Pharm Sci Rev Res. 2015;35(1):63–6.

[38] Arrossi S, Thouyaret L, Herrero R, Campanera A, Magdaleno A, Cuberli M, et al. Effect of self-collection of HPV DNA offered by community health workers at home visits on uptake of screening for cervical cancer (the EMA study): a population-based cluster-randomised trial. Lancet Glob Health. 2015;3(2):e85–94. doi: 10.1016/S2214-109X(14)70354-7 . 25617202

[39] Asgary R, Adongo PB, Nwameme A, Cole HV, Maya E, Liu M, et al. mHealth to Train Community Health Nurses in Visual Inspection With Acetic Acid for Cervical Cancer Screening in Ghana. J Low Genit Tract Dis. 2016;20(3):239–42. doi: 10.1097/LGT.0000000000000207 . 27030884 PMC4920727

[40] Austad K, Chary A, Xocop SM, Messmer S, King N, Carlson L, et al. Barriers to Cervical Cancer Screening and the Cervical Cancer Care Continuum in Rural Guatemala: A Mixed-Method Analysis. J Glob Oncol. 2018;4:1–10. doi: 10.1200/JGO.17.00228 . 30084698 PMC6223515

[41] Awolude OA, Oyerinde SO, Akinyemi JO. Screen and triage to facilitate screen and treat by community health extension workers: Task-sharing strategy to achieve universal cervical cancer screening in Nigeria. J Glob Oncol. 2018;4(Supplement 1):14S. doi: 10.1200/JGO.18.00023 . 30085882 PMC6223525

[42] Aye LL, Tripathy JP, Maung Maung T, Oo MM, Nwe ML, Thu HMM, et al. Experiences from the pilot implementation of the Package of Essential Non-communicable Disease Interventions (PEN) in Myanmar, 2017–18: A mixed methods study. PLoS ONE [Electronic Resource]. 2020;15(2):e0229081. doi: 10.1371/journal.pone.0229081 . 32069323 PMC7028297

[43] Aziz Z, Mathews E, Absetz P, Sathish T, Oldroyd J, Balachandran S, et al. A group-based lifestyle intervention for diabetes prevention in low- and middle-income country: implementation evaluation of the Kerala Diabetes Prevention Program. Implement Sc. 2018;13(1):97. doi: 10.1186/s13012-018-0791-0 . 30021592 PMC6052531

[44] Balagopal P, Kamalamma N, Patel TG, Misra R. A community-based diabetes prevention and management education program in a rural village in India. Diabetes Care. 2008;31(6):1097–104. doi: 10.2337/dc07-1680 . 18316397

[45] Bansil P, Wittet S, Lim JL, Winkler JL, Paul P, Jeronimo J. Acceptability of self-collection sampling for HPV-DNA testing in low-resource settings: a mixed methods approach. BMC Public Health. 2014;14:596. doi: 10.1186/1471-2458-14-596 . 24927941 PMC4061776

[46] Bao HL, Zhao ZP, Zhang M, Wang LM, Cong S, Fang LW, et al. [The impact of five-year Chinese rural area cervical cancer screening program on screening rate]. Chung-Hua Yu Fang i Hsueh Tsa Chih [Chinese Journal of Prev Med]. 2018;52(3):260–4. doi: 10.3760/cma.j.issn.0253-9624.2018.03.008 . 29973004

[47] Barasheh N, Shakerinejad G, Nouhjah S, Haghighizadeh MH. The effect of educational program based on the precede-proceed model on improving self-care behaviors in a semi-urban population with type 2 diabetes referred to health centers of Bavi, Iran. Diabetes Metab Syndr. 2017;11(Supplement 2):S759–S65. 28669595 10.1016/j.dsx.2017.05.012

[48] Barfar E, Rashidian A, Hosseini H, Nosratnejad S, Barooti E, Zendehdel K. Cost-effectiveness of mammography screening for breast cancer in a low socioeconomic group of Iranian women. Arch Iran Med. 2014;17(4):241–5. . 24724599

[49] Berner A, Hassel SB, Tebeu PM, Untiet S, Kengne-Fosso G, Navarria I, et al. Human papillomavirus self-sampling in Cameroon: women’s uncertainties over the reliability of the method are barriers to acceptance. J Low Genit Tract Dis. 2013;17(3):235–41. doi: 10.1097/LGT.0b013e31826b7b51 . 23422643

[50] Bernstein M, Hari A, Aggarwal S, Lee D, Farfel A, Patel P, et al. Implementation of a human papillomavirus screen-and-treat model in Mwanza, Tanzania: training local healthcare workers for sustainable impact. Int Health. 2018;10(3):197–201. doi: 10.1093/inthealth/ihy014 . 29579207

[51] Bhatt S, Isaac R, Finkel M, Evans J, Grant L, Paul B, et al. Mobile technology and cancer screening: Lessons from rural India. J Glob Health. 2018;8(2):020421. doi: 10.7189/jogh.08.020421 . 30603075 PMC6304168

[52] Blumenthal PD, Gaffikin L, Deganus S, Lewis R, Emerson M, Adadevoh S, et al. Cervical cancer prevention: safety, acceptability, and feasibility of a single-visit approach in Accra, Ghana. Am J Obstet Gynecol. 2007;196(4):407.e1–8; discussion. e8. doi: 10.1016/j.ajog.2006.12.031 . 17403438

[53] Botha MH, Van Der Merwe FH, Snyman L, Dreyer G. The vaccine and cervical cancer screen (VACCS) project-acceptance of human papilloma virus vaccination in a school based program. Int J Gynecol Cancer. 2014;24(9):821. .26046162

[54] Bouchlaka A, Ben Abdallah M, Ben Aissa R, Smida S, Ouechtati A, Boussen H, et al. [Practice of large scale mammography in the Ariana area of Tunisia: prelude to a mass screening?]. Tunis Med. 2009;87(7):426–31. . 20063674

[55] Broquet C, Triboullier D, Untiet S, Schafer S, Petignat P, Vassilakos P. Acceptability of self-collected vaginal samples for HPV testing in an urban and rural population of Madagascar. Afr Health Sci. 2015;15(3):755–61. doi: 10.4314/ahs.v15i3.8 . 26957962 PMC4765472

[56] Busingye P, Nakimuli A, Nabunya E, Mutyaba T. Acceptability of cervical cancer screening via visual inspection with acetic acid or Lugol’s iodine at Mulago Hospital, Uganda. Int J Gynaecol Obstet. 2012;119(3):262–5. doi: 10.1016/j.ijgo.2012.06.015 . 22980432

[57] Cai SR, Zhu HH, Huang YQ, Li QL, Ma XY, Zhang SZ, et al. Cost-Effectiveness between Double and Single Fecal Immunochemical Test(s) in a Mass Colorectal Cancer Screening. Biomed Res Int. 2016;2016:6830713. doi: 10.1155/2016/6830713 . 27144171 PMC4838800

[58] Campbell C, Kafwafwa S, Brown H, Walker G, Madetsa B, Deeny M, et al. Use of thermo-coagulation as an alternative treatment modality in a ‘screen-and-treat’ programme of cervical screening in rural Malawi. Int J Cancer. 2016;139(4):908–15. doi: 10.1002/ijc.30101 . 27006131 PMC5084797

[59] Chalapati W, Chumworathayi B. Can a home-visit invitation increase Pap smear screening in Samliem, Khon Kaen, Thailand? Asian Pac J Cancer Prev. 2007;8(1):119–23. . 17477785

[60] Chariwala RA, Shukla R, Gajiwala UR, Gilbert C, Pant H, Lewis MG, et al. Effectiveness of health education and monetary incentive on uptake of diabetic retinopathy screening at a community health center in South Gujarat, India. Indian J Ophthalmol. 2020;68(Suppl 1):S52–S5. doi: 10.4103/ijo.IJO_2118_19 . 31937730 PMC7001183

[61] Chary AN, Rohloff PJ. Major challenges to scale up of visual inspection-based cervical cancer prevention programs: the experience of Guatemalan NGOs. Glob Health Sci Pract. 2014;2(3):307–17. doi: 10.9745/GHSP-D-14-00073 . 25276590 PMC4168631

[62] Chigbu C, Onyebuchi A, Onyeka T, Odugu B, Dim C. Impact of community health educators on uptake of cervical/ breast cancer prevention services. Int J Gynaecol Obstet. 2018;143(Supplement 3):443. . 28295268 10.1002/ijgo.12150

[63] Chinula L, Mapanje C, Varela A, Chapola J, Limarzi L, Bulla A, et al. Uptake of a community-based screen-and-treat cervical cancer prevention strategy in rural malawi. Int J Gynaecol Obstet. 2018;143(Supplement 3):456. .33687746

[64] Chong HY, Roslani AC, Law CW. Colonoscopic prioritization in colorectal carcinoma screening using quantitative immunochemical faecal occult blood test: a pilot study. Med J Malaysia. 2013;68(1):30–3. . 23466763

[65] Chumworathayi B, Yuenyao P, Luanratanakorn S, Pattamadilok J, Chalapati W, Na-Nhongkai C. Can an appointment-letter intervention increase pap smear screening in Samliem, Khon Kaen, Thailand? Asian Pac J Cancer Prev. 2007;8(3):353–6. . 18159966

[66] Chutinet A, Keosodsay S, Vorasayan P, Samajarn J, Akarathanawat W, Kijpaisalratana N, et al. The First 10 Thrombolysis for Acute Ischemic Stroke in Lao People’s Democratic Republic under Teleconsultation from Thailand. J Stroke Cerebrovasc Dis. 2019;28(11):104327. doi: 10.1016/j.jstrokecerebrovasdis.2019.104327 . 31530479

[67] Crofts V, Flahault E, Tebeu PM, Untiet S, Fosso GK, Boulvain M, et al. Education efforts may contribute to wider acceptance of human papillomavirus self-sampling. Int J Womens Health. 2015;7:149–54. doi: 10.2147/IJWH.S56307 . 25674016 PMC4321569

[68] Dandge S, Jeemon P, Reddy PS. Technology enabled non-physician health workers extending telemedicine to rural homes to control hypertension and diabetes (TETRA): A pre-post demonstration project in Telangana, India. PLoS ONE. 2019;14(2). doi: 10.1371/journal.pone.0211551 . 30779798 PMC6380577

[69] de Villiers A, Steyn NP, Draper CE, Hill J, Dalais L, Fourie J, et al. Implementation of the HealthKick intervention in primary schools in low-income settings in the Western Cape Province, South Africa: a process evaluation. BMC Public Health. 2015;15:818. doi: 10.1186/s12889-015-2157-8 . 26297447 PMC4546332

[70] Do TNP, Do QH, Cowie MR, Ha NB, Do VD, Do TH, et al. Effect of the Optimize Heart Failure Care Program on clinical and patient outcomes—The pilot implementation in Vietnam. Int J Cardiol Heart Vasc. 2019;22:169–73. doi: 10.1016/j.ijcha.2019.02.010 . 30899774 PMC6409388

[71] Dorje T, Zhao G, Tso K, Wang J, Chen Y, Tsokey L, et al. Smartphone and social media-based cardiac rehabilitation and secondary prevention in China (SMART-CR/SP): a parallel-group, single-blind, randomised controlled trial. Lancet Digit Health. 2019;1(7):e363–e74. doi: 10.1016/S2589-7500(19)30151-7 . 33323210

[72] Dorji T, Tshomo U, Phuntsho S, Tamang TD, Tshokey T, Baussano I, et al. Introduction of a National HPV vaccination program into Bhutan. Vaccine. 2015;33(31):3726–30. doi: 10.1016/j.vaccine.2015.05.078 . 26057136

[73] Elnaem MH, Nik Mohamed MH, Huri HZ. Pharmacist-led academic detailing improves statin therapy prescribing for Malaysian patients with type 2 diabetes: Quasi-experimental design. PLoS ONE [Electronic Resource]. 2019;14(9):e0220458. doi: 10.1371/journal.pone.0220458 . 31536502 PMC6752830

[74] Erwin E, Aronson KJ, Day A, Ginsburg O, Macheku G, Feksi A, et al. SMS behaviour change communication and eVoucher interventions to increase uptake of cervical cancer screening in the Kilimanjaro and Arusha regions of Tanzania: a randomised, double-blind, controlled trial of effectiveness. BMJ Innov. 2019;5(1):28–34. doi: 10.1136/bmjinnov-2018-000276 . 31645991 PMC6792319

[75] Escosteguy CC, Teixeira AB, Portela MC, Guimaraes AE, Lima SM, Ferreira VM, et al. Implementing clinical guidelines on acute myocardial infarction care in an emergency service. Arq Bras Cardiol. 2011;96(1):18–25. doi: 10.1590/s0066-782x2010005000142 . 21109914

[76] Fall NS, Tamalet C, Diagne N, Fenollar F, Raoult D, Sokhna C, et al. Feasibility, Acceptability, and Accuracy of Vaginal Self-Sampling for Screening Human Papillomavirus Types in Women from Rural Areas in Senegal. Am J Trop Med Hyg. 2019;100(6):1552–5. doi: 10.4269/ajtmh.19-0045 . 30994102 PMC6553900

[77] Fallala MS, Mash R. Cervical cancer screening: Safety, acceptability, and feasibility of a single-visit approach in Bulawayo, Zimbabwe. Afr J Prim Health Care Fam Med. 2015;7(1):05. doi: 10.4102/phcfm.v7i1.742 . 26245601 PMC4564888

[78] Flor LS, Wilson S, Bhatt P, Bryant M, Burnett A, Camarda JN, et al. Community-based interventions for detection and management of diabetes and hypertension in underserved communities: a mixed-methods evaluation in Brazil, India, South Africa and the USA. BMJ Glob Health. 2020;5(6):06. doi: 10.1136/bmjgh-2019-001959 . 32503887 PMC7279660

[79] Fokom Domgue J, Futuh B, Ngalla C, Kakute P, Manjuh F, Manga S, et al. Feasibility of a community-based cervical cancer screening with “test and treat” strategy using self-sample for an HPV test: Experience from rural Cameroon, Africa. Int J Cancer. 2020;147(1):128–38. doi: 10.1002/ijc.32746 . 31633801

[80] Fong J, Gyaneshwar R, Lin S, Morrell S, Taylor R, Brassil A, et al. Cervical screening using visual inspection with acetic acid (VIA) and treatment with cryotherapy in Fiji. Asian Pac J Cancer Prev. 2014;15(24):10757–62. doi: 10.7314/apjcp.2014.15.24.10757 . 25605171

[81] Fort MP, Murillo S, Lopez E, Dengo AL, Alvarado-Molina N, de Beausset I, et al. Impact evaluation of a healthy lifestyle intervention to reduce cardiovascular disease risk in health centers in San Jose, Costa Rica and Chiapas, Mexico. BMC Health Serv Res. 2015;15:577. doi: 10.1186/s12913-015-1248-7 . 26711290 PMC4693408

[82] Gagliardino JJ, Etchegoyen G, Pendid-La Research G. A model educational program for people with type 2 diabetes: a cooperative Latin American implementation study (PEDNID-LA). Diabetes Care. 2001;24(6):1001–7. doi: 10.2337/diacare.24.6.1001 . 11375360

[83] Geng W, Tian X, Fu X, Wang P, Wang Y, Wang X, et al. Early routine angioplasty versus selective angioplasty after successful thrombolysis in acute ST-segment elevation myocardial infarction. Coron Artery Dis. 2013;24(3):238–43. doi: 10.1097/MCA.0b013e32835e5c67 . 23358446

[84] Ghosh K, Segura A, Crispen C, Montz FJ. Use of the ‘see and treat’ technique for the management of high-risk abnormal Pap smears in a Third World country. Int J Gynecol Cancer. 1997;7(2):144–50.

[85] Gottschlich A, Rivera-Andrade A, Grajeda E, Alvarez C, Mendoza Montano C, Meza R. Acceptability of Human Papillomavirus Self-Sampling for Cervical Cancer Screening in an Indigenous Community in Guatemala. J Glob Oncol. 2017;3(5):444–54. doi: 10.1200/JGO.2016.005629 . 29094082 PMC5646882

[86] Greenwald ZR, Fregnani JH, Longatto-Filho A, Watanabe A, Mattos JSC, Vazquez FL, et al. The performance of mobile screening units in a breast cancer screening program in Brazil. Cancer Causes Control. 2018;29(2):233–41. doi: 10.1007/s10552-017-0995-7 . 29250701

[87] Gulayin PE, Lozada A, Beratarrechea A, Gutierrez L, Poggio R, Chaparro RM, et al. An Educational Intervention to Improve Statin Use: Cluster RCT at the Primary Care Level in Argentina. Am J Prev Med. 2019;57(1):95–105. doi: 10.1016/j.amepre.2019.02.018 . 31128958

[88] Gurav SK, Zirpe KG, Wadia RS, Naniwadekar A, Pote PU, Tungenwar A, et al. Impact of “Stroke Code”-Rapid Response Team: An Attempt to Improve Intravenous Thrombolysis Rate and to Shorten Door-to-Needle Time in Acute Ischemic Stroke. Indian J Crit Care Med. 2018;22(4):243–8. doi: 10.4103/ijccm.IJCCM_504_17 . 29743763 PMC5930528

[89] Haikel RL, Mauad EC, Silva TB, Mattos J, Chala LF, Longatto-Filho A, et al. Mammography-based screening program: Preliminary results from a first 2-year round in a Brazilian region using mobile and fixed units. BMC Womens Health. 2012;12(no pagination). . 23031787 10.1186/1472-6874-12-32 PMC3532077

[90] Hasandokht T, Farajzadegan Z, Siadat ZD, Paknahad Z, Rajati F. Lifestyle interventions for hypertension treatment among Iranian women in primary health-care settings: Results of a randomized controlled trial. J Res Med Sci. 2015;20(1):54–61. . 25767523 PMC4354066

[91] Hassan ZM. Mobile phone text messaging to improve knowledge and practice of diabetic foot care in a developing country: Feasibility and outcomes. Int J Nurs Pract. 2017;23(1). doi: 10.1111/ijn.12546 . 28635062

[92] He M, Wang J, Gong L, Dong Q, Ji N, Xing H, et al. Community-based stroke system of care for Chinese rural areas. Stroke. 2014;45(8):2385–90. doi: 10.1161/STROKEAHA.114.006030 . 25005441

[93] Hebert K, Quevedo HC, Gogichaishvili I, Nozadze N, Sagirashvili E, Trahan P, et al. Feasibility of a heart failure disease management program in eastern Europe: Tbilisi, Georgia. Circ Heart Fail. 2011;4(6):763–9. doi: 10.1161/CIRCHEARTFAILURE.111.962431 . 21900187

[94] Heydari G, Jianfar G, Alvanpour A, Hesami Z, Talischi F, Masjedi MR. Efficacy of telephone quit-line for smokers in Iran: 12 months follow up results. Tanaffus. 2011;10(3):42–8. . 25191375 PMC4153163

[95] Hua W, Cao S, Cui J, Maberley D, Matsubara J. Analysis of reasons for noncompliance with laser treatment in patients of diabetic retinopathy. Can J Ophthalmol. 2017;52(Suppl 1):S34–S8. doi: 10.1016/j.jcjo.2017.09.025 . 29074011

[96] Huang S, Hu X, Chen H, Xie D, Gan X, Wu Y, et al. The positive effect of an intervention program on the hypertension knowledge and lifestyles of rural residents over the age of 35 years in an area of China. Hypertens Res. 2011;34(4):503–8. doi: 10.1038/hr.2010.265 . 21248756

[97] Huang X, Liu L, Song Y, Gao L, Zhao M, Bao H, et al. Achieving blood pressure control targets in hypertensive patients of rural China—A pilot randomized trial. Trials. 2020;21(1). doi: 10.1186/s13063-020-04368-1 . 32527283 PMC7291427

[98] Huchko MJ, Ibrahim S, Blat C, Cohen CR, Smith JS, Hiatt RA, et al. Cervical cancer screening through human papillomavirus testing in community health campaigns versus health facilities in rural western Kenya. Int J Gynaecol Obstet. 2018;141(1):63–9. doi: 10.1002/ijgo.12415 . 29197067 PMC7369666

[99] Huchko MJ, Olwanda E, Choi Y, Kahn JG. HPV-based cervical cancer screening in low-resource settings: Maximizing the efficiency of community-based strategies in rural Kenya. Int J Gynaecol Obstet. 2020;148(3):386–91. doi: 10.1002/ijgo.13090 . 31849036 PMC9261489

[100] Ibrahim HO, Stapar D, Mash B. Is screening for microalbuminuria in patients with type 2 diabetes feasible in the Cape Town public sector primary care context? A cost and consequence study. S Afr Fam Pract. 2013;55(4):367–72.

[101] Isaac R, Finkel M, Olver I, Annie IK, Prashanth HR, Subhashini J, et al. Translating evidence into practice in low resource settings: cervical cancer screening tests are only part of the solution in rural India. Asian Pac J Cancer Prev. 2012;13(8):4169–72. doi: 10.7314/apjcp.2012.13.8.4169 . 23098426

[102] Jafar TH, Silva A, Naheed A, Jehan I, Liang F, Assam PN, et al. Control of blood pressure and risk attenuation: a public health intervention in rural Bangladesh, Pakistan, and Sri Lanka: feasibility trial results. J Hypertens. 2016;34(9):1872–81. doi: 10.1097/HJH.0000000000001014 . 27488552

[103] Jahic E. Experience and Outcomes of Primary Percutaneous Coronary Intervention for Patients with ST-Segment Elevation Myocardial Infarction of Tertiary Care Center in Bosnia and Herzegovina. Med Arh. 2017;71(3):183–7. doi: 10.5455/medarh.2017.71.183-187 . 28974830 PMC5585807

[104] Jeyapaul S, Oommen AM, Cherian AG, Marcus TA, Malini T, Prasad JH, et al. Feasibility, uptake and real-life challenges of a rural cervical and breast cancer screening program in Vellore, Tamil Nadu, South India. Indian J Cancer. 2020;02:02. doi: 10.4103/ijc.IJC_271_19 . 33402583

[105] Jin H, Qu Y, Guo ZN, Yan XL, Sun X, Yang Y. Impact of Jilin Province Stroke Emergency Maps on Acute Stroke Care Improvement in Northeast China. Front Neurol [electronic resource]. 2020;11:734. doi: 10.3389/fneur.2020.00734 . 32774322 PMC7387724

[106] Joshi R, Agrawal T, Fathima F, Usha T, Thomas T, Misquith D, et al. Cardiovascular risk factor reduction by community health workers in rural India: A cluster randomized trial. Am Heart J. 2019;216:9–19. doi: 10.1016/j.ahj.2019.06.007 . 31377568 PMC6842688

[107] Juan Z, Rongrong J, Juanjuan L, Jinlei L, Xiawen S, Guijuan D, et al. Study on the effectiveness of implementation: The National Demonstration Areas for Comprehensive Prevention and Control of Non-communicable Diseases. [Chinese]. Zhonghua Liu Xing Bing Xue Za Zhi. 2018;39(4):394–400. doi: 10.3760/cma.j.issn.0254-6450.2018.04.002 . 29699025

[108] Junling G, Yang L, Junming D, Pinpin Z, Hua F. Evaluation of group visits for Chinese hypertensives based on primary health care center. Asia Pac J Public Health. 2015;27(2):NP350–60. doi: 10.1177/1010539512442566 . 22535548

[109] Kamal AK, Khalid W, Muqeet A, Jamil A, Farhat K, Gillani, et al. Making prescriptions “talk” to stroke and heart attack survivors to improve adherence: Results of a randomized clinical trial (The Talking Rx Study). PLoS ONE [Electronic Resource]. 2018;13(12):e0197671. doi: 10.1371/journal.pone.0197671 . 30571697 PMC6301764

[110] Khan N, Walley JD, Khan SE, Hicks J, Sheikh FI, Khan MA, et al. Enhanced hypertension care through private clinics in Pakistan: A cluster randomised trial. BJGP Open. 2019;3(1). doi: 10.3399/bjgpopen18X101617 . 31049404 PMC6480862

[111] Khetan AK, Zullo MZ, Gupta RG, Agarwal SA, Mohan SM, Josephson RJ. Effect of a community health worker based approach to integrated cardiovascular risk factor control in India: A cluster randomized, controlled, parallel-group trial (SEHAT). Eur Heart J. 2018;39(Supplement 1):646–7.

[112] Khuhaprema T, Sangrajrang S, Lalitwongsa S, Chokvanitphong V, Raunroadroong T, Ratanachu-Ek T, et al. Organised colorectal cancer screening in Lampang Province, Thailand: preliminary results from a pilot implementation programme. BMJ Open. 2014;4(1):e003671. doi: 10.1136/bmjopen-2013-003671 . 24435889 PMC3902312

[113] Klassen SL, Miller RJH, Hao R, Warnica JW, Fine NM, Carpen M, et al. Implementation of a Multidisciplinary Inpatient Cardiology Service to Improve Heart Failure Outcomes in Guyana. J Card Fail. 2018;24(12):835–41. doi: 10.1016/j.cardfail.2018.07.002 . 30012360

[114] Ku GM, Kegels G. Effects of the First Line Diabetes Care (FiLDCare) self-management education and support project on knowledge, attitudes, perceptions, self-management practices and glycaemic control: a quasi-experimental study conducted in the Northern Philippines. BMJ Open. 2014;4(8):e005317. doi: 10.1136/bmjopen-2014-005317 . 25113555 PMC4127918

[115] Kundury KK, Hathur B. Intervention through Short Messaging System (SMS) and phone call alerts reduced HbA1C levels in ~47% type-2 diabetics-results of a pilot study. PLoS ONE [Electronic Resource]. 2020;15(11):e0241830. doi: 10.1371/journal.pone.0241830 . 33201926 PMC7671489

[116] Kurt G, Akyuz A. Evaluating the Effectiveness of Interventions on Increasing Participation in Cervical Cancer Screening. J Nurs Res. 2019;27(5):e40. doi: 10.1097/jnr.0000000000000317 . 30908429 PMC6752698

[117] Kushwaha S, Talwar P, Chandel N, Anthony A, Maheshwari S, Khurana S. Saving the brain initiative—Developing an effective hub-and-spoke model to improve the acute stroke management pathways in urban India. J Neurol Sci. 2018;393:83–7. doi: 10.1016/j.jns.2018.08.012 . 30125806

[118] Laatikainen T, Inglin L, Collins D, Ciobanu A, Curocichin G, Salaru V, et al. Implementing Package of Essential Non-communicable Disease Interventions in the Republic of Moldova-a feasibility study. Eur J Public Health. 2020;16. doi: 10.1093/eurpub/ckaa037 . 32298428

[119] Latina J, Fernandez-Jimenez R, Bansilal S, Sartori S, Vedanthan R, Lewis M, et al. Grenada Heart Project-Community Health ActioN to EncouraGe healthy BEhaviors (GHP-CHANGE): A randomized control peer group-based lifestyle intervention. Am Heart J. 2020;220:20–8. doi: 10.1016/j.ahj.2019.08.022 . 31765932

[120] Lee H, Mtengezo JT, Kim D, Makin MS, Kang Y, Malata A, et al. Exploring Complicity of Cervical Cancer Screening in Malawi: The Interplay of Behavioral, Cultural, and Societal Influences. Asia Pac J Oncol Nurs. 2020;7(1):18–27. doi: 10.4103/apjon.apjon_48_19 . 31879680 PMC6927154

[121] Legood R, Gray AM, Mahe C, Wolstenholme J, Jayant K, Nene BM, et al. Screening for cervical cancer in India: How much will it cost? A trial based analysis of the cost per case detected. Int J Cancer. 2005;117(6):981–7. doi: 10.1002/ijc.21220 . 16003735

[122] Levinson KL, Abuelo C, Chyung E, Salmeron J, Belinson SE, Sologuren CV, et al. The Peru cervical cancer prevention study (PERCAPS): community-based participatory research in Manchay, Peru. Int J Gynecol Cancer. 2013;23(1):141–7. doi: 10.1097/IGC.0b013e318275b007 . 23165314 PMC3529758

[123] Levinson KL, Abuelo C, Salmeron J, Chyung E, Zou J, Belinson SE, et al. The Peru Cervical Cancer Prevention Study (PERCAPS): the technology to make screening accessible. Gynecol Oncol. 2013;129(2):318–23. doi: 10.1016/j.ygyno.2013.01.026 . 23385153 PMC3632388

[124] Li M, Nyabigambo A, Navvuga P, Nuwamanya E, Nuwasiima A, Kaganda P, et al. Acceptability of cervical cancer screening using visual inspection among women attending a childhood immunization clinic in Uganda. Papillomavirus Res. 2017;4:17–21. doi: 10.1016/j.pvr.2017.06.004 . 29179864 PMC5883247

[125] Lidofsky A, Miller A, Jorgensen J, Tajik A, Tendeu K, Pius D, et al. Development and Implementation of a Culturally Appropriate Education Program to Increase Cervical Cancer Screening among Maasai Women in Rural Tanzania. Ann Glob Health. 2019;85(1):127. doi: 10.5334/aogh.2503 . 31673514 PMC6798900

[126] Lieber M, Afzal O, Shaia K, Mandelberger A, Du Preez C, Beddoe AM. Cervical Cancer Screening in HIV-Positive Farmers in South Africa: Mixed-Method Assessment. Ann Glob Health. 2019;85(1):15. doi: 10.5334/aogh.37 . 30993957 PMC6634387

[127] Liebermann EJ, VanDevanter N, Shirazian T, Frias Guzman N, Niles M, Healton C, et al. Barriers to Cervical Cancer Screening and Treatment in the Dominican Republic: Perspectives of Focus Group Participants in the Santo Domingo Area. J Transcult Nurs. 2020;31(2):121–7. doi: 10.1177/1043659619846247 . 31046602

[128] Lima ST, da Silva Nalin de Souza B, Franca AK, Salgado Filho N, Sichieri R. Dietary approach to hypertension based on low glycaemic index and principles of DASH (Dietary Approaches to Stop Hypertension): a randomised trial in a primary care service. Br J Nutr. 2013;110(8):1472–9. doi: 10.1017/S0007114513000718 . 23632203

[129] Lin G, Feng Z, Liu H, Li Y, Nie Y, Liang Y, et al. Mass screening for colorectal cancer in a population of two million older adults in Guangzhou, China. Sci Rep. 2019;9(1):10424. doi: 10.1038/s41598-019-46670-2 . 31320661 PMC6639356

[130] Linde DS, Andersen MS, Mwaiselage J, Manongi R, Kjaer SK, Rasch V. Effectiveness of One-Way Text Messaging on Attendance to Follow-Up Cervical Cancer Screening Among Human Papillomavirus-Positive Tanzanian Women (Connected2Care): Parallel-Group Randomized Controlled Trial. J Med Internet Res. 2020;22(4):e15863. doi: 10.2196/15863 . 32238335 PMC7163417

[131] Liyanage IK, Wickramasinghe K, Katulanda P, Jayawardena R, Karunathilake I, Friel S, et al. Integrating the development agenda with noncommunicable disease prevention in developing countries: A quasi-experimental study on inter-sectoral action and its impact on self-reported salt consumption-the INPARD study. Cardiovasc Diagn Ther. 2019;9(2):120–8. doi: 10.21037/cdt.2018.10.19 . 31143633 PMC6511677

[132] Lizbeth NCF, Cristina CAL, Jessica ZC, Eduardo TOL, de Dios ALJ, Leticia CSM. Individualized nutritional and pharmaceutical therapy consultation as a new strategy for the management of chronic diseases in actopan, Hidalgo. Int Res J Pharm. 2019;10(12):37–44.

[133] Luo JG, Han L, Chen LW, Gao Y, Ding XJ, Li Y, et al. Effect of Intensive Personalized “5As+5Rs” Intervention on Smoking Cessation in Hospitalized Acute Coronary Syndrome Patients Not Ready to Quit Immediately: A Randomized Controlled Trial. Nicotine Tob Res. 2018;20(5):596–605. doi: 10.1093/ntr/ntx126 . 28637193

[134] Lynch A, Sobuwa S, Castle N. Barriers to the implementation of prehospital thrombolysis in the treatment of ST-segment elevation myocardial infarction in South Africa: An exploratory inquiry. Afr J Emerg Med. 2020. 10.1016/j.afjem.2020.08.001 PMC7700957 33299757

[135] Ma GX, Yin L, Gao W, Tan Y, Liu R, Fang C, et al. Workplace-based breast cancer screening intervention in China. Cancer Epidemiol Biomarkers Prev. 2012;21(2):358–67. doi: 10.1158/1055-9965.EPI-11-0915 . 22155948

[136] Marino BCA, Ribeiro ALP, Alkmim MB, Antunes AP, Boersma E, Marcolino MS. Coordinated regional care of myocardial infarction in a rural area in Brazil: Minas Telecardio Project 2. Eur Heart J Qual Care Clin Outcomes. 2016;2(3):215–24. doi: 10.1093/ehjqcco/qcw020 . 29474619

[137] Martin CE, Tergas AI, Wysong M, Reinsel M, Estep D, Varallo J. Evaluation of a single-visit approach to cervical cancer screening and treatment in Guyana: feasibility, effectiveness and lessons learned. J Obstet Gynaecol Res. 2014;40(6):1707–16. doi: 10.1111/jog.12366 . 24888938

[138] Mash B, Powell D, du Plessis F, van Vuuren U, Michalowska M, Levitt N. Screening for diabetic retinopathy in primary care with a mobile fundal camera—evaluation of a South African pilot project. S Afr Med J. 2007;97(12):1284–8. . 18264611

[139] Matenge TG, Mash B. Barriers to accessing cervical cancer screening among HIV positive women in Kgatleng district, Botswana: A qualitative study. PLoS ONE [Electronic Resource]. 2018;13(10):e0205425. doi: 10.1371/journal.pone.0205425 . 30356248 PMC6200249

[140] Mathers LJ, Wigton TR, Leonhardt JG. Screening for cervical neoplasia in an unselected rural Guatemalan population using direct visual inspection after acetic acid application: a pilot study. J Low Genit Tract Dis. 2005;9(4):232–5. doi: 10.1097/01.lgt.0000179864.59951.91 . 16205195

[141] Mauad EC, Nicolau SM, Moreira LF, Haikel RL Jr., Longatto-Filho A, Baracat EC. Adherence to cervical and breast cancer programs is crucial to improving screening performance. Rural Remote Health. 2009;9(3):1241. . 19778158

[142] Maza M, Melendez M, Masch R, Alfaro K, Chacon A, Gonzalez E, et al. Acceptability of self-sampling and human papillomavirus testing among non-attenders of cervical cancer screening programs in El Salvador. Prev Med. 2018;114:149–55. doi: 10.1016/j.ypmed.2018.06.017 . 29958860

[143] Megevand E, Van Wyk W, Knight B, Bloch B. Can cervical cancer be prevented by a see, screen, and treat program? A pilot study. Am J Obstet Gynecol. 1996;174(3):923–8. doi: 10.1016/s0002-9378(96)70327-7 . 8633670

[144] Mehndiratta A, Mishra SC, Bhandarkar P, Chhatbar K, Cluzeau F, PrimaryCareDoctors T. Increasing identification of foot at risk of complications in patients with diabetes: a quality improvement project in an urban primary health centre in India. BMJ Open Qual. 2020;9(3):08. doi: 10.1136/bmjoq-2019-000893 . 32764027 PMC7412605

[145] Memon S, Ahsan S, Alvi R, Fawwad A, Basit A, Shera S, et al. Retinal Screening Acceptance, Laser Treatment Uptake and Follow-up Response in Diabetics Requiring Laser Therapy in an Urban Diabetes Care Centre. J Coll Physicians Surg Pak. 2015;25(10):743–6. doi: 10.2015/JCPSP.743746 . 26454391

[146] Mendoza Montano C, Fort M, deRamirez M, Cruz J, Ramirez-Zea M. Evaluation of a pilot hypertension management programme for Guatemalan adults. Health Promot Int. 2016;31(2):363–74. doi: 10.1093/heapro/dau117 . 25595280 PMC4863869

[147] Meng W, Bi XW, Bai XY, Pan HF, Cai SR, Zhao Q, et al. Barrier-focused intervention to increase colonoscopy attendance among nonadherent high-risk populations. World J Gastroenterol. 2009;15(31):3920–5. doi: 10.3748/wjg.15.3920 . 19701973 PMC2731255

[148] Mishra SI, Bastani R, Crespi CM, Chang LC, Luce PH, Baquet CR. Results of a randomized trial to increase mammogram usage among Samoan women. Cancer Epidemiol Biomarkers Prev. 2007;16(12):2594–604. doi: 10.1158/1055-9965.EPI-07-0148 . 18086763 PMC3612893

[149] Mittal S, Mandal R, Banerjee D, Das P, Ghosh I, Panda C, et al. HPV detection-based cervical cancer screening program in low-resource setting: lessons learnt from a community-based demonstration project in India. Cancer Causes Control. 2016;27(3):351–8. doi: 10.1007/s10552-015-0708-z . 26712612

[150] Modibbo F, Iregbu KC, Okuma J, Leeman A, Kasius A, de Koning M, et al. Randomized trial evaluating self-sampling for HPV DNA based tests for cervical cancer screening in Nigeria. Infect Agent Cancer [Electronic Resource]. 2017;12:11. doi: 10.1186/s13027-017-0123-z . 28184239 PMC5294803

[151] Moon TD, Silva-Matos C, Cordoso A, Baptista AJ, Sidat M, Vermund SH. Implementation of cervical cancer screening using visual inspection with acetic acid in rural Mozambique: successes and challenges using HIV care and treatment programme investments in Zambezia Province. J Int AIDS Soc. 2012;15(2):17406. doi: 10.7448/IAS.15.2.17406 . 22713260 PMC3499800

[152] Moreira J, Figueroa MM, Anselmi M, Prandi R, Montano CC, Bell D, et al. Long-Term Outcomes of a Cohort of Hypertensive Subjects in Rural Ecuador. Glob Heart. 2019;14(4):373–8. doi: 10.1016/j.gheart.2019.09.001 . 31727267

[153] Morrison J, Akter K, Jennings HM, Nahar T, Kuddus A, Shaha SK, et al. Participatory learning and action to address type 2 diabetes in rural Bangladesh: a qualitative process evaluation. BMC Endocr Disord. 2019;19(1):118. doi: 10.1186/s12902-019-0447-3 . 31684932 PMC6830002

[154] Muffih TP, Claudettea C, Manjuha F, DeGregoriob G, Mangaa S, Nulaha K, et al. Implementing a fee-for-service cervical cancer screening and treatment program in cameroon: Challenges and opportunities. J Glob Oncol. 2018;4(Supplement 2):73s. . 28536303 10.1634/theoncologist.2016-0383 PMC5507645

[155] Murthy GVS, Gilbert C, Shukla R, Bala V, Anirudh GG, Mukpalkar S, et al. Overview and project highlights of an initiative to integrate diabetic retinopathy screening and management in the public health system in India. Indian J Ophthalmol. 2020;68(Suppl 1):S12–S5. doi: 10.4103/ijo.IJO_1964_19 . 31937722 PMC7001184

[156] Nakalembe M, Makanga P, Kambugu A, Laker-Oketta M, Huchko MJ, Martin J. A public health approach to cervical cancer screening in Africa through community-based self-administered HPV testing and mobile treatment provision. Cancer Med. 2020;9(22):8701–12. doi: 10.1002/cam4.3468 . 32966684 PMC7666725

[157] Ndikom CM, Ofi BA, Omokhodion FO, Adedokun BO. Effects of educational intervention on women’s knowledge and uptake of cervical cancer screening in selected hospitals in Ibadan, Nigeria. Int J Health Promot Educ. 2017;55(5–6):259–71.

[158] Ng N, Nichter M, Padmawati RS, Prabandari YS, Muramoto M, Nichter M. Bringing smoking cessation to diabetes clinics in Indonesia. Chronic Illn. 2010;6(2):125–35. doi: 10.1177/1742395310364253 . 20444767

[159] Ngichabe SK, Muthaura PN, Murungi C, Muyoka J, Omenge E, Muchiri L. Cryotherapy Following Visual Inspection with Acetic Acid and Lugol’s Iodine (Via/Vili) in Khwisero, Western Kenya: Lesson from the Field Affecting Policy and Practice. East Afr Med J. 2013;90(10):316–23. . 26862641

[160] Nguyen HL, Ha DA, Goldberg RJ, Kiefe CI, Chiriboga G, Ly HN, et al. Culturally adaptive storytelling intervention versus didactic intervention to improve hypertension control in Vietnam- 12 month follow up results: A cluster randomized controlled feasibility trial. PLoS ONE. 2018;13(12). doi: 10.1371/journal.pone.0209912 . 30596749 PMC6312314

[161] Nguyen QN, Pham ST, Nguyen VL, Wall S, Weinehall L, Bonita R, et al. Implementing a hypertension management programme in a rural area: local approaches and experiences from Ba-Vi district, Vietnam. BMC Public Health. 2011;11:325. doi: 10.1186/1471-2458-11-325 . 21586119 PMC3112133

[162] Obioha KC, Dim CC, Ugwu EO, Chigbu CO, Enebe JT, Ozumba BC. Acceptability and outcome of cervical cytology in postnatal women and other nonpregnant women in Enugu, Nigeria: A cross-sectional study. J Clin Diagn Res. 2020;14(4):QC07–QC10.

[163] Ogilvie GS, Mitchell S, Sekikubo M, Biryabarema C, Byamugisha J, Jeronimo J, et al. Results of a community-based cervical cancer screening pilot project using human papillomavirus self-sampling in Kampala, Uganda. Int J Gynaecol Obstet. 2013;122(2):118–23. doi: 10.1016/j.ijgo.2013.03.019 . 23731506

[164] Ogola EN, Okello FO, Herr JL, Macgregor-Skinner E, Mulvaney A, Yonga G. Healthy Heart Africa-Kenya: A 12-Month Prospective Evaluation of Program Impact on Health Care Providers’ Knowledge and Treatment of Hypertension. Glob Heart. 2019;14(1):61–70. doi: 10.1016/j.gheart.2019.02.002 . 31036303

[165] Oranratanaphan S, Termrungruanglert W, Khemapech N. Acceptability of self-sampling HPV testing among Thai women for cervical cancer screening. Asian Pac J Cancer Prev. 2014;15(17):7437–41. doi: 10.7314/apjcp.2014.15.17.7437 . 25227855

[166] Ouedraogo Y, Furlane G, Fruhauf T, Badolo O, Bonkoungou M, Pleah T, et al. Expanding the Single-Visit Approach for Cervical Cancer Prevention: Successes and Lessons From Burkina Faso. Glob Health Sci Pract. 2018;6(2):288–98. doi: 10.9745/GHSP-D-17-00326 . 29959272 PMC6024624

[167] Page CM, Ibrahim S, Park LP, Huchko MJ. Systems-level barriers to treatment in a cervical cancer prevention program in Kenya: Several observational studies. PLoS ONE [Electronic Resource]. 2020;15(7):e0235264. doi: 10.1371/journal.pone.0235264 . 32658921 PMC7357749

[168] Palanuwong B. Alternative cervical cancer prevention in low-resource settings: Experiences of visual inspection by acetic acid with single-visit approach in the first five provinces of Thailand. Aust N Z J Obstet Gynaecol. 2007;47(1):54–60. doi: 10.1111/j.1479-828X.2006.00680.x . 17261102

[169] Pantano NP, Fregnani JH, Resende JC, Zeferino LC, Fonseca BO, Antoniazzi M, et al. Evaluation of human papillomavirus self-collection offered by community health workers at home visits among under-screened women in Brazil. J Med Screen. 2020:969141320941056. doi: 10.1177/0969141320941056 . 32703059

[170] Paolino M, Gago J, Le Pera A, Cinto O, Thouyaret L, Arrossi S. Adherence to triage among women with HPV-positive self-collection: A study in a middle-low income population in Argentina. Ecancermedicalscience. 2020;14(no pagination). 10.3332/ecancer.2020.1138 PMC7685770 33281930

[171] Parham GP, Mwanahamuntu MH, Kapambwe S, Muwonge R, Bateman AC, Blevins M, et al. Population-level scale-up of cervical cancer prevention services in a low-resource setting: development, implementation, and evaluation of the cervical cancer prevention program in Zambia. PLoS ONE [Electronic Resource]. 2015;10(4):e0122169. doi: 10.1371/journal.pone.0122169 . 25885821 PMC4401717

[172] Pastakia SD, Manyara SM, Vedanthan R, Kamano JH, Menya D, Andama B, et al. Impact of Bridging Income Generation with Group Integrated Care (BIGPIC) on Hypertension and Diabetes in Rural Western Kenya. J Gen Intern Med. 2017;32(5):540–8. doi: 10.1007/s11606-016-3918-5 . 27921256 PMC5400758

[173] Patel A, Praveen D, Maharani A, Oceandy D, Pilard Q, Kohli MPS, et al. Association of Multifaceted Mobile Technology-Enabled Primary Care Intervention with Cardiovascular Disease Risk Management in Rural Indonesia. JAMA Cardiol. 2019;4(10):978–86. doi: 10.1001/jamacardio.2019.2974 . 31461123 PMC6714032

[174] Patel S, Klein RM, Patel A, Klein RB, Aung M, Hoe W. Diabetic retinopathy screening and treatment in Myanmar: a pilot study. BMJ Open Ophthalmol. 2017;1(1):e000084. doi: 10.1136/bmjophth-2017-000084 . 29354714 PMC5721643

[175] Paul P, Winkler JL, Bartolini RM, Penny ME, Huong TT, Nga le T, et al. Screen-and-treat approach to cervical cancer prevention using visual inspection with acetic acid and cryotherapy: experiences, perceptions, and beliefs from demonstration projects in Peru, Uganda, and Vietnam. Oncologist. 2013;18(12):1278–84. doi: 10.1634/theoncologist.2013-0253 . 24217554 PMC3868422

[176] Paz-Soldan VA, Bayer AM, Nussbaum L, Cabrera L. Structural barriers to screening for and treatment of cervical cancer in Peru. Reprod Health Matters. 2012;20(40):49–58. doi: 10.1016/S0968-8080(12)40680-2 . 23245408 PMC3839786

[177] Pazoki R, Nabipour I, Seyednezami N, Imami SR. Effects of a community-based healthy heart program on increasing healthy women’s physical activity: a randomized controlled trial guided by Community-based Participatory Research (CBPR). BMC Public Health. 2007;7:216. doi: 10.1186/1471-2458-7-216 . 17716376 PMC2018720

[178] Pfaendler KS, Mwanahamuntu MH, Sahasrabuddhe VV, Mudenda V, Stringer JS, Parham GP. Management of cryotherapy-ineligible women in a “screen-and-treat” cervical cancer prevention program targeting HIV-infected women in Zambia: lessons from the field. Gynecol Oncol. 2008;110(3):402–7. doi: 10.1016/j.ygyno.2008.04.031 . 18556050 PMC2745977

[179] Phongsavan K, Phengsavanh A, Wahlstrom R, Marions L. Safety, feasibility, and acceptability of visual inspection with acetic acid and immediate treatment with cryotherapy in rural Laos. Int J Gynaecol Obstet. 2011;114(3):268–72. doi: 10.1016/j.ijgo.2011.03.009 . 21752376

[180] Pillay A, Trieu K, Santos JA, Sukhu A, Schultz J, Wate J, et al. Assessment of a Salt Reduction Intervention on Adult Population Salt Intake in Fiji. Nutrients. 2017;9(12):12. doi: 10.3390/nu9121350 . 29231897 PMC5748800

[181] Pimple S, Pednekar M, Mazumdar P, Goswami S, Shastri S. Predictors of quitting tobacco—results of a worksite tobacco cessation service program among factory workers in Mumbai, India. Asian Pac J Cancer Prev. 2012;13(2):533–8. doi: 10.7314/apjcp.2012.13.2.533 . 22524820

[182] Prabhakaran D, Jeemon P, Mohanan PP, Govindan U, Geevar Z, Chaturvedi V, et al. Management of acute coronary syndromes in secondary care settings in Kerala: impact of a quality improvement programme. Natl Med J India. 2008;21(3):107–11. . 19004139

[183] Praveen PA, Venkatesh P, Tandon N. Screening model for diabetic retinopathy among patients with type 1 diabetes attending a tertiary care setting in India. Indian J Ophthalmol. 2020;68(Suppl 1):S96–S9. doi: 10.4103/ijo.IJO_1830_19 . 31937741 PMC7001160

[184] Queiroz MS, de Carvalho JX, Bortoto SF, de Matos MR, das Gracas Dias Cavalcante C, Andrade EAS, et al. Diabetic retinopathy screening in urban primary care setting with a handheld smartphone-based retinal camera. Acta Diabetol. 2020;57(12):1493–9. doi: 10.1007/s00592-020-01585-7 . 32748176 PMC7398859

[185] Raj P, Singh S, Lewis MG, Shukla R, Murthy GVS, Gilbert C. Diabetic retinopathy screening uptake after health education with or without retinal imaging within the facility in two AYUSH hospitals in Hyderabad, India: A nonrandomized pilot study. Indian J Ophthalmol. 2020;68(Suppl 1):S56–S8. doi: 10.4103/ijo.IJO_2119_19 . 31937731 PMC7001171

[186] Rajalakshmi R, Shanthirani CS, Anandakumar A, Anjana RM, Murthy GVS, Gilbert C, et al. Assessment of diabetic retinopathy in type 1 diabetes in a diabetes care center in South India-Feasibility and awareness improvement study. Indian J Ophthalmol. 2020;68(Suppl 1):S92–S5. doi: 10.4103/ijo.IJO_1851_19 . 31937740 PMC7001175

[187] Ramachandran A, Kumar R, Nanditha A, Raghavan A, Snehalatha C, Krishnamoorthy S, et al. MDiabetes initiative using text messages to improve lifestyle and health-seeking behaviour in India. BMJ Innov. 2018;4(4):155–62.

[188] Ramagiri R, Kannuri NK, Lewis MG, Murthy GVS, Gilbert C. Evaluation of whether health education using video technology increases the uptake of screening for diabetic retinopathy among individuals with diabetes in a slum population in Hyderabad. Indian J Ophthalmol. 2020;68(Suppl 1):S37–S41. doi: 10.4103/ijo.IJO_2028_19 . 31937727 PMC7001174

[189] Ramogola-Masire D, de Klerk R, Monare B, Ratshaa B, Friedman HM, Zetola NM. Cervical cancer prevention in HIV-infected women using the “See and Treat” approach in Botswana. J Acquir Immune Defic Syndr. 2011;30. 10.1097/QAI.0b013e3182426227 PMC3884088 22134146

[190] Rashid RM, Ramli S, John J, Dahlui M. Cost effective analysis of recall methods for cervical cancer screening in Selangor—results from a prospective randomized controlled trial. Asian Pac J Cancer Prev. 2014;15(13):5143–7. doi: 10.7314/apjcp.2014.15.13.5143 . 25040965

[191] Rosenbaum AJ, Gage JC, Alfaro KM, Ditzian LR, Maza M, Scarinci IC, et al. Acceptability of self-collected versus provider-collected sampling for HPV DNA testing among women in rural El Salvador. Int J Gynaecol Obstet. 2014;126(2):156–60. doi: 10.1016/j.ijgo.2014.02.026 . 24880188

[192] Rosser JI, Njoroge B, Huchko MJ. Changing knowledge, attitudes, and behaviors regarding cervical cancer screening: The effects of an educational intervention in rural Kenya. Patient Educ Couns. 2015;98(7):884–9. doi: 10.1016/j.pec.2015.03.017 . 25858634 PMC4437717

[193] Sadeghi-Hokmabadi E, Farhoudi M, Taheraghdam A, Hashemilar M, Savadi-Osguei D, Rikhtegar R, et al. Intravenous recombinant tissue plasminogen activator for acute ischemic stroke: a feasibility and safety study. Int J Gen Med. 2016;9:361–7. doi: 10.2147/IJGM.S112430 . 27822079 PMC5087792

[194] Salamanca O, Geary A, Suarez N, Benavent S, Gonzalez M. Implementation of a diabetic retinopathy referral network, Peru. Bull World Health Organ. 2018;96(10):674–81. doi: 10.2471/BLT.18.212613 . 30455515 PMC6238996

[195] Salimzadeh H, Delavari A. Effectiveness of a theory-based intervention to increase colorectal cancer screening among Iranian. J Gastroenterol Hepatol. 2013;28:567–8. doi: 10.1007/s10865-013-9533-6 . 24027014

[196] Sanghvi H, Limpaphayom KK, Plotkin M, Charurat E, Kleine A, Lu E, et al. Cervical cancer screening using visual inspection with acetic acid: operational experiences from Ghana and Thailand. Reprod Health Matters. 2008;16(32):67–77. doi: 10.1016/S0968-8080(08)32401-X . 19027624

[197] Sankaranarayanan R, Rajkumar R, Esmy PO, Fayette JM, Shanthakumary S, Frappart L, et al. Effectiveness, safety and acceptability of ‘see and treat’ with cryotherapy by nurses in a cervical screening study in India. Br J Cancer. 2007;96(5):738–43. doi: 10.1038/sj.bjc.6603633 . 17311015 PMC2360066

[198] Saraf DS, Gupta SK, Pandav CS, Nongkinrih B, Kapoor SK, Pradhan SK, et al. Effectiveness of a School Based Intervention for Prevention of Non-communicable Diseases in Middle School Children of Rural North India: A Randomized Controlled Trial. Indian J Pediatr. 2015;82(4):354–62. doi: 10.1007/s12098-014-1562-9 . 25209052

[199] Sartorelli DS, Sciarra EC, Franco LJ, Cardoso MA. Beneficial effects of short-term nutritional counselling at the primary health-care level among Brazilian adults. Public Health Nutr. 2005;8(7):820–5. doi: 10.1079/phn2005737 . 16277797

[200] Scepanovic M, Jovanovic O, Keber D, Jovanovic I, Miljus D, Nikolic G, et al. Faecal occult blood screening for colorectal cancer in Serbia: a pilot study. Eur J Cancer Prev. 2017;26(3):195–200. doi: 10.1097/CEJ.0000000000000247 . 27082163

[201] Scott LaMontagne D, Barge S, Le NT, Mugisha E, Penny ME, Gandhi S, et al. Human papillomavirus vaccine delivery strategies. Bull World Health Organ 2011;89(11):821–30. doi: 10.2471/BLT.11.089862 22084528 PMC3209730

[202] Sharma SR, Sharma N. Hyperacute thrombolysis with recombinant tissue plasminogen activator of acute ischemic stroke: feasibility and effectivity from an Indian perspective. Ann Indian Acad Neurol. 2008;11(4):221–4. doi: 10.4103/0972-2327.44556 . 19893677 PMC2771988

[203] Shastri SS, Mittra I, Mishra GA, Gupta S, Dikshit R, Singh S, et al. Effect of VIA screening by primary health workers: randomized controlled study in Mumbai, India. J Natl Cancer Inst. 2014;106(3):dju009. doi: 10.1093/jnci/dju009 . 24563518 PMC3982783

[204] Shen J, Olwanda E, Kahn JG, Huchko MJ. Cost of HPV screening at community health campaigns (CHCs) and health clinics in rural Kenya. BMC Health Serv Res. 2018;18(1):378. doi: 10.1186/s12913-018-3195-6 . 29801496 PMC5970469

[205] Shiferaw N, Salvador-Davila G, Kassahun K, Brooks MI, Weldegebreal T, Tilahun Y, et al. The Single-Visit Approach as a Cervical Cancer Prevention Strategy Among Women With HIV in Ethiopia: Successes and Lessons Learned. Glob Health Sci Pract. 2016;4(1):87–98. doi: 10.9745/GHSP-D-15-00325 . 27016546 PMC4807751

[206] Singh S, Shukla AK, Sheikh A, Gupta G, More A. Effect of health education and screening location on compliance with diabetic retinopathy screening in a rural population in Maharashtra. Indian J Ophthalmol. 2020;68(Suppl 1):S47–S51. doi: 10.4103/ijo.IJO_1976_19 . 31937729 PMC7001165

[207] Singla S, Mathur S, Kriplani A, Agarwal N, Garg P, Bhatla N. Single visit approach for management of cervical intraepithelial neoplasia by visual inspection & loop electrosurgical excision procedure. Indian J Med Res. 2012;135(5):614–20. . 22771589 PMC3401690

[208] Snyman LC, Dreyer G, Botha MH, van der Merwe FH, Becker PJ. The Vaccine and Cervical Cancer Screen (VACCS) project: Linking cervical cancer screening to HPV vaccination in the South-West District of Tshwane, Gauteng, South Africa. S Afr Med J. 2015;105(2):115–20. doi: 10.7196/samj.8418 . 26242529

[209] Solla DJ, Paiva Filho Ide M, Delisle JE, Braga AA, Moura JB, Moraes X Jr., et al. Integrated regional networks for ST-segment-elevation myocardial infarction care in developing countries: the experience of Salvador, Bahia, Brazil. Circ Cardiovasc Qual Outcomes. 2013;6(1):9–17. doi: 10.1161/CIRCOUTCOMES.112.967505 . 23233748

[210] Souza GF, Figueira RM, Alkmim MB, Sousa LAP, Bonisson L, Ribeiro ALP, et al. Teleophthalmology Screening for Diabetic Retinopathy in Brazil: Applicability and Economic Assessment. Telemed J E Health. 2020;26(3):341–6. doi: 10.1089/tmj.2018.0241 . 30994411

[211] Sui Y, Luo J, Dong C, Zheng L, Zhao W, Zhang Y, et al. Implementation of regional Acute Stroke Care Map increases thrombolysis rates for acute ischaemic stroke in Chinese urban area in only 3 months. Stroke Vasc Neurol. 2020;24:24. doi: 10.1136/svn-2020-000332 . 32973114 PMC8005897

[212] Swaddiwudhipong W, Chaovakiratipong C, Nguntra P, Mahasakpan P, Tatip Y, Boonmak C. A mobile unit: an effective service for cervical cancer screening among rural Thai women. Int J Epidemiol. 1999;28(1):35–9. doi: 10.1093/ije/28.1.35 . 10195661

[213] Tamin NSI, Razalli KA, Sallahuddin SN, Chan HK, Hassan MRA. A 5-year evaluation of using stool-based test for opportunistic colorectal cancer screening in primary health institutions across Malaysia. Cancer Epidemiol. 2020;69(no pagination). doi: 10.1016/j.canep.2020.101829 . 32998070

[214] Tankumpuan T, Anuruang S, Jackson D, Hickman LD, DiGiacomo M, Davidson PM. Improved adherence in older patients with hypertension: An observational study of a community-based intervention. Int J Older People Nurs. 2019;14(3):e12248. doi: 10.1111/opn.12248 . 31173482

[215] Teguete I, Muwonge R, Traore CB, Dolo A, Bayo S, Sankaranarayanan R. Can visual cervical screening be sustained in routine health services? Experience from Mali, Africa. BJOG. 2012;119(2):220–6. doi: 10.1111/j.1471-0528.2011.03122.x . 21895956

[216] Tetra Dewi FS, Stenlund H, Marlinawati VU, Ohman A, Weinehall L. A community intervention for behaviour modification: an experience to control cardiovascular diseases in Yogyakarta, Indonesia. BMC Public Health. 2013;13:1043. doi: 10.1186/1471-2458-13-1043 . 24188684 PMC3840649

[217] Tian M, Yin X, Dunzhu D, Liu Z, Li C, Sun H, et al. A qualitative evaluation of a simplified cardiovascular management program in Tibet, China. Global Health. 2018;14(1):24. doi: 10.1186/s12992-018-0342-0 . 29490675 PMC5831713

[218] Todo MC, Bergamasco CM, Azevedo PS, Minicucci MF, Inoue RMT, Okoshi MP, et al. Impact of coronary intensive care unit in treatment of myocardial infarction. Rev Assoc Med Bras. 2017;63(3):242–7. doi: 10.1590/1806-9282.63.03.242 . 28489130

[219] Torres KL, Marino JM, Pires Rocha DA, de Mello MB, de Melo Farah HH, Reis RDS, et al. Self-sampling coupled to the detection of HPV 16 and 18 E6 protein: A promising option for detection of cervical malignancies in remote areas. PLoS ONE [Electronic Resource]. 2018;13(7):e0201262. doi: 10.1371/journal.pone.0201262 . 30036381 PMC6056043

[220] Tran VD, Jancey J, Lee A, James A, Howat P, Thi Phuong Mai L. Physical activity and nutrition program for adults with metabolic syndrome: Process evaluation. Eval Program Plann. 2017;61:128–33. doi: 10.1016/j.evalprogplan.2016.12.012 . 28063345

[221] Tsvetanova Dimova R, Dimitrova Dimitrova D, Angelova Levterova B, Stoyanov Dimov R, Atanasova Semerdjieva M, Frantova Tarnovska M, et al. Feasibility of immunochemical faecal occult blood testing for colorectal cancer screening in Bulgaria. J BUON. 2015;20(2):413–20. . 26011330

[222] Tutino GE, Yang WY, Li X, Li WH, Zhang YY, Guo XH, et al. A multicentre demonstration project to evaluate the effectiveness and acceptability of the web-based Joint Asia Diabetes Evaluation (JADE) programme with or without nurse support in Chinese patients with Type 2 diabetes. Diabet Med. 2017;34(3):440–50. doi: 10.1111/dme.13164 . 27278933 PMC5324581

[223] Uivarosan D, Bungau S, Tit DM, Moisa C, Fratila O, Rus M, et al. Financial Burden of Stroke Reflected in a Pilot Center for the Implementation of Thrombolysis. Medicina. 2020;56(2):28. doi: 10.3390/medicina56020054 . 32013001 PMC7074434

[224] Vinitha R, Nanditha A, Snehalatha C, Satheesh K, Susairaj P, Raghavan A, et al. Effectiveness of mobile phone text messaging in improving glycaemic control among persons with newly detected type 2 diabetes. Diabetes Res Clin Pract. 2019;158(no pagination). doi: 10.1016/j.diabres.2019.107919 . 31711858

[225] Walley JD, Khan N, Khan MA, Ali S, King R, Khan SE, et al. Delivering integrated hypertension care at private health facilities in urban pakistan: A process evaluation. BJGP Open. 2018;2(4):1–12. doi: 10.3399/bjgpopen18X101613 . 30723799 PMC6348318

[226] Wan LH, Zhang XP, You LM, Ruan HF, Chen SX. The Efficacy of a Comprehensive Reminder System to Improve Health Behaviors and Blood Pressure Control in Hypertensive Ischemic Stroke Patients: A Randomized Controlled Trial. J Cardiovasc Nurs. 2018;33(6):509–17. doi: 10.1097/JCN.0000000000000496 . 29901484

[227] Wang B, Wang Y, Ye T, Xiao G, Chang H, Wen H, et al. [Integrated regional network construction for ST-segment elevation myocardial infarction care]. Chung-Hua Hsin Hsueh Kuan Ping Tsa Chih [Chinese Journal of Cardiology]. 2014;42(8):650–4. . 25388337

[228] Wang X, Liu D, Du M, Hao R, Zheng H, Yan C. The role of text messaging intervention in Inner Mongolia among patients with type 2 diabetes mellitus: a randomized controlled trial. BMC Med Inform Decis Mak. 2020;20(1):90. doi: 10.1186/s12911-020-01129-7 . 32410608 PMC7222448

[229] Wang YH, Ye KY, Wang SY, Wu F, Yan QH, Cheng MN, et al. Real world study of influenza vaccination intervention among key population of chronic disease management in Shanghai community. Chung-Hua Yu Fang i Hsueh Tsa Chih [Chinese Journal of Prev Med]. 2020;54(4):425–9. doi: 10.3760/cma.j.cn112150-20191031-00828 . 32268652

[230] Webb EM, Rheeder P. A cluster-randomized trial to estimate the effect of mobile screening and treatment feedback on HbA1c and diabetes-related complications in Tshwane primary health care clinics, South Africa. Prim Care Diabetes. 2017;11(6):546–54. doi: 10.1016/j.pcd.2017.05.010 . 28690088

[231] Wei X, Walley JD, Zhang Z, Zou G, Gong W, Deng S, et al. Implementation of a comprehensive intervention for patients at high risk of cardiovascular disease in rural China: A pragmatic cluster randomized controlled trial. PLoS ONE [Electronic Resource]. 2017;12(8):e0183169. doi: 10.1371/journal.pone.0183169 . 28813512 PMC5559073

[232] Wu Y, Li S, Patel A, Li X, Du X, Wu T, et al. Effect of a Quality of Care Improvement Initiative in Patients with Acute Coronary Syndrome in Resource-Constrained Hospitals in China: A Randomized Clinical Trial. JAMA Cardiol. 2019;4(5):418–27. doi: 10.1001/jamacardio.2019.0897 . 30994898 PMC6537808

[233] Wu Y, Liang Y, Zhou Q, Liu H, Lin G, Cai W, et al. Effectiveness of a short message service intervention to motivate people with positive results in preliminary colorectal cancer screening to undergo colonoscopy: A randomized controlled trial. Cancer. 2019;125(13):2252–61. doi: 10.1002/cncr.32043 . 30825395

[234] Xiao M, Lei X, Zhang F, Sun Z, Harris VC, Tang X, et al. Home Blood Pressure Monitoring by a Mobile-Based Model in Chongqing, China: A Feasibility Study. Int J Environ Res Public Health [Electronic Resource]. 2019;16(18):10. doi: 10.3390/ijerph16183325 . 31509950 PMC6765873

[235] Yazdanpanah B, Safari M, Yazdanpanah S, Angha P, Karami M, Emadi M, et al. The effect of participatory community-based diabetes cares on the control of diabetes and its risk factors in western suburb of Yasouj, Iran. Health Educ Res. 2012;27(5):794–803. doi: 10.1093/her/cys079 . 22907534

[236] Yeates KE, Sleeth J, Hopman W, Ginsburg O, Heus K, Andrews L, et al. Evaluation of a Smartphone-Based Training Strategy Among Health Care Workers Screening for Cervical Cancer in Northern Tanzania: The Kilimanjaro Method. J Glob Oncol. 2016;2(6):356–64. doi: 10.1200/JGO.2015.001768 . 28717721 PMC5493243

[237] You J, Wang S, Li J, Luo Y. Usefulness of a nurse-led program of care for management of patients with chronic heart failure. Med Sci Monit. 2020;26(no pagination). doi: 10.12659/MSM.920469 . 32068197 PMC7047924

[238] Yu R, Yan LL, Wang H, Ke L, Yang Z, Gong E, et al. Effectiveness of a community-based individualized lifestyle intervention among older adults with diabetes and hypertension, Tianjin, China, 2008–2009. Prev Chronic Dis. 2014;11:E84. doi: 10.5888/pcd11.120333 . 24831288 PMC4023677

[239] Yu Y, Lv Y, Yao B, Duan L, Zhang X, Xie L, et al. A novel prescription pedometer-assisted walking intervention and weight management for Chinese occupational population. PLoS ONE [Electronic Resource]. 2018;13(1):e0190848. doi: 10.1371/journal.pone.0190848 . 29324808 PMC5764333

[240] Zhang Q, Zhang RY, Qiu JP, Zhang JF, Wang XL, Jiang L, et al. Prospective multicenter randomized trial comparing physician versus patient transfer for primary percutaneous coronary intervention in acute ST-segment elevation myocardial infarction. Chin Med J (Engl). 2008;121(6):485–91. . 18364130

[241] Zhang X, Zhao G, Bi H, Zhou M, Wang X, Juan J. Exploring an Appropriate Method of Cervical Cancer Screening in Rural China. Asia Pac J Public Health. 2019;31(7):652–8. doi: 10.1177/1010539519876411 . 31578073

[242] Zhao Y, Liao Q, Mi X, Li MZ, Zhao C, Cui SH, et al. [Survey of the acceptance status of HPV self-sampling screening in female population for cervical cancer]. Chung-Hua Fu Chan Ko Tsa Chih [Chinese Journal of Obstetrics & Gynecology]. 2019;54(5):312–7. doi: 10.3760/cma.j.issn.0529-567x.2019.05.005 . 31154712

[243] Zheng X, Spatz ES, Bai X, Huo X, Ding Q, Horak P, et al. Effect of Text Messaging on Risk Factor Management in Patients With Coronary Heart Disease: The CHAT Randomized Clinical Trial. Circ Cardiovasc Qual Outcomes. 2019;12(4):e005616. doi: 10.1161/CIRCOUTCOMES.119.005616 . 30998400

[244] Zhong X, Potemans B, Zhang L, Oldenburg B. Getting a Grip on NCDs in China: an Evaluation of the Implementation of the Dutch-China Cardiovascular Prevention Program. Int J Behav Med. 2015;22(3):393–403. doi: 10.1007/s12529-014-9453-z . 25471465 PMC4449379

[245] Zhong X, Wang Z, Fisher EB, Tanasugarn C. Peer Support for Diabetes Management in Primary Care and Community Settings in Anhui Province, China. Ann Fam Med. 2015;13(Suppl 1):S50–8. doi: 10.1370/afm.1799 . 26304972 PMC4648136

[246] Forouzanfar MH, Afshin A, Alexander LT, Anderson HR, Bhutta ZA, Biryukov S, et al. Global, regional, and national comparative risk assessment of 79 behavioural, environmental and occupational, and metabolic risks or clusters of risks, 1990–2015: a systematic analysis for the Global Burden of Disease Study 2015. Lancet. 2016;388(10053):1659–1724. doi: 10.1016/S0140-6736(16)31679-8 27733284 PMC5388856

[247] Collaboration NCDRF. Worldwide trends in hypertension prevalence and progress in treatment and control from 1990 to 2019: a pooled analysis of 1201 population-representative studies with 104 million participants. Lancet. 2021. Epub 2021/08/28. doi: 10.1016/S0140-6736(21)01330-1 . 34450083 PMC8446938

[248] Tomlinson M, Rotheram-Borus MJ, Swartz L, Tsai AC. Scaling up mHealth: where is the evidence? PLoS Med. 2013;10(2):e1001382. Epub 2013/02/21. doi: 10.1371/journal.pmed.1001382 ; PubMed Central PMCID: PMC3570540. 23424286 PMC3570540

[249] Vedanthan R, Bernabe-Ortiz A, Herasme OI, Joshi R, Lopez-Jaramillo P, Thrift AG, et al. Innovative Approaches to Hypertension Control in Low- and Middle-Income Countries. Cardiol Clin. 2017;35(1):99–115. Epub 2016/11/26. doi: 10.1016/j.ccl.2016.08.010 ; PubMed Central PMCID: PMC5131527. 27886793 PMC5131527

[250] Kruk ME, Pate M, Mullan Z. High quality health systems—time for a revolution: Report of Lancet Glob Health Commission on High Quality Health Systems in the SDG Era Lancet. Global Health. 2018. 10.1016/S2214-109X(17)30101-8 28302563

[251] Sheikh K, Abimbola S, Organization WH. Learning health systems: pathways to progress: flagship report of the Alliance for Health Policy and Systems Research. 2021.

[252] Curran GM, Bauer M, Mittman B, Pyne JM, Stetler C. Effectiveness-implementation hybrid designs: combining elements of clinical effectiveness and implementation research to enhance public health impact. Med Care. 2012;50(3):217–26. Epub 2012/02/09. doi: 10.1097/MLR.0b013e3182408812 ; PubMed Central PMCID: PMC3731143. 22310560 PMC3731143

[253] Kirk MA, Kelley C, Yankey N, Birken SA, Abadie B, Damschroder L. A systematic review of the use of the consolidated framework for implementation research. Implement Sc. 2015;11(1):1–13. 10.1186/s13012-016-0437-z PMC4869309 27189233

[254] Jailobaeva K, Falconer J, Loffreda G, Arakelyan S, Witter S, Ager A. An analysis of policy and funding priorities of global actors regarding noncommunicable disease in low- and middle-income countries. Global Health. 2021;17(1):68. Epub 2021/07/01. doi: 10.1186/s12992-021-00713-4 ; PubMed Central PMCID: PMC8240078. 34187499 PMC8240078

[255] Beyeler N, Fewer S, Yotebieng M, Yamey G. Improving resource mobilisation for global health R&D: a role for coordination platforms? BMJ Glob Health. 2019;4(1):e001209. Epub 2019/03/23. doi: 10.1136/bmjgh-2018-001209 ; PubMed Central PMCID: PMC6407558. 30899563 PMC6407558

[256] Kruk ME, Yamey G, Angell SY, Beith A, Cotlear D, Guanais F, et al. Transforming Global Health by Improving the Science of Scale-Up. PLoS Biol. 2016;14(3):e1002360. Epub 2016/03/05. doi: 10.1371/journal.pbio.1002360 ; PubMed Central PMCID: PMC4775018. 26934704 PMC4775018

[257] Patel S, Craigen G, Pinto da Costa M, Inkster B. Opportunities and Challenges for Digital Social Prescribing in Mental Health: Questionnaire Study. J Med Internet Res. 2021;23(3):e17438. Epub 2021/03/10. doi: 10.2196/17438 ; PubMed Central PMCID: PMC7988390. 33687338 PMC7988390

[258] Daniel M, Maulik PK, Kallakuri S, Kaur A, Devarapalli S, Mukherjee A, et al. An integrated community and primary healthcare worker intervention to reduce stigma and improve management of common mental disorders in rural India: protocol for the SMART Mental Health programme. Trials. 2021;22(1):179. Epub 2021/03/04. doi: 10.1186/s13063-021-05136-5 ; PubMed Central PMCID: PMC7923507. 33653406 PMC7923507

[259] Asubiaro T. Sub-Saharan Africa’s Biomedical Journal Coverage in Scholarly Databases: A comparison of Web of Science, Scopus, EMBASE, PubMed, African Index Medicus and African Journals Online. 2021. 10.5195/jmla.2023.1448 PMC10361549 37483369

[260] Kieling C, Herrman H, Patel V, Mari JdJ. Indexation of psychiatric journals from low- and middle-income countries: a survey and a case study. World Psychiatry. 2009;8(1):40. doi: 10.1002/j.2051-5545.2009.tb00209.x 19293959 PMC2809367

[261] Pilkington K, Boshnakova A, Clarke M, Richardson J. “No language restrictions” in database searches: what does this really mean? J Altern Complement Med. 2005;11(1):205–207. doi: 10.1089/acm.2005.11.205 15750383

[262] Shenderovich Y, Eisner M, Mikton C, Gardner F, Liu J, Murray J. Methods for conducting systematic reviews of risk factors in low-and middle-income countries. BMC Med Res Methodol. 2016;16(1):1–8. doi: 10.1186/s12874-016-0134-2 26979282 PMC4791911

[263] Sheriff RJS, Adams CE, Tharyan P, Jayaram M, Duley L. Randomised trials relevant to mental health conducted in low and middle-income countries: a survey. BMC Psychiatry. 2008;8(1):1–9. doi: 10.1186/1471-244X-8-69 18702809 PMC2527605

[264] Beard P, Greenall J, Hoffman C, Nettleton S, Popescu I, Ste-Marie M. Incident Analysis Collaborating Parties. Canadian Incident Analysis Framework. Edmonton, AB: Canadian Patient Safety Institute; 2012.

