# Supplementary material for: Implementation research on noncommunicable disease prevention and control interventions in low- and middle-income countries: A systematic review
Source: PLoS Med. 2022 Jul 25;19(7):e1004055. doi: 10.1371/journal.pmed.1004055 (PMC9359585; doi:10.1371/journal.pmed.1004055)
Supplement: S1 Appendix — Table A in S1 Appendix. Interventions provided within health systems. Table B in S1 Appendix. Sample of the search strategy used in the MEDLINE database. Table C in S1 Appendix. List of low- and middle-income countries. Table D in S1 Appendix. Data extraction tool. Table E in S1 Appendix. Distribution of studies by countries where they were implemented. Fig A in S1 Appendix. Variation of conditions evaluated by income group. Fig B in S1 Appendix. Priority NCD interventions (n = 265) identified in 222 studies included in the review. Fig C in S1 Appendix. Distribution of included studies by NCD. Fig D in S1 Appendix. Distribution of intervention type by income group. Fig E in S1 Appendix. Distributions by research designs. Fig F in S1 Appendix. Distributions by standalone implementation studies vs. embedded or hybrid effectiveness-implementation studies. Fig G in S1 Appendix. Distributions by pilot vs. scale-up project. Fig H in S1 Appendix. Variation by level of health system. Fig I in S1 Appendix. Studies that reported funding (vs. those that did not) by NCD conditions. Fig J in S1 Appendix. Distributions by funding type. Fig K in S1 Appendix. Distribution of funding sources by NCDs and their risk factors. Fig L in S1 Appendix. Types of reported funding sources (N = 222 included studies). (DOCX) [file pmed.1004055.s002.docx]

**S 1 Appendix. Appendix tables and figures**

Hategeka C, Adu P, Desloge A, Marten R, Shao R, Tian M, Wei T, Kruk ME. Implementation Research on Non-Communicable Disease Prevention and Control Interventions in Low- and Middle-Income Countries: A Systematic Review

**Table A in S1 Appendix. Interventions provided within health systems [1]**

| **Conditions**  (i.e., NDCs or Risk factors) | **Interventions** | **Ranking of interventions*** | **Category of interventions†** |
| --- | --- | --- | --- |
| Tobacco use | - Implement effective mass media campaigns that educate the public about the harms of smoking/tobacco use and second hand smoke | Best buy | Primary prevention |
|  | - Provide cessation for tobacco cessation to all those who want to quit | Other | Secondary prevention |
| Harmful use of alcohol | - Provide brief psychosocial intervention for persons with hazardous and harmful alcohol use | Good buy | Secondary prevention |
|  | - Provide prevention, treatment and care for alcohol use disorders and comorbid conditions in health and social services | Other | Primary prevention and treatment |
| Unhealthy diet | - Reduce salt intake through the establishment of a supportive environment in public institutions such as hospitals, schools, workplaces and nursing homes, to enable lower sodium options to be provided - Reduce salt intake through a behaviour change communication and mass media campaigns | Best buy | Primary prevention |
|  | - Promote and support exclusive breastfeeding for the first 6 months of life, including promotion of breastfeeding - Implement nutrition education and counselling in different settings (for example, in preschools, schools, workplaces and hospitals) to increase the intake of fruits and vegetables - Implement mass media campaign on healthy diets, including social marketing to reduce the intake of total fat, saturated fats, sugars and salt, and promote the intake of fruits and vegetables | Other | Primary prevention |
| Physical inactivity | - Implement community wide public education and awareness campaign for physical activity which includes a mass media campaign combined with other community based education, motivational and environmental programs aimed at supporting behavioural change of physical activity levels | Best buy | Primary prevention |
|  | - Provide physical activity counselling and referral as part of routine primary health care services through the use of a brief intervention | Good buy | Secondary prevention? |
| Cardiovascular disease | - Drug therapy and counselling to individuals who have had a heart attack or stroke and to persons with high risk (≥ 30%) of a fatal and non-fatal cardiovascular event in the next 10 years | Best buy | Primary and secondary prevention |
|  | - Treatment of new cases of acute myocardial infarction with either: acetylsalicylic acid, or acetylsalicylic acid and clopidogrel, or thrombolysis, or primary percutaneous coronary interventions - Treatment of acute ischemic stroke with intravenous thrombolytic therapy - Primary prevention of rheumatic fever and rheumatic heart diseases by increasing appropriate treatment of streptococcal pharyngitis at the primary care level - Secondary prevention of rheumatic fever and rheumatic heart disease by developing a register of patients who receive regular prophylactic penicillin | Good buy | Primary and secondary prevention |
|  | - Treatment of congestive cardiac failure with angiotensin-converting-enzyme inhibitor, beta-blocker and diuretic - Cardiac rehabilitation post myocardial infarction - Anticoagulation for medium-and high-risk non-valvular atrial fibrillation and for mitral stenosis with atrial fibrillation - Low-dose acetylsalicylic acid for ischemic stroke - Care of acute stroke and rehabilitation in stroke units | Other | Secondary and tertiary prevention |
|  | - Management of hypertension |  | Secondary prevention |
| Diabetes | - Preventive foot care for people with diabetes (including educational programmes, access to appropriate footwear, multidisciplinary clinics) - Diabetic retinopathy screening for all diabetes patients and laser photocoagulation for prevention of blindness 1 - Effective glycaemic control for people with diabetes, along with standard home glucose monitoring for people treated with insulin to reduce diabetes complications | Good buy | Secondary prevention |
|  | - Lifestyle interventions for preventing type 2 diabetes - Influenza vaccination for patients with diabetes - Preconception care among women of reproductive age who have diabetes including patient education and intensive glucose management - Screening of people with diabetes for proteinuria and treatment with angiotensin converting enzyme inhibitor for the prevention and delay of renal disease | Other | Primary and secondary prevention |
| Cancer | - Vaccination against human papillomavirus (2 doses) of 9–13 year old girls - Prevention of cervical cancer by screening women aged 30–49 years, either through: - Visual inspection with acetic acid linked with timely treatment of pre-cancerous lesions - Pap smear (cervical cytology) every 3–5 years linked with timely treatment of pre-cancerous lesions - Human papillomavirus test every 5 years linked with timely treatment of pre-cancerous lesions | Best buy | Primary and secondary prevention |
|  | - Screening with mammography (once every 2 years for women aged 50-69 years) linked with timely diagnosis and treatment of breast cancer - Treatment of colorectal cancer stages I and II with surgery +/- chemotherapy and radiotherapy - Basic palliative care for cancer: home-based and hospital care with multi-disciplinary team and access to opiates and essential supportive medicines | Good buy | Secondary and tertiary prevention |
|  | - Prevention of liver cancer through hepatitis B immunization - Oral cancer screening in high-risk groups (for example, tobacco users, betel-nut chewers) linked with timely treatment - Population-based colorectal cancer screening, including through a faecal occult blood test, as appropriate, at age >50 years, linked with timely treatment | Other | Primary and secondary prevention |
| Chronic respiratory disease | - Symptom relief for patients with asthma with inhaled salbutamol - Symptom relief for patients with chronic obstructive pulmonary disease with inhaled salbutamol - Treatment of asthma using low dose inhaled beclometasone and short acting beta agonist | Good buy | Tertiary prevention |
|  | - Influenza vaccination for patients with chronic obstructive pulmonary disease | Other | Primary prevention |

* ‘Best buys’: Effective interventions with cost effectiveness analysis ≤ I$ 100 per DALY averted in LMICs. Good buys: Effective interventions with cost effectiveness analysis >I$ 100 per DALY averted in LMICs. ‘Other recommended interventions from WHO guidance (cost effective analysis not available).

† NCD interventions evaluated in studies included in this review were further classified into three categories:

- Prevention category includes interventions whose focus was primary prevention
- Screening category includes interventions that focused on screening
- Treatment category includes therapeutic interventions

**Additional notes**:

- Include studies that look at factors affecting implementation of interventions above
- Include studies that include multiple interventions including at least one intervention above

**Table B in S1 Appendix. Sample of the search strategy used in the MEDLINE database**

| 1 (implementation adj2 (research$ or strateg$ or approach$ or science$ or evaluation$ or process$)).ti,ab. (20234) |
| --- |
| 2 (diffusion adj2 innovation$).ti,ab. (1010) |
| 3 (process$ adj2 evaluation$).ti,ab. (9382) |
| 4 (dissemination adj2 science).ti,ab. (82) |
| 5 (deliver$ adj2 (healthcare or health service$ or health intervention$ or health care or medical care or medical service$ or science)).ti,ab. (25430) |
| 6 (acceptab$ or adopt$ or uptak$ or feasib$ or appropriate$ or sustainab$ or fidelity or penetrat$ or implementation).ti,ab. (2093646) |
| 7 1 or 2 or 3 or 4 or 5 or 6 (2117415) |
| 8 Noncommunicable Diseases/ (1462) |
| 9 cardiovascular diseases/ or heart diseases/ or vascular diseases/ or cerebrovascular diseases/ (300166) |
| 10 exp Myocardial Ischemia/ (436095) |
| 11 Heart Failure/ (121688) |
| 12 exp brain ischemia/ or exp stroke/ (188862) |
| 13 exp Diabetes Mellitus, Type 2/ (137161) |
| 14 lung diseases, obstructive/ or exp pulmonary disease, chronic obstructive/ (74566) |
| 15 exp Asthma/ (129881) |
| 16 ((cardiovascular or cardio-vascular) adj3 (event* or outcome* or risk*)).ti,ab. (154641) |
| 17 ((coronary or heart or myocard*) adj3 disease*).ti,ab. (277152) |
| 18 ((coronary or heart or myocard*) adj3 (event* or outcome* or risk*)).ti,ab. (75786) |
| 19 ((ischaemic or ischemic or ischaemia or ischemia) adj3 disease*).ti,ab. (47100) |
| 20 ((ischaemic or ischemic or ischaemia or ischemia) adj3 (event* or outcome* or risk*)).ti,ab. (27086) |
| 21 ((cerebrovascular or vascular) adj3 disease*).ti,ab. (72711) |
| 22 high blood pressure.mp. or exp Hypertension/ (264875) |
| 23 ((cerebrovascular or vascular) adj3 (event* or outcome* or risk*)).ti,ab. (30259) |
| 24 (stroke or heart failure or myocardial infarct*).ti,ab. (564558) |
| 25 diabet*.ti,ab. (653663) |
| 26 ((type 2 or type ii or noninsulin dependent or non insulin dependent or adult onset or maturity onset or obes*) adj2 diabet*).ti,ab. (183796) |
| 27 (niddm or t2dm or tiidm).ti,ab. (29438) |
| 28 chronic obstructive pulmonary disease.ti,ab. (49828) |
| 29 (neoplas* or cancer* or carcinoma* or tumor* or tumour* or malignan* or leukaemia or leukemia).ti,ab. (3556629) |
| 30 (chronic adj2 (lung or pulmonary or respiratory)).ti,ab. (83262) |
| 31 8 or 9 or 10 or 11 or 12 or 13 or 14 or 15 or 16 or 17 or 18 or 19 or 20 or 21 or 22 or 23 or 24 or 25 or 26 or 27 or 28 or 29 or 30 (5752349) |
| 32 (influenza adj2 (vaccin* or immuni?ation or immuni?e) adj3 (diabet$ or (chronic adj2 (lung or pulmonary or respiratory)))).ti,ab. (55) |
| 33 (screen* adj3 (colorectal cancer or oral cancer)).ti,ab. (8953) |
| 34 Angiotensin-Converting Enzyme Inhibitors/ (33152) |
| 35 (exclusive breastfeeding or breastfeeding).ti,ab. (27260) |
| 36 (clopidogrel or thrombol$ or primary percutaneous coronary).ti,ab. (55751) |
| 37 ((primary prevention or secondary prevention) adj2 (rheumatic fever or rheumatic heart disease$)).ti,ab. (63) |
| 38 ((treatment or chemotherap$ or radiotherap$) adj3 colorectal cancer).ti,ab. (8160) |
| 39 (Cardiac rehab$ adj3 myocardial infarction).ti,ab. (166) |
| 40 ((salbutamol or low dose inhaled beclomethasone or short acting beta agonist) adj3 (chronic obstructive pulmonary disease or asthma)).ti,ab. (193) |
| 41 ((screen$ or mammography) adj3 (cancer adj2 breast)).ti,ab. (10189) |
| 42 (glycaemic control adj3 diabet$).ti,ab. (1011) |
| 43 ((screen$ or laser photocoagulat$) adj3 (diabetic retinopathy or diabet$)).ti,ab. (6534) |
| 44 (advertising as topic/ or Mass Media/) and ("Tobacco Use"/ or exp "Tobacco Use Cessation"/ or drinking behavior/ or exp alcohol drinking/ or exp Diet/ or food/ or exp dietary fats/ or fast foods/ or fruit/ or vegetables/ or Sodium Chloride, Dietary/ or Beverages/ or exp Exercise/ or Motor Activity/ or health behavior/ or risk reduction behavior/) (2349) |
| 45 (Patient Education as Topic/ or Counseling/ or Patient Compliance/ or Motivational Interviewing/) and ("Tobacco Use"/ or drinking behavior/ or exp alcohol drinking/ or exp dietary fats/ or Sodium Chloride/) (2525) |
| 46 ((food? or diet* or vegetable? or fruit? or sugar* or fat or fats or sucrose or candy or sweet* or snack* or fastfood? or junkfood?) and (marketing or adverti?ing or counsel* or educat* or campaign$)).ti,ab. (52931) |
| 47 (Health Education/ or Health Knowledge, Attitudes, Practice/) and (Life Style/ or Food Habits/ or Diet/ or Health Behavior/ or Obesity/ or Motor Activity/) (19839) |
| 48 ((Tobacco or smok* or alcohol$) adj3 (cessation$ or quit$ or stop$)).ti,ab. (38344) |
| 49 best buys.mp. (57) |
| 50 (aspirin or acetylsalicylic acid).ti,ab. (56640) |
| 51 Primary Prevention/ and Aspirin/ (701) |
| 52 ((hepatitis b or hep b) adj3 (vaccin* or immuni?ation or immuni?e)).ti,ab. (10255) |
| 53 Hepatitis B Vaccines/ (9378) |
| 54 ((cervical or pap) adj3 screen*).ti,ab. (12688) |
| 55 Mass Screening/ and (Uterine Cervical Neoplasms/ or Cervical Intraepithelial Neoplasia/) (7518) |
| 56 Papanicolaou Test/ (6725) |
| 57 Hydroxymethylglutaryl-CoA Reductase Inhibitors/ or Simvastatin/ or Aspirin/ or Metformin/ or Adrenergic beta-Antagonists/ or Propranolol/ or *Aspirin/ (156946) |
| 58 (Patient Education as Topic/ or Counseling/ or Patient Compliance/ or Motivational Interviewing/) and (Neoplasms/ or Obesity/ or Diabetes Mellitus, Type 2/ or Smoking/ or Risk Factors/ or Cardiovascular Diseases/ or Hypertension/) (30326) |
| 59 Drug therapy, combination.mp. and (Diabetes Mellitus, Type 2/ or Cardiovascular Diseases/ or Hypertension/) (12626) |
| 60 Health Promotion/ and (Neoplasms/ or Obesity/ or Diabetes Mellitus, Type 2/ or Smoking/ or Risk Factors/ or Cardiovascular Diseases/ or Hypertension/) (14796) |
| 61 (salt adj3 (intake or reduc* or lower*)).ti,ab. (9460) |
| 62 (foot care adj3 diabet$).ti,ab. (427) |
| 63 (psychosocial intervention adj3 alcohol$).ti,ab. (12) |
| 64 ((prevent$ or treat$ or care or manag$) adj3 alcohol$).ti,ab. (20944) |
| 65 (Anticoagulat$ adj3 (non?valvular atrial fibrillation or mitral stenosis)).ti,ab. (75) |
| 66 32 or 33 or 34 or 35 or 36 or 37 or 38 or 39 or 40 or 41 or 42 or 43 or 44 or 45 or 46 or 47 or 48 or 49 or 50 or 51 or 52 or 53 or 54 or 55 or 56 or 57 or 58 or 59 or 60 or 61 or 62 or 63 or 64 or 65 (526654) |
| 67 ((developing or less* developed or under developed or underdeveloped or middle income or low* income or underserved or under served or deprived or poor*) adj (countr* or nation? or state? or population? or world)).ti,ab. (107805) |
| 68 ((developing or less* developed or under developed or underdeveloped or middle income or low* income) adj (economy or economies)).ti,ab. (624) |
| 69 (low* adj (gdp or gnp or gross domestic or gross national)).ti,ab. (265) |
| 70 (low adj3 middle adj3 countr*).ti,ab. (19230) |
| 71 (lmic or lmics or third world or lami countr*).ti,ab. (8631) |
| 72 transitional countr*.ti,ab. (162) |
| 73 Developing Countries/ (75877) |
| 74 (Albania or Algeria or American Samoa or Angola or Armenia or Azerbaijan or Bangladesh or Belarus or Belize or Benin or Bhutan or Bolivia or "Bosnia and Herzegovina" or Botswana or Brazil or Bulgaria or Burkina Faso or Burundi or Cabo Verde or Cambodia or Cameroon or Central African Republic or Chad or China or Colombia or Comoros or Democratic Republic of Congo or Republic of Congo or Costa Rica or Cote d'Ivoire or Cuba or Djibouti or Dominica or Dominican Republic or Ecuador or Egypt or El Salvador or Equatorial Guinea or Eritrea or Ethiopia or Fiji or Gabon or Gambia or Georgia or Ghana or Grenada or Guatemala or Guinea or "Guinea-Bissau" or Guyana or Haiti or Honduras or India or Indonesia or Iran or Iraq or Jamaica or Jordan or Kazakhstan or Kenya or Kiribati or North Korea or Kosovo or Kyrgyz Republic or Lao PDR or Lebanon or Lesotho or Liberia or Libya or Macedonia or Madagascar or Malawi or Malaysia or Maldives or Mali or Marshall Islands or Mauritania or Mauritius or Mexico or Micronesia or Moldova or Mongolia or Montenegro or Morocco or Mozambique or Myanmar or Namibia or Nauru or Nepal or Nicaragua or Niger or Nigeria or Pakistan or Papua New Guinea or Paraguay or Peru or Philippines or Romania or Russian Federation or Rwanda or Samoa or "Sao Tome and Principe" or Senegal or Serbia or Sierra Leone or Solomon Islands or Somalia or South Africa or South Sudan or Sri Lanka or "St. Lucia" or "St. Vincent and the Grenadines" or Sudan or Suriname or Swaziland or Syrian Arab Republic or Tajikistan or Tanzania or Thailand or Timor-Leste or Togo or Tonga or Tunisia or Turkey or Turkmenistan or Tuvalu or Uganda or Ukraine or Uzbekistan or Vanuatu or Venezuela or Vietnam or "West Bank and Gaza" or Yemen or Zambia or Zimbabwe).hw,kf,ti,ab,cp. (2769041) |
| 75 (Africa or Caribbean or West Indies or South America or Latin America or Central America or Middle East or South Asia or Southeast Asia).hw,kf,ti,ab,cp. (244313) |
| 76 67 or 68 or 69 or 70 or 71 or 72 or 73 or 74 or 75 (2927762) |
| 77 7 and 31 and 66 and 76 (4294) |
| 78 limit 77 to yr="1990 - 2020" (4229) |

**Table C in S1 Appendix . List of low- and middle-income countries**

| **Low – and middle-income countries** |
| --- |
| - Albania - Algeria - American Samoa - Angola - Armenia - Azerbaijan - Bangladesh - Belarus - Belize - Benin - Bhutan - Bolivia - Bosnia and Herzegovina - Botswana - Brazil - Bulgaria - Burkina Faso - Burundi - Cabo Verde - Cambodia - Cameroon - Central African Republic - Chad - China - Colombia - Comoros - Democratic Republic of Congo - Republic of Congo or Costa Rica - Cote d'Ivoire - Cuba - Djibouti - Dominica - Dominican Republic - Ecuador - Egypt - El Salvador - Equatorial Guinea - Eritrea - Ethiopia - Fiji - Gabon - Gambia - Georgia - Ghana - Grenada - Guatemala - Guinea - Guinea-Bissau - Guyana - Haiti - Honduras - India - Indonesia - Iran - Iraq - Jamaica - Jordan - Kazakhstan - Kenya - Kiribati - North Korea - Kosovo - Kyrgyz Republic - Lao PDR - Lebanon - Lesotho - Liberia - Libya - Macedonia - Madagascar - Malawi - Malaysia - Maldives - Mali - Marshall Islands - Mauritania - Mauritius - Mexico - Micronesia - Moldova - Mongolia - Montenegro - Morocco - Mozambique - Myanmar - Namibia - Nauru - Nepal - Nicaragua - Niger - Nigeria - Pakistan - Papua New Guinea - Paraguay - Peru - Philippines - Romania - Russian Federation - Rwanda - Samoa - Sao Tome and Principe - Senegal - Serbia - Sierra Leone - Solomon Islands - Somalia - South Africa - South Sudan - Sri Lanka - St. Lucia - St. Vincent and the Grenadines - Sudan - Suriname - Swaziland - Syrian Arab Republic - Tajikistan - Tanzania - Thailand - Timor-Leste - Togo - Tonga - Tunisia - Turkey - Turkmenistan - Tuvalu - Uganda - Ukraine - Uzbekistan - Vanuatu - Venezuela - Vietnam - West Bank and Gaza - Yemen - Zambia - Zimbabwe |

We used the 2019 World Bank income group to classify countries into low- and middle-income.

Note: Include studies if they were conducted in both LMICs and high-income countries.

**Table D in S1 Appendix . Data Extraction Tool**

|  | **Variables to be extracted** | **Notes** |
| --- | --- | --- |
|  | Study author |  |
|  | Study title |  |
|  | Year of publication |  |
|  | Country of corresponding author |  |
|  | Country of implementation |  |
|  | Study funding sources reported | Yes or No |
|  | Name of funding source if reported |  |
|  | Type of funding source, if applicable | 1. Local 2. International 3. Other |
|  | Primary aim of the study | If standalone implementation |
|  | Secondary aim of the study | If implementation embedded in effectiveness |
|  | Standalone vs embedded | 1. Standalone implementation study 2. Implementation embedded in effectiveness study |
|  |  |  |
|  | Type of Methods/Approach | 1. Quantitative 2. Qualitative 3. Mixed method |
|  | Major study designs | 1) Experimental design: randomized controlled trial or cluster randomized control trial or randomized stepped wedge  2) Quasi-experimental trial (participants NOT randomized) *synonym observational trial:  a) Single Interrupted time series  b) Controlled (multiple interrupted time series  c) Pre-post study with comparison group  d) Regression discontinuity  e) Non-randomized stepped wedge  3) Pre-experimental trial (no control group or no repeat measures: pre-post study)  4) Other observational (including non-intervention) studies  a) Cohort study (prospective or retrospective)  b) Cross-sectional study (outcomes and predictors assessed at same time point, i.e., from survey)  c) Case control study  d) Qualitative research (main aim is qualitative)  e) Economic evaluation  5) Multiple study types reported  6) Other (indicate which ones) |
|  | Study designs | 1. Randomized controlled trial 2. Cluster randomized control trial 3. Randomized stepped wedge 4. Single Interrupted time series 5. Controlled (multiple interrupted time series 6. Pre-post study with comparison group 7. Regression discontinuity 8. Non-randomized stepped wedge 9. Pre-post study (no-control) 10. Cohort study (prospective or retrospective) 11. Cross-sectional study 12. Case control study 13. Qualitative research 14. Economic evaluation 15. Other (indicate which ones) |
|  | Type of mixed method study (if applicable) | 1. Sequential exploratory 2. Sequential explanatory 3. Concurrent 4. Other |
|  | Type of hybrid study (if applicable) | 1. Type 1 2. Type 2 3. Type 3 |
|  | Level of health system targeted† | 1. Micro-level 2. Meso-level 3. Macro-level |
|  | Implementation science framework guiding the implementation research | Whether used (YES / No). If yes, which one. |
|  | Name the implementation framework used | 1. RE-AIM framework 2. CFIR 3. Diffusion of innovation 4. PEPFAR implementation science framework 5. CDC REP framework 6. EPIS framework 7. ExpandNet Scaling Up framework 8. Promoting Action on Research Implementation in Health Services (PARIHS) framework 9. Other (please specify) |
|  | Type of NCDs/risk factors targeted | 1. Tobacco use 2. Harmful use of alcohol 3. Unhealth diet 4. Physical inactivity 5. Cardiovascular disease 6. Diabetes 7. Cancer 8. Chronic respiratory disease 9. Other |
|  | Intervention type | See Appendix Table 1 |
|  | Implementation outcomes categories | 1. Acceptability 2. Adoption 3. Appropriateness 4. Feasibility 5. Fidelity 6. Penetration 7. Sustainability 8. Implementation costs 9. Research 10. Maintenance 11. Other |
|  | Implementation strategies | 1. Actor 2. Action target 3. Recipients |
|  | Study population | 1. General population; 2. High risky population; 3. Disease profile (healthy population); 4. Age/sex |
|  | Sample size | Sample frame size, N included, N with complete follow un information |
|  | Pilot or scale up project | 1. Pilot/ demonstration project 2. scaled up project. 3. Other |
|  | Level of scale up | 1. Sub-national 2. National 3. Regional 4. Other |
|  | Study considered equity | 1. Yes 2. No |
|  | Equity lens | 1. Disaggregated data by key SES stratifiers. 2. Vulnerable population? 3. Was the sample size adequate for this? |

† *Micro-level* refers to the point where the care providers interact with the patient; micro-level interventions aim to directly influence the performance of the staff or the operations of a facility [2, 3]. *Meso-level* refers to the level responsible for service areas/clinical programs providing care for a similar group of patients, typically part of a larger organization (e.g., subnational intervention targeting improvement of a network of facilities and communities) [2, 3]. *Macro-level* is the highest (strategic) level of the system, an umbrella including all intersecting areas, departments, providers and staff (eg, boards, healthcare network, integrated health system that includes several organizations); macro-level interventions are best able to directly tackle the social, political, economic, and organisational structures that shape a health system [2, 3].

**Table E in S1 Appendix . Distribution of studies by countries where they were implemented**

| Country of implementation* | Income group* | Number of studies | Percentage |
| --- | --- | --- | --- |
| India | Lower middle income | 37 | 14.7 |
| China | Upper middle income | 34 | 13.5 |
| Brazil | Upper middle income | 15 | 6 |
| South Africa | Upper middle income | 11 | 4.4 |
| Iran | Lower middle income | 8 | 3.2 |
| Kenya | Lower middle income | 8 | 3.2 |
| Multiple | - | 8 | 3.2 |
| Thailand | Upper middle income | 8 | 3.2 |
| Peru | Upper middle income | 7 | 2.8 |
| Uganda | Low income | 7 | 2.8 |
| Malaysia | Upper middle income | 6 | 2.4 |
| Nigeria | Lower middle income | 6 | 2.4 |
| Pakistan | Lower middle income | 6 | 2.4 |
| Vietnam | Lower middle income | 6 | 2.4 |
| Guatemala | Upper middle income | 5 | 2 |
| Tanzania | Lower middle income | 5 | 2 |
| Argentina | Upper middle income | 4 | 1.6 |
| Cameroon | Lower middle income | 4 | 1.6 |
| El Salvador | Lower middle income | 3 | 1.2 |
| Ghana | Lower middle income | 3 | 1.2 |
| Indonesia | Lower middle income | 3 | 1.2 |
| Malawi | Low income | 3 | 1.2 |
| Mexico | Upper middle income | 3 | 1.2 |
| Bangladesh | Lower middle income | 2 | 0.8 |
| Bolivia | Lower middle income | 2 | 0.8 |
| Botswana | Upper middle income | 2 | 0.8 |
| Costa Rica | Upper middle income | 2 | 0.8 |
| Ethiopia | Low income | 2 | 0.8 |
| Fiji | Upper middle income | 2 | 0.8 |
| Guyana | Upper middle income | 2 | 0.8 |
| Jordan | Upper middle income | 2 | 0.8 |
| Lao PDR | Lower middle income | 2 | 0.8 |
| Myanmar | Lower middle income | 2 | 0.8 |
| Sri Lanka | Lower middle income | 2 | 0.8 |
| Zambia | Lower middle income | 2 | 0.8 |
| Bhutan | Lower middle income | 1 | 0.4 |
| Bosnia and Herzegovina | Upper middle income | 1 | 0.4 |
| Bulgaria | Upper middle income | 1 | 0.4 |
| Burkina Faso | Low income | 1 | 0.4 |
| Chile | High income | 1 | 0.4 |
| Colombia | Upper middle income | 1 | 0.4 |
| Cuba | Upper middle income | 1 | 0.4 |
| Dominican Republic | Upper middle income | 1 | 0.4 |
| Ecuador | Upper middle income | 1 | 0.4 |
| Georgia | Lower middle income | 1 | 0.4 |
| Grenada | Upper middle income | 1 | 0.4 |
| Honduras | Lower middle income | 1 | 0.4 |
| Madagascar | Low income | 1 | 0.4 |
| Mali | Low income | 1 | 0.4 |
| Moldova | Upper middle income | 1 | 0.4 |
| Mongolia | Lower middle income | 1 | 0.4 |
| Mozambique | Low income | 1 | 0.4 |
| Nicaragua | Lower middle income | 1 | 0.4 |
| Paraguay | Upper middle income | 1 | 0.4 |
| Philippines | Lower middle income | 1 | 0.4 |
| Romania | Upper middle income | 1 | 0.4 |
| Samoa | Lower middle income | 1 | 0.4 |
| Senegal | Lower middle income | 1 | 0.4 |
| Serbia | Upper middle income | 1 | 0.4 |
| Tunisia | Lower middle income | 1 | 0.4 |
| Turkey | Upper middle income | 1 | 0.4 |
| Uruguay | High income | 1 | 0.4 |
| Zimbabwe | Lower middle income | 1 | 0.4 |

* Countries from high income countries (e.g., Chile) were included in this appendix table and in the map if they were part of multi-country studies that included LMICs.

Fig A in S1 Appendix . Variation of conditions evaluated by income group


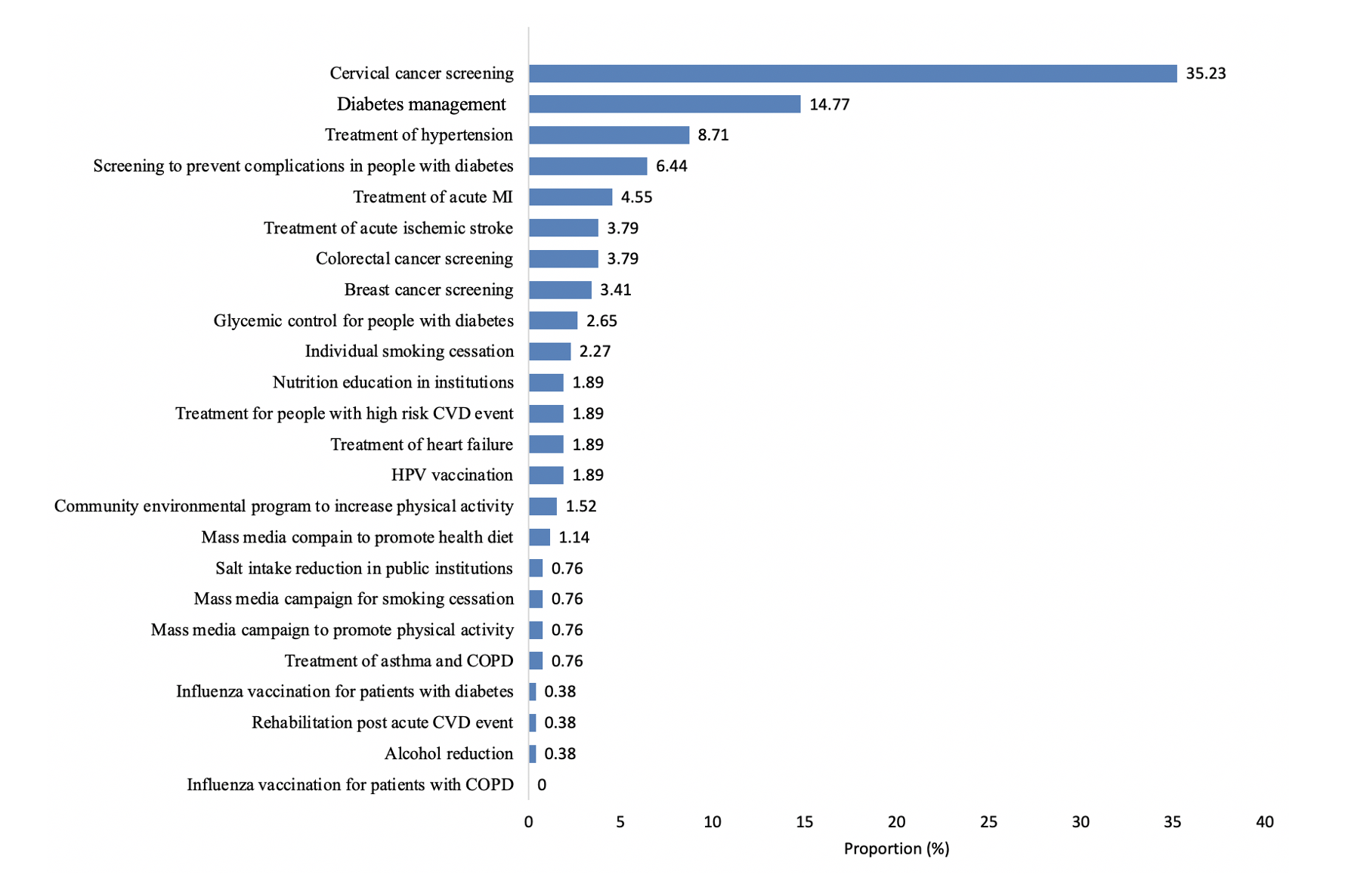


Fig B in S1 Appendix . Priority NCD interventions (n=265) identified in 222 studies included in the review.


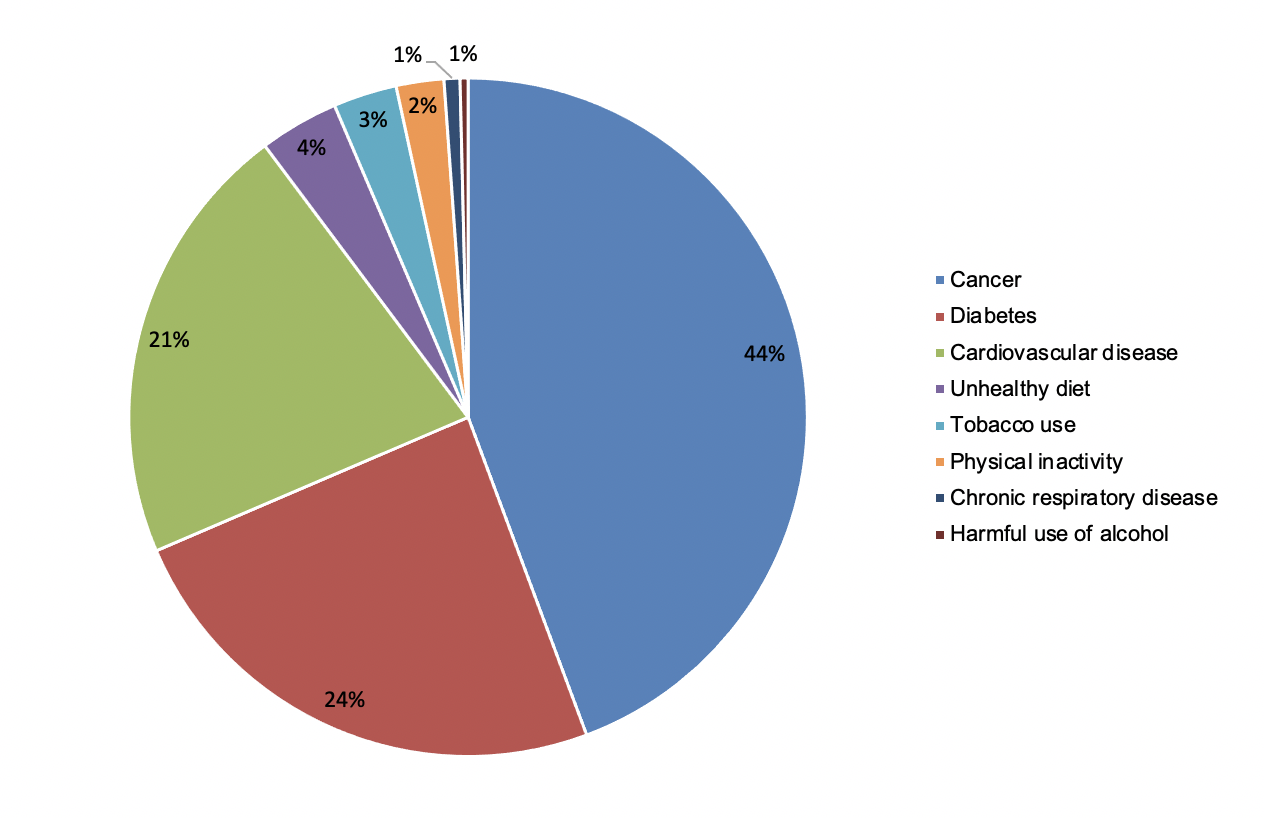


Fig C in S1 Appendix . Distribution of included studies by NCD conditions

Fig D in S1 Appendix . Distribution of intervention type by income group

Fig E in S1 Appendix . Distributions by research designs

Fig F in S1 Appendix . Distributions by standalone implementation studies vs embedded or hybrid effectiveness-implementation studies

Fig G in S1 Appendix . Distributions by pilot vs scale up project

Fig H in S1 Appendix . Variation by level of health system

*Micro-level* refers to the point where the care providers interact with the patient; micro-level interventions aim to directly influence the performance of the staff or the operations of a facility [2, 3]. *Meso-level* refers to the level responsible for service areas/clinical programs providing care for a similar group of patients, typically part of a larger organization (e.g., subnational intervention targeting improvement of a network of facilities and communities) [2, 3]. *Macro-level* is the highest (strategic) level of the system, an umbrella including all intersecting areas, departments, providers and staff (eg, boards, healthcare network, integrated health system that includes several organizations); macro-level interventions are best able to directly tackle the social, political, economic, and organisational structures that shape a health system [2, 3].


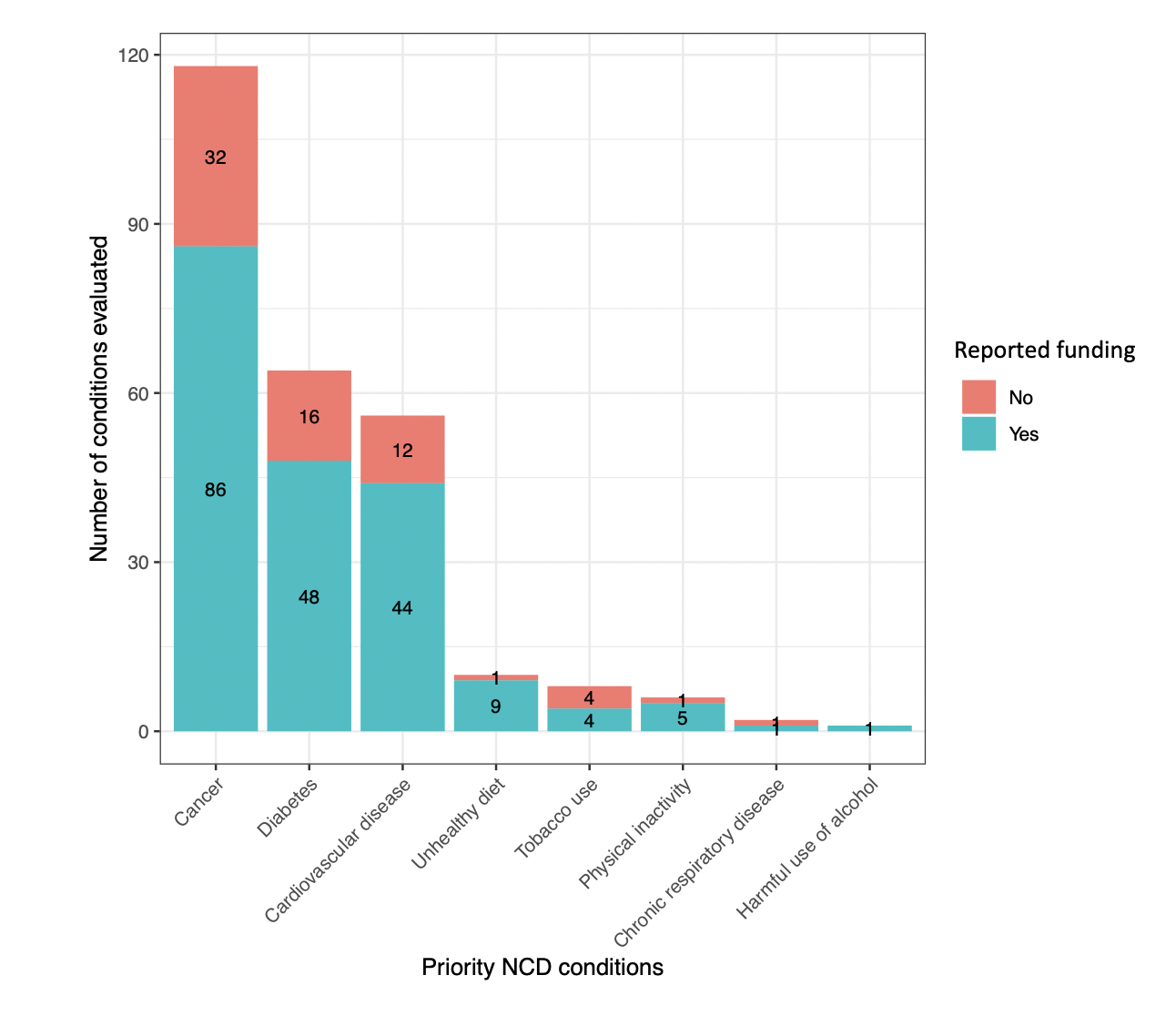


Fig I in S1 Appendix . Studies that reported funding (vs those that did not) by NCD conditions

Fig J in S1 Appendix . Distributions by funding type


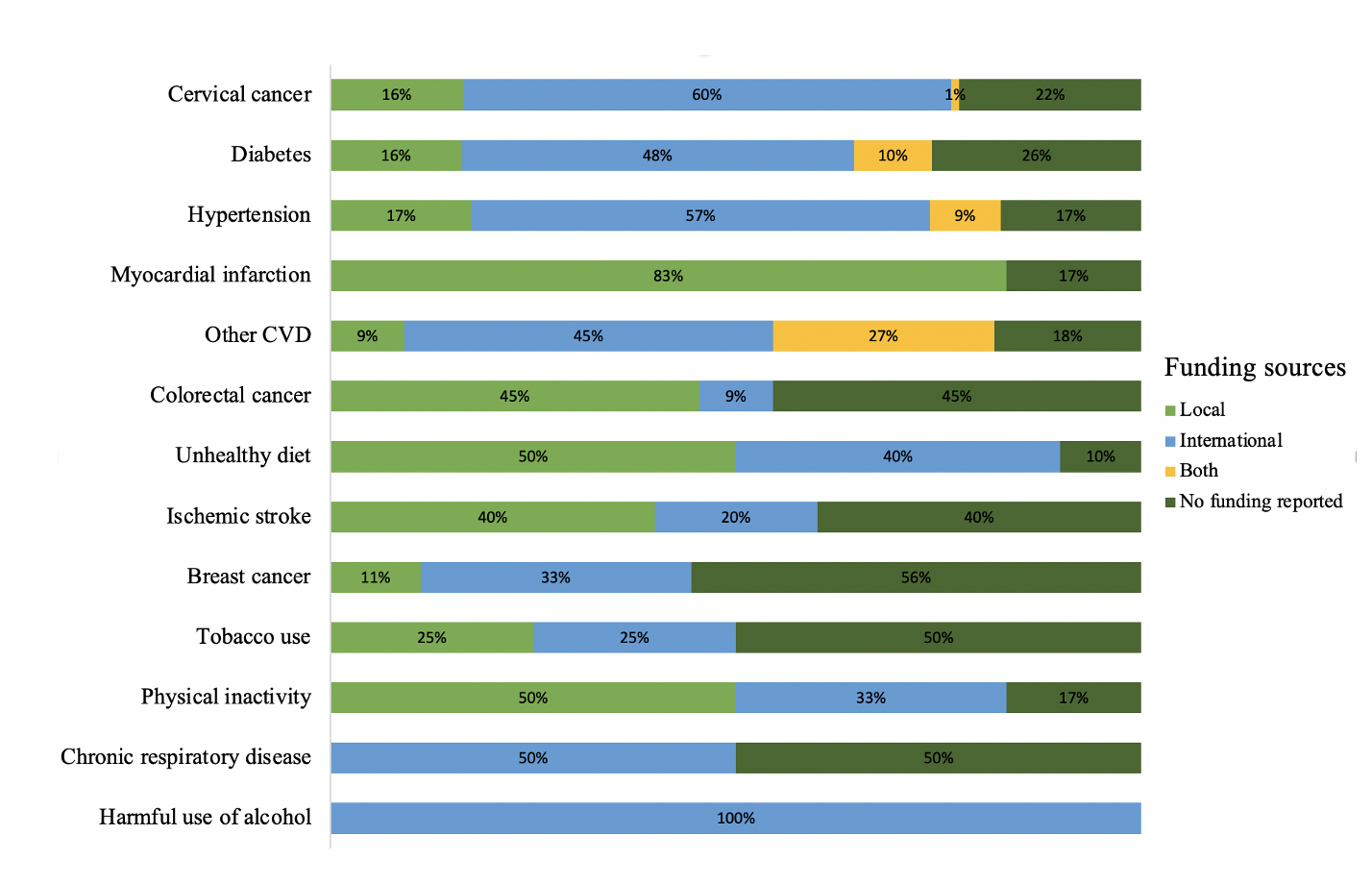


Fig K in S1 Appendix . Distribution of funding sources by NCDs and their risk factors.


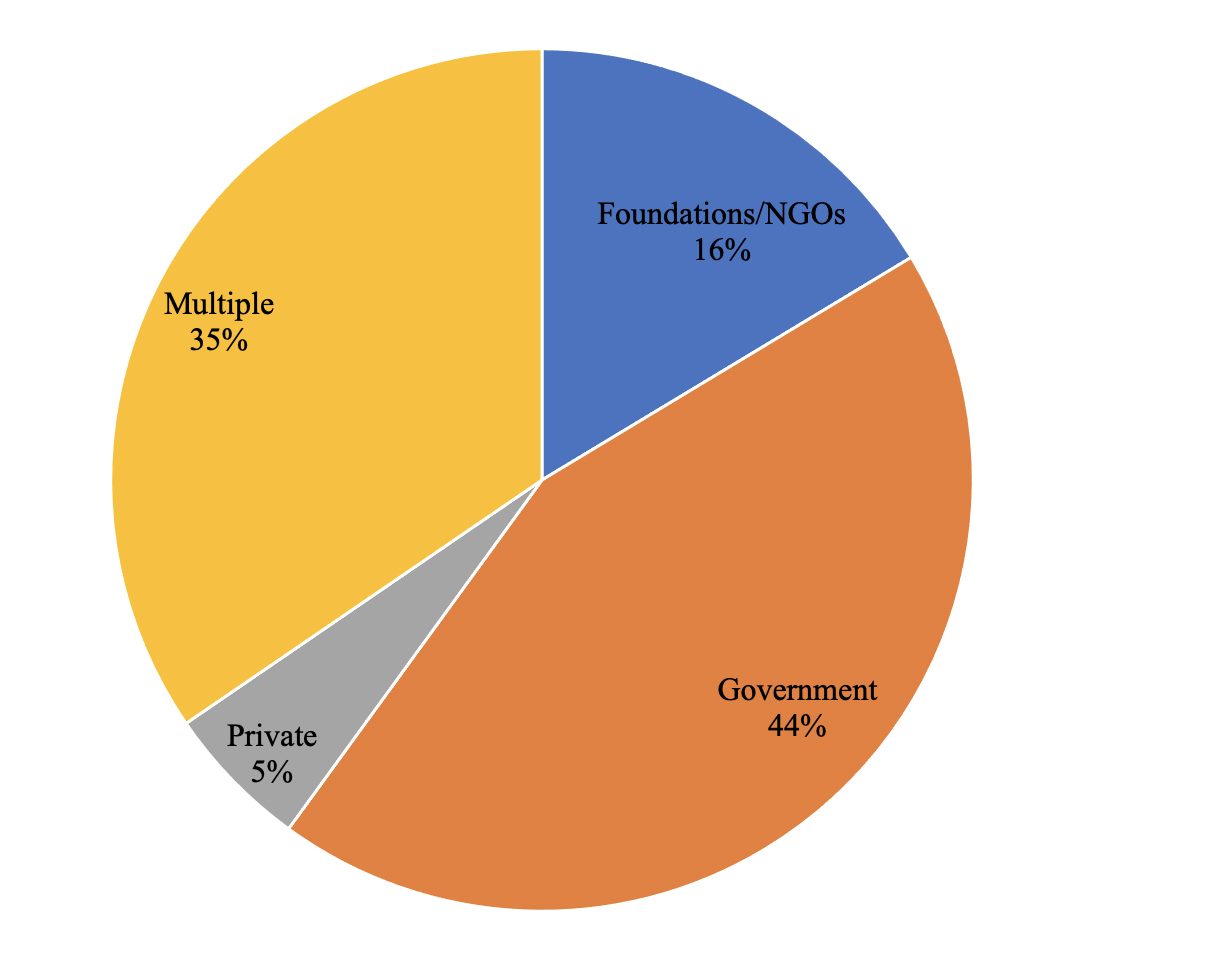


Fig L in S1 Appendix . Types of reported funding sources (N=222 included studies)

**References**

1. World Health Organization. Updated appendix 3 of the who global NCD action plan 2013-2020. Technical Annex. 2017.

2. Beard P, Greenall J, Hoffman C, Nettleton S, Popescu I, Ste-Marie M. Incident Analysis Collaborating Parties. Canadian Incident Analysis Framework. Edmonton, AB: Canadian Patient Safety Institute; 2012 [cited 2013 Dec 14].

3. Kruk ME, Gage AD, Arsenault C, Jordan K, Leslie HH, Roder-DeWan S, et al. High-quality health systems in the Sustainable Development Goals era: time for a revolution. The Lancet global health. 2018;6(11):e1196-e252.
