# Supplementary material for: Implementation research on noncommunicable disease prevention and control interventions in low- and middle-income countries: A systematic review
Source: PLoS Med. 2022 Jul 25;19(7):e1004055. doi: 10.1371/journal.pmed.1004055 (PMC9359585; doi:10.1371/journal.pmed.1004055)
Supplement: Attachment — Submitted filename: hategeka.pdf [file pmed.1004055.s004.pdf]

This paper describes a systematic review of implementation research on non–communicable disease in low– and middle–income countries.

I was asked for a statistical report and I interpret that to include all aspects of the design and conduct of the study.

Points of detail

Page 5 Including studies in any language is obviously a good thing but it is to some extent vitiated by the choice of bibliographic databases. Various empirical studies have indicated that the main bibliographic databases have varied and incomplete coverage of material not in English (Pilkington et al., 2005; Shenderovich et al., 2016), and have limited coverage of material from low and middle–income country journals (Kieling et al., 2009; Syed Sheriff et al., 2008). It would be worth looking at African Index Medicus or LILACS.

Page 5 How were the articles in languages other than English dealt with? Was the single data extractor fluent in all the languages or were they professionally translated?

Page 5 It is usual to specify the date up to which the databases were searched (See the updated PRISMA recommendations Page et al., 2021, Table 1, item 6)

Page 6 It is not optimal to use one reviewer without information about their training and the study which established their validity. Some of the items in Appendix Table 4 do seem to allow scope for individual judgment.

Page 6 I am not clear about the distinction which the authors wish to draw between 'risk of bias' and 'stronger/weaker designs'. My understanding of weaker designs is that they are the ones at highest risk of bias and contrariwise.

Page 7 The detail given in Figure 1 is rather confusing and does not seem to fit with the description given in the methods. For instance on page 5 we learn there was no restriction for language of publication but in the figure there were 4 omitted due to language. What in Figure 1 does 'Full text' mean? Was the full text not available? What does 'Time' mean? Did the study cover an inappropriate time period or did the current authors run out of time?

Page 7 Do the interventions vary by condition and type? The referenced

figures just show proportions which obviously are not equal but that seems hardly worth saying. Have I missed the force of the authors' remarks here?

Page 7 Appendix Figure 4 does not, unfortunately, contain absolute numbers (a defect which occurs throughout) so we cannot perform the simple statistical confirmation of the statement that the 'focus appear to vary by countries'. The same is true of trying to confirm from Appendix Figure 7 whether 'Study designs also appear to vary by NCD conditions targeted.' Various other later statements have the same omission.

Figure 2 Choosing two shades of blue here does not make this easy to read. I do not have a colour vision anomaly but other readers might. Absolute numbers would be better here and if they were placed above the bars they would be legible.

Figure 4 This uses lots of ink to give us a small number of proportions. A table would be more economical and could give us the absolute numbers too.

Figure 5 I am afraid I do not understand this at all. If the number of studies continues to rise how do we explain the dramatic drop in interventions in 2020?

Figure 6 Some of this is hard to read. I cannot see the five regions in quasi–experimental that I was expecting. What is the 1 floating between 45 and 9 which does not seem to have its own colour? At least this one has absolute frequencies.

The authors may feel that I am obsessed with minor details of figures but the point of them is to convey information to the reader and if they are hard to interpret they lose their value.

Points of more substance

What is an NCD?

This may seem obvious to the authors but I do not see why cervical cancer is here classified as an NCD. I believe it is currently thought to be a consequence predominantly of an infection (with human pappilomavirus).

The authors have included alcohol use disorder as one of their NCDs but what about substance use disorder? There is also of course the whole range of mental health conditions which are not covered here. (Disclaimer: I have

worked for many years in mental health and have been involved in studies of health service delivery interventions in LaMICs.)

The authors include stroke but there are other neurological conditions like epilepsy and the dementing illnesses which are not communicable.

If there is an internationally recognised definition of NCD, perhaps from WHO, then that needs to be made clear.

Synthesis methods

I appreciate the authors have found such a range of studies that providing the usual quantitative synthesis is unwise but as it stands we know what studies have been carried out very little of what happened when they were. For instance how many of these came to a successful conclusion and how many had to be abandoned? Given the difficulties of this sort of research especially in LaMICs that would be valuable information.

The authors might also be interested in some of the insights in the guidelines for synthesis without meta–analysis (SWiM) (Campbell et al., 2020).

Summary

Quite a few presentational issues and some concerns about the coverage.

Michael Dewey

References

M Campbell, J E McKenzie, A Sowden, S V Katikireddi, S E Brennan, S Ellis, J Hartmann-Boyce, R Ryan, S Shepperd, J Thomas, V Welch, and H Thomson. Synthesis without meta–analysis (SWiM) in systematic reviews: reporting guideline. *British Medical Journal*, 368:l6890, 2020.

C Kieling, H Herrman, V Patel, and J J Mari. Indexation of psychiatric journals from low– and middle–income countries: a survey and a case study. *World Psychiatry*, 8:40–44, 2009.

M J Page, J E McKenzie, P M Bossuyt, I Boutron, T C Hoffmann, C D Mulrow, L Shamseer, J M Tetzlaff, E A Akl, S E Brennan, R Chou, J Glanville, J M Grimshaw, A Hróbjartsson, M M Lalu, T Li, E W Loder, E Mayo-Wilson, S McDonald, L A McGuiness, L A Stewart, J Thomas, A C Tricco, V A Welch, P Whiting, and D Moher. The PRISMA 2020 statement: an

updated guideline for reporting systematic reviews. *British Medical Journal*, 372:n71, 2021.

K Pilkington, A Boshnakova, M Clarke, and J Richardson. 'No language restrictions' in database searches: what does this really mean? *The Journal of Alternative and Complementary Medicine*, 11:205–207, 2005.

Y Shenderovich, M Eisner, C Mikton, F Gardner, J Liu, and J Murray. Methods for conducting systematic reviews of risk factors in low– and middle–income countries. *BMC Medical Research Methodology*, 16(32), 2016.

R J Syed Sheriff, C E Adams, P Tharyan, M Jayaram, L Duley, and the PRACTIHC Mental Health Group. Randomised trials relevant to mental health conducted in low and middle–income countries: a survey. *BMC Psychiatry*, 8(69), 2008.
