# Supplementary material for: Implementation research on noncommunicable disease prevention and control interventions in low- and middle-income countries: A systematic review
Source: PLoS Med. 2022 Jul 25;19(7):e1004055. doi: 10.1371/journal.pmed.1004055 (PMC9359585; doi:10.1371/journal.pmed.1004055)
Supplement: Attachment — Submitted filename: Response to the PLOS Medicine Staff.docx [file pmed.1004055.s003.docx]

Response to the PLOS Medicine Staff’s comments

1. **The manuscript file is over our file size limit of 10MB. This limit is in place for the convenience of reviewers, editors and readers. Please adapt this file so that the file size is below 10MB.**

We have moved the supporting information file from the manuscript file, reducing its size to less than 10 MB. The manuscript file is now 9.5 MB. The supporting information file has been zipped (size 2.8 MB) and uploaded separately.
