# Supplementary material for: Implementation research on noncommunicable disease prevention and control interventions in low- and middle-income countries: A systematic review
Source: PLoS Med. 2022 Jul 25;19(7):e1004055. doi: 10.1371/journal.pmed.1004055 (PMC9359585; doi:10.1371/journal.pmed.1004055)
Supplement: Attachment — Submitted filename: Hategeka et al. Responses to EditorsReviewers FEB25.docx [file pmed.1004055.s005.docx]

We appreciate the thorough review of the manuscript by the editors and reviewers. Below, we discuss the changes we made as a result.

**Responses to the editors:**

**1)      Please add this statement to the manuscript's Competing Interests: "Margaret Kruk is an Academic Editor on PLOS Medicine's editorial board."**

We have updated as suggested.

**2)      Abstract:
a)      Please report your abstract according to PRISMA for abstracts, following the PLOS Medicine abstract structure (Background, Methods and Findings, Conclusions)** [**http://www.plosmedicine.org/article/info:doi/10.1371/journal.pmed.1001419**](http://www.plosmedicine.org/article/info:doi/10.1371/journal.pmed.1001419) **.**

We have updated the abstract to conform to the PRISMA checklist

**b)      Please combine the “Methods” and “Findings” sections into one “Methods and findings” section**

We have combined the “Methods and findings” as suggested.

**c)      Please ensure that all numbers presented in the abstract are present and identical to numbers presented in the main manuscript text.**

We have double-checked the numbers in the abstract to make sure they are consistent with what we reported in main manuscript text.

**d)      Please summarize the synthesis/appraisal methods**

We have reported how the extracted data were synthesized. As for appraisal methods, given the focus of this review and the heterogeneity in aims and methodologies of included studies, risk of bias assessment to understand how effect size may have been compromised by bias is not applicable. We instead commented on the distribution of research designs and discussed about stronger/weaker designs.

**3)      Please remove the “Research in Context” section**

We have removed it as suggested

**4)      At this stage, we ask that you include a short, non-technical Author Summary of your research to make findings accessible to a wide audience that includes both scientists and non-scientists. The Author Summary should immediately follow the Abstract in your revised manuscript. This text is subject to editorial change and should be distinct from the scientific abstract. Please see our author guidelines for more information:** [**https://journals.plos.org/plosmedicine/s/revising-your-manuscript#loc-author-summary**](https://journals.plos.org/plosmedicine/s/revising-your-manuscript#loc-author-summary)

We have included Author Summary as suggested

**5)      Please submit a completed PRISMA checklist. We understand that not all items on the checklist will be directly relevant, and these can be marked as ‘not applicable’. 
a)      The PRISMA guidelines provided at the EQUATOR site** [**http://www.equator-network.org/reporting-guidelines/prisma/**](http://www.equator-network.org/reporting-guidelines/prisma/) 
**b)      When completing the checklist, please use section and paragraph numbers, rather than page numbers.**

We have submitted a completed PRISMA checklist as suggested.

**6)      Please rename figure 1 to “PRISMA flow chart”**

We have renamed it as suggested

**7)      In line with PLOS Medicine’s guidelines, please update your search to the present time and provide the beginning and end dates of your search.**

We appreciate your suggestion to update our search to the present time; however, the review findings are still quite current and novel, and updating the search is a substantial undertaking and would unfortunately significantly lengthen the time until we would have a version ready for submission. Additionally, we do not currently have the necessary resources to carry out all necessary steps including screening, full text review and reanalysis, if we were to update the search to the present time. We do, however, plan to update the review every five years—systematic reviews are generally a moving target—to track changes over time in implementation research on non-communicable disease prevention and control interventions in low- and middle-income countries. We have provided the beginning and end dates of our search as well as the last time we conducted the search.

**8)      References: 
a)      Please select the PLOS Medicine reference style in your citation manager. In-text reference call outs should be presented as follows noting the absence of spaces within the square brackets, e.g., "... countries [1,2]."
b)      Please ensure that journal name abbreviations consistently match those found in the National Center for Biotechnology Information (NCBI) databases.** [**https://journals.plos.org/plosmedicine/s/submission-guidelines#loc-references**](https://journals.plos.org/plosmedicine/s/submission-guidelines#loc-references)**. 
c)      Please ensure six names appear before et al. e.g., check refs #26-32, 34-36, 38 and so forth**

We have updated references as suggested.

# Responses to Reviewer #1: Points of detail

# Page 5 Including studies in any language is obviously a good thing but it is to some extent vitiated by the choice of bibliographic databases. Various empirical studies have indicated that the main bibliographic databases have varied and incomplete coverage of material not in En- glish (Pilkington et al., 2005; Shenderovich et al., 2016), and have lim- ited coverage of material from low and middle–income country journals (Kieling et al., 2009; Syed Sheriff et al., 2008). It would be worth look- ing at African Index Medicus or LILACS.

We appreciate your comments and your sharing of relevant references, some of which we have now cited in our review. We opted to include MEDLINE and EMBASE as they are the major indexing databases for biomedical research, allowing us to retrieve most studies. This is consistent with systematic reviews published in PLOS MEDICINE. For example, this review “Zhang TN, Huang XM, Zhao XY, Wang W, Wen R, Gao SY. Risks of specific congenital anomalies in offspring of women with diabetes: A systematic review and meta-analysis of population-based studies including over 80 million births. PLoS medicine. 2022 Feb 1;19(2):e1003900” also searched in the two databases.

We agree that despite using rigours search strategies without language restrictions, studies published in journals not indexed in MEDLINE and EMBASE were not captured as acknowledged in our limitations (we have now cited the references you shared - please see page, paragraph). Further, searching additional databases such as African Index Medicus (AIM) may not have necessarily resulted in additional eligible studies. For example, A recent study showed that indexing of active African journals was higher in EMBASE than in African Index Medicus, suggesting that looking at AIM may not result in additional eligible studies (Asubiaro 2021).

Asubiaro, T., 2021. Sub-Saharan Africa's Biomedical Journal Coverage in Scholarly Databases: A comparison of Web of Science, Scopus, EMBASE, PubMed, African Index Medicus and AfricanJournals Online.

**Page 5 How were the articles in languages other than English dealt with? Was the single data extractor fluent in all the languages or were they professionally translated?**

Our team members included researchers who are fluent in other languages including Chinese and French and were assigned studies in these languages.

**Page 5 It is usual to specify the date up to which the databases were searched (See the updated PRISMA recommendations Page et al., 2021, Table 1, item 6).**

We have specified the date in the search strategy sub-section of the Methods Section.

**Page 6 It is not optimal to use one reviewer without information about their training and the study which established their validity. Some of the items in Appendix Table 4 do seem to allow scope for individual judgment.**

Given the number of the studies we included, we allocated individual reviewers to a portion of the studies to extract data. Ahead of data extraction, we conducted several calls to standardize the approach for data extraction. Early in the extraction, we had meetings to compare data extracted to ensure consistency. Any concerns raised during data extraction were discussed and disagreement were resolved by the senior author. All team members had public health training, with minimum of master’s and all reviewers had past experience conducting systematic reviews.

**Page 6 I am not clear about the distinction which the authors wish to draw between ‘risk of bias’ and ‘stronger/weaker designs’. My understanding of weaker designs is that they are the ones at highest risk of bias and contrariwise.**

Although biases tend to be common in weaker designs, both weaker and stronger designs can be affected by bias at the design stage, analytical stage, or result reporting stage. Risk of bias assessment focuses on evaluating potential biases within each study designs including weaker/stronger designs. In this review, we aimed to describe type of study designs and implementation outcomes used in included studies rather than synthesizing intervention effects. As such, a formal risk of bias assessment is not applicable. However, we commented on the distribution of research designs and discussed about stronger/weaker designs. For example, we reported that a quarter of included studies employed were weakest study design, pre-experimental; whereas about another quarter used the strongest design, experimental.

**Page 7 The detail given in Figure 1 is rather confusing and does not seem to fit with the description given in the methods. For instance on page 5 we learn there was no restriction for language of publication but in the figure there were 4 omitted due to language. What in Figure 1 does ‘Full text’ mean? Was the full text not available? What does ‘Time’ mean? Did the study cover an inappropriate time period or did the current authors run out of time?**

While our search in databases was not restricted to any language, during study screening / review processes we only retained eligible studies that were in six official languages of the United Nations (ie, Arabic, Chinese, English, French, Russian and Spanish). We have now added a note in the Methods Section to clarify (please see page 6, paragraph 1).

Full text means that studies were excluded because full text was not available; whereas time refers to studies that were excluded because they were published before/conducted before 1990. We have added a note under Figure 1 to clarify.

**Page 7 Do the interventions vary by condition and type? The referenced**

**figures just show proportions which obviously are not equal but that seems hardly worth saying. Have I missed the force of the authors’ remarks here?**

We have updated the figure number and also included absolute numbers. Figure 2 showed variation in distribution of priority NCD prevention and control interventions by type of NCD and their risk factors.

**Page 7 Appendix Figure 4 does not, unfortunately, contain absolute num- bers (a defect which occurs throughout) so we cannot perform the sim- ple statistical confirmation of the statement that the ‘focus appear to vary by countries’. The same is true of trying to confirm from Appendix Figure 7 whether ‘Study designs also appear to vary by NCD conditions targeted.’ Various other later statements have the same omission.**

We have updated the figure with absolute numbers and have also changed our statements saying the variation is by regions (not by countries). However, even without these numbers the Appendix Figure 4 clearly shows that the type of intervention varies by regions. For example, only screening interventions were reported in studies conducted in low-income countries.

**Figure 2 Choosing two shades of blue here does not make this easy to read. I do not have a colour vision anomaly but other readers might. Absolute numbers would be better here and if they were placed above the bars they would be legible.**

We have changed the color as suggested and have also added absolute numbers of interventions evaluated (please see the updated Figure 2).

**Figure 4 This uses lots of ink to give us a small number of proportions. A table would be more economical and could give us the absolute numbers too.**

We agree with the broader point; however, we think these visual presentations are easier for readers to see information than a single table. We have, however, increased the size to optimize readability. We would be happy to reformat these figures or replace them with a table if the editorial team think it is absolutely necessary. We have added number (N=222) in the title of the figures.

**Figure 5 I am afraid I do not understand this at all. If the number of studies continues to rise how do we explain the dramatic drop in interventions in 2020?**

Figure 5 A shows number of studies published each year (total=222); whereas Figure 5B shows distributions of NCD interventions evaluated (total=265) by type of interventions (primary, screening and treatment). The statement of increasing number of studies over time refer to Figure 5A. We have now revised the title of the figure 5 to avoid the confusion.

**Figure 6 Some of this is hard to read. I cannot see the five regions in quasi– experimental that I was expecting. What is the 1 floating between 45 and 9 which does not seem to have its own colour? At least this one has absolute frequencies.**

We have reformatted the figure to make it easy to read.

**The authors may feel that I am obsessed with minor details of figures but the point of them is to convey information to the reader and if they are hard to interpret they lose their value.**

We appreciate your comments and have addressed them as appropriate (please see our responses above). We also reformatted all Figures in supplementary file to include absolute numbers/frequencies.

# Points of more substance

**What is an NCD? This may seem obvious to the authors but I do not see why cervical cancer is here classified as an NCD. I believe it is currently thought to be a consequence predominantly of an infection (with human pappilomavirus). The authors have included alcohol use disorder as one of their NCDs but what about substance use disorder? There is also of course the whole range of mental health conditions which are not covered here. (Disclaimer: I have worked for many years in mental health and have been involved in studies of health service delivery interventions in LaMICs.) The authors include stroke but there are other neurological conditions like epilepsy and the dementing illnesses which are not communicable. If there is an internationally recognised definition of NCD, perhaps from WHO, then that needs to be made clear.**

NCDs, including cardiovascular diseases, cancer, diabetes, chronic respiratory diseases and mental disorders, are not passed from person to person and tend to be of long duration and are the result of a combination of genetic, physiological, environmental and behaviours factors. The choice of interventions included in this review was based on the updated appendix 3 of the WHO Global NCD Action Plan 2013-2020. These interventions included in the appendix target four types of NCD—cardiovascular diseases, cancer, chronic respiratory diseases and diabetes—which make the largest contribution to morbidity and mortality due to NCDs, and four shared behavioural risk factors—tobacco use, unhealthy diet, physical inactivity and harmful use of alcohol. Substance use disorder, mental health conditions, epilepsy, and dementia are NOT included in this appendix. As such, they were not eligible for inclusion in this review. Like the reviewer, we believe they should be featured in WHO list of priority NCD interventions and articulated this in the limitations. Presumably, cervical cancer has been included since all cancers are classified as NCDs.

- World Health Organization. Updated appendix 3 of the WHO Global NCD Action Plan 2013-2020. Technical Annex. 2017.
- World Health Organization. The WHO Global NCD Action Plan 2013-2020

## Synthesis methods

**I appreciate the authors have found such a range of studies that providing the usual quantitative synthesis is unwise but as it stands we know what studies have been carried out very little of what happened when they were. For instance how many of these came to a successful conclusion and how many had to be abandoned? Given the difficulties of this sort of research especially in LaMICs that would be valuable information. The authors might also be interested in some of the insights in the guidelines for synthesis without meta–analysis (SWiM) (Campbell et al., 2020).**

The reviewer raised an important point; however, the focus on the current review was on synthesizing evidence on what interventions have been implemented, where and when they were implemented, and what were the implementation outcomes. Assessing the study findings would be an excellent topic for another review. Thank you for sharing this reference that provides the guidelines for synthesis without meta-analysis (SWiM). These guidelines, however, are not applicable to our review, which does not focus on synthesizing intervention effects among studies included in this review.

# Summary

**Quite a few presentational issues and some concerns about the coverage.**

Thank you for your thorough review.

**Responses to Reviewer #2**

**Overview**

**The investigators conducted a systematic review of evidence on the current state of implementation research (IR) for NCD prevention and control in low-and middle-income countries (LMICs) using MEDLINE and EMBASE databases from 1990 through 2020. They used standard search terms for this topic that are detailed in their documentation. Among more than 9600 potential studies they found 222 eligible studies from 63 countries.  Most studies were on cervical cancer and were for proof of concept or pilots targeted at a micro-level of the healthcare system. Most studies used quantitative methods and weak study designs were common. Study publications increase dramatically over their study timeframe. Major gaps in IR were noted and that published studies were mostly funded by international sources.** 

**General Comments
This is a very well written study that is very important and timely. IR is currently vastly underused in LMICs, and the authors highlight this challenge and the need for more efforts and resources. While they do show convincingly that the number of IR studies annually has increased over their 30-year study timeframe, efforts in this area fall short of what is needed.** 

Thank you.

**There are several areas where the manuscript could be improved. The results seem to be in both the results section some are also in the discussion section. There are many figures and tables and some of the findings could be moved into the text with a concise description and allowing for deletion of some figures/tables. Better description of the inclusion/exclusion criteria would be helpful. A brief clear description of the working definition of screening, treatment and prevention would be useful. 
Some discussion on why so few studies were multinational would be helpful. Also, a brief description of micro, meso and macro health system interventions would be useful. In the discussion, using high income country comparable statistics might be useful to give the context of what is currently the situation (e.g., use of implementation science framework for studies in HIC vs LMIC, etc.). While not the ideal comparator is could help under the challenges in the LMIC context. In the discussion/conclusion, it would be helpful to present some of the challenges and barriers conducting IR in LMICs. This could be very insightful.  Finally, a better description of why descriptive epidemiological studies were excluded is needed. These may contain barriers and facilitators to implementation and be key in designing IR intervention strategies.**

- We have revised our discussion and removed some results that appeared in the discussion.
- We have provided a working definition of screening, prevention, and treatment (see Appendix Table 1).
- We have also provided a description micro-, meso- and macro-levels of health systems under Table 2 and in the relevant figure in appendix.
- We have provided additional details related to inclusion and exclusion under Figure 1.
- The focus of our review does not allow us to understand why few multinational studies were implemented.
- Kirk et al (2015) systematic review of the use of the consolidated framework for implementation research showed most of studies that used CFIR were conducted in high income countries, suggesting that uptake of IR frameworks may be higher in these settings than LMICs. We have cited this study in our discussion.
- Descriptive epidemiological studies that were not part of implementation of relevant NCD interventions were excluded; however, studies on barriers/facilitators of successful implementation of relevant NCD interventions were included provided there were conducted in the context of interventions implementation.
- In the discussion, we have reflected on potential barriers/challenges in conducting IR in LMICs including lack of funding.

**Specific Comments
Funding source section.
While the statement is made that the funders have no involvement in the study, it does not state who the funders were. Suggest including them.** 

We have removed the subsection as it is not consistent with PLOS MEDICINE formatting. However, we have provided the names of funders in the section of funding. We also acknowledged in the competing interest that some of team members were employed by the funders.

**Page 8 first para
…"country of implementation" is a bit unclear. Suggest making it "county where the IR is being conducted" - it seems that is what you are communicating.** 

We have revised as suggested.

**Figure 1. 
More description of the exclusion categories is needed. For example, intervention, and full text are not entirely clear.** 

We have added a clarification note under the figure

**Figure 2
How were studies that had multiple interventions in this graphic? Were they included in all the categories that had interventions?** 

Yes, they were included in all the categories that had interventions.

**Figure 3. 
This plot is driven by county size. Consider a metric that uses a standardization method such as studies per 100,000 population. This may allow for better comparisons, etc.** 

Thank you for the suggestion. We have now reported number of studies per one million population (please see Figure 3 for details).

**Figure 5. 
Seems that A and B should have the same total numbers but in B 2020 is lower than 2020 in A. Please explain/reconcile.**

Figure 5 A shows number of studies published each year (total=222); whereas Figure 5B shows distributions of NCD interventions evaluated (total=265) by type of interventions (primary, screening and treatment). The statement of increasing number of studies over time refer to Figure 5A. We have now revised the title of the figure 5 to avoid the confusion.

**Figure 7. 
Same issue as for Fig 2. How were studies with multiple interventions handled. Some studies may have a N >1. Please clarify.** 

As explained earlier, they were included in all the categories that had interventions / implementation outcomes reported.

**Table 1. 
See above comment about exclusion of epidemiology descriptive studies. They may have barriers and facilitates for IR intervention development.** 

Descriptive epidemiological studies that were not part of implementation of relevant NCD interventions were excluded; however, those that studied barriers/facilitators of successful implementation of relevant NCD interventions were included provided there were conducted in the context of intervention implementation.

**Appendix Table 3. 
Should have a date on it because LMIC countries change their status over time (e.g., move from LIC to LMIC, etc.)**

We have now indicated that we used the 2019 World Bank income group to classify countries into low- and middle-income.

**Appendix Figure 1. 
Diabetes is misspelled**

Thank you for pointing this out. We have fixed it

**Appendix Figure 5. 
See above comments. Macro, Meso, and Micro need short descriptions.**

We have also provided a description micro-, meso- and macro-levels of health systems under Table 2 and in the relevant figure in appendix.

**Appendix Figure 7. 
The legend is off the page.** 

Thank you. We have fixed it and is now Appendix Figure 5

**Appendix Figure 8. 
Legend is confusing as a standalone. Suggest: Funding, no funding (at least none noted in the published report). However, for the study to happen, it must have been funded at some level - so 'no funding" may not work. More detail would be interesting.** 

We have revised the legend to “Reported funding” as suggested. Please see the updated Appendix Figure 9

**Responses to Reviewer #3**

**Thank you for this interesting and useful review of the literature focusing on implementation research on 33 WHO recommended interventions to prevent and control non-communicable diseases in low- and middle-income countries.**

Thank you

**General comments

Overall, the study is well written and easy to follow. I have a series of more specific suggestions and comments below, but my main concern is that you only present descriptive statistics regarding some key characteristics of the identified studies, rather than the narrative synthesis which you planned to do according to the study protocol in PROSPERO. Such a narrative synthesis could have provided the reader with a richer understanding of what included studies found and other aspects beyond the narrower quantitative approach used. If you can, including a narrative synthesis would likely be very interesting. If this is not feasible, you should at least comment on this departure from the study protocol and throughout the paper more clearly state the narrow quantitative scope of the study.**

We have already clarified in our methods section and in the protocol that narrative descriptive analysis that would be used in this review refers to descriptive statistics including summary statistics of type of interventions, study designs, and implementation outcomes (please see our sub-section on data analysis within the Methods Section,). We did not include summary of findings, including intervention effects, among included studies given this was not the focus on the current review.

**Another key aspect which is missing from the paper is an assessment of the quality and usefulness of the included studies. Indeed, you stress the importance of good quality implementation research in several places in the manuscript, yet it is difficult from this study to say anything about the current quality of implementation research on NCD interventions in LMICs.**

Our review aims included describing type of study designs and implementation outcomes used in included studies rather than synthesizing intervention effects. As such, a formal risk of bias assessment is not applicable. However, we commented on the distribution of research designs and discussed about stronger/weaker designs. For example, we reported that a quarter of included studies employed were weakest study design, pre-experimental; whereas about another quarter used the strongest design, experimental.

**Specific comments

-- Title
Since the study focuses on NCD prevention and control interventions and not only care, you could consider replacing "Care" with "prevention and control interventions" in the manuscript title: "Implementation Research on Non-Communicable Disease Prevention and Control Interventions in Low- and Middle-Income Countries: A Systematic Review"**

We have updated as suggested

**-- Abstract
I would not mention the protocol in the abstract.**

It is recommended to include the protocol and many systematic reviews in PLOS MEDICINE include the protocol.

**"We synthesize extracted data narratively using descriptive statistics" - generally narrative synthesis refers to "the use of words and text to summarise and explain the findings of the synthesis", whereas descriptive statistics is a quantitative approach to describe or summarize the characteristics of a sample or data set. Actually, in this study you only present descriptive statistics, no narrative summary or synthesis. Please revise the abstract accordingly.**

We have already clarified in our methods section and in the protocol that narrative descriptive analysis that would be used in this review refers to descriptive statistics including summary statistics of type of interventions, study designs, and implementation outcomes (please see our sub-section on data analysis within the Methods Section,). We did not include summary of findings, including intervention effects, among included studies given this was not the focus on the current review.

**It would be useful if you could mention something about inclusion and exclusion criteria in methods.**

We have updated the methods in the abstract to include inclusion/exclusion criteria.

**"…approximately similar to their proportion of global population…" while this is a nice idea, it does not make sense to make this sort of comparison since you are missing the high-income countries in your review and they would very likely distort these proportions. Also, your assessment isn't quite correct since LICs are home to about 9% of the global population and about 73% live in MICs.**

We have removed the statement from the abstract.
 **"Slightly more studies used the weakest study design, pre-experimental, than the strongest design, experimental (25% versus 24%)" - in my view, it would be more correct to state that these two categories were (almost exactly) equal as the difference is something like 2 studies out of 222.**

We have updated this statement (please see revised abstract).

**"Despite growth in implementation research on NCDs in LMICs" - this statement is not supported by the findings in the abstract, suggest adding a line supporting this.**

We have added a line of results supporting the statement.

**"Future studies should prioritize implementation at scale". One could argue that studying interventions at the pilot stage should be prioritized in order to assess whether to take an intervention to scale in the first place. Is there a risk that advocating for a priority on studying implementation at scale could lead to less well studied interventions going to scale?**

We are not advocating that pilot studies should be stopped; however, the goal of implementation research is to inform implementation at a large scale and promote sustainability of evidence-based interventions. However, in this study we found that most studies were pilot. This phenomenon has been called pilotitis. Given inference from pilot studies does not necessary hold at scale, implementation researchers should also design studies capable of addressing critical implementation issues including scale-up and sustainability.

Huang, F., Blaschke, S. and Lucas, H., 2017. Beyond pilotitis: taking digital health interventions to the national level in China and Uganda. *Globalization and health*, *13*(1), pp.1-11.

Alonge O, Rodriguez DC, Brandes N, Geng E, Reveiz L, Peters DH. How is implementation research applied to advance health in low-income and middle-income countries?. BMJ Global Health. 2019 Mar 1;4(2):e001257.

**"stronger internal validity, be more conceptually-driven and use mixed- methods to understand mechanisms" - while interesting, these conclusions are not supported by the findings stated in the abstract. Note that it should be possible to read the abstract without reading the full manuscript.**

We have updated the abstract. Specifically, we had already reported that about a quarter of studies used the weakest study designs and most of studies used quantitative methods. We have now added that only less than 5% of studies reported using implementation research framework.

**"To maximize impact of the research under limited resources, adding implementation science outcomes to effectiveness research and regional collaborations are promising." Could you clarify what you mean by this statement, and consider whether this conclusion is supported by the findings of the present study?**

This statement means that there is a room to add implementation outcomes to studies that are testing effectiveness of interventions. Given most studies tend to test effectiveness of interventions, adding implementation outcomes would result in increase in hybrid studies that test both effectiveness and implementation of interventions. This conclusion is based on the review findings (please see results where we highlight that most of studies were standalone). We therefore identified missed opportunities in the conclusion.

**-- Research in context
The text provided under "Added value of this study" does not seem to correspond to this heading. I would suggest beginning the paragraph with something like "This study provides the first comprehensive review of implementation research on NCD interventions…"
The text provided under "Implications of all the available evidence" does not seem to be supported by the findings of the present study. Please review this paragraph.**

We have dropped the research in context as it does not align with PLOS MEDICINE guidelines. We replaced it with Author Summary.

**-- Introduction

In the first paragraph, you only mention NCD mortality. I would encourage you to also mention the significant NCD morbidity and its consequences.**

We have revised as suggested (please see paragraph 1 of the Introduction Section)).

**You mention COVID-19 and the interlinkages between NCD morbidity and COVID-19. You could consider also making a similar link to other infectious diseases such as TB and HIV to drive home the point that NCD prevention and control should not be regarded as a vertical issue separated from other health issues.**

Thank you. We have updated our introduction to reflect your suggestions (please see paragraph 2 of the Introduction Section).

**You write that "…the evidence for the clinical effectiveness of most NCD prevention and treatment interventions is well established…" - would you agree with Isaranuwatchai et al. (BMJ 2020;368:m141) who argue that the clinical effectiveness of some NCD prevention and treatment interventions may indeed be contestable or even wasted in some contexts?**

We are aware of this excellent study and cited it. Similar to this study, we really argue to have more contextualized studies including cost-effectiveness in various regions to inform scale up (please see the last paragraph of the Introduction Section). Clearly, cost-effectiveness of the NCD we included in this review will vary depend on local context, and the point we made "…the evidence for the clinical effectiveness of most NCD prevention and treatment interventions is well established…" is just for clinical effectiveness.

**Please consider explicitly stating the study aim and/or research question of the study.**

We have revised to include the study aim/objective in the introduction in the abstract and the main manuscript.

**Given the limited scope of this study, consider rephrasing "In this systematic review, we synthesize evidence on the current state of implementation research" to better reflect that you present descriptive statistics of key characteristics of peer-reviewed publications on implementation research on 33 specific interventions in LMICs in 1990-2020.**

We have revised to articulate that this review is focused on synthesizing evidence on the current state of implementation research on WHO priority NCD prevention and control interventions provided within health systems in LMICs. Please see the last paragraph of the Introduction Section.

**-- Methods

According to the information registered on the PROSPERO database, the study protocol was technically not pre-specified as the start date (Dec 1, 2020) was seven months before the protocol was registered (July 30, 2021), and the anticipated completion date was only one day later (July 31, 2021).**

We submitted the protocol to PROSPERO in a timely manner; however, the registration was delayed because PROSPERO was prioritizing registration of review relevant to COVID-19. To avoid confusion, we have revised the sentence to read as “This systematic review was conducted according to a study protocol registered in PROSPERO (#CRD42021252969)”

**Could you clarify in the methods how you selected the included interventions and specifically how you define "relevant" interventions and "within health systems"? For example, in Table 2 / Appendix table 1 you list "mass media campaigns that educate the public about the harms of smoking/tobacco use and second hand smoke" as an intervention within the health system, whereas it seems like you have excluded other interventions such as taxation and plain packaging.**

We have clarified that we used the updated Appendix 3 of the WHO Global NCD Action Plan 2013-2020 to identify priority NCD prevention and control interventions. Of these interventions, we selected those that are specifically provided by health systems. This was achieved through discussions and consensus.

Mass media campaigns that educate the public about the harms of smoking/tobacco use and second-hand smoke are generally done and coordinated by health systems; however, taxation and plain packaging are outside of the scope of health systems.

**"Low- and middle-income countries as defined by the World Bank." - please specify which version you use as the classification is modified each year and the list has changed a fair bit since 1990. For example, in 1990 both China and India were classified as low-income countries.**

We have clarified that we used the 2019 World Bank list to define income groups.

**Throughout, I would recommend making the reference to the updated Appendix 3 of the WHO Global NCD Action Plan 2013-2020 more explicit to avoid the perception that you came up with your own list of interventions.**

We have made reference to the document as suggested.

**The list of data elements you extracted, presented in appendix table 4, plays a central role in defining the focus of your study. Please describe how you arrived at the 29 included parameters.** 

The first and last authors created the list driven by the study objective. The list was then discussed with other team members to achieve consensus.

**The following sentence is redundant since the information is provided in table 1: "Non-empirical/primary research including reviews meta-analyses, editorials, commentaries, letters to editors, opinion papers, newspaper and protocols, are not eligible for inclusion."**

We have revised to “Non-empirical/primary studies are not eligible for inclusion” and cited the Table.

**As noted in the abstract: As far as I can see, you do not present any narrative summary or synthesis of findings, only some basic descriptive statistics.**

Please see our earlier response.

**The heading "Risk of bias assessment" could be removed from methods since it was not done, unless it is explicitly required by editors. The corresponding paragraph could be moved to the limitations section.**

This subsection is required for systematic review, providing details of what were done (or not done) and why. We have articulated that the focus on this review is to synthesize evidence on what interventions have been implemented, where and when they were implemented, and what were the implementation outcomes. Given the review does not focus on synthesizing intervention effects of included studies, a formal of bias assessment to understand how effect size may have been compromised by bias is not applicable. However, we commented on the distribution of research designs and discussed about stronger/weaker designs. We have also noted this in our limitations.

**Table 1. This table is unnecessarily unwieldy. Specifically I would recommend removing the row for "population" as your review focuses on interventions and not populations (that non-humans are excluded can be left implicit in my opinion), and significantly shorten the study design section - it is not necessary to list all existing study designs here.**

We included this because it is quite standard for systematic review; however, we would be happy to move it to appendix if the editorial team wishes.

**-- Results

Perhaps I am missing something, but I would normally expect there to be a table with the full list of included studies and their characteristics (either in manuscript or in appendix). Currently, there is just a reference to Table 1 (which should not be referenced here as it belongs to methods), followed by references "20-241". In my view, this is not a sufficient way to list the included studies.**

In this review, we included 222 studies (many studies) and we elected to present them in summary manner to make it easy for readers. If the reviewer and editorial team think it is absolutely necessary, we would be happy to include it as appendix.

**I would recommend using the same number of decimal points throughout (you now mix between 1 and 0 decimal points).**

We have revised accordingly.

**You refer to a grand total of 12 figures in the Appendix, all of which are simple bar or pie charts. In my opinion, this information would be far more easily digested if you could present it as one or two tables instead.**

We think these visual presentations are easier for readers to see information than tables. However, we would be happy to follow the editorial guidance.

**According to table 2 you have only 33 eligible interventions in this study - how could you find 265 interventions? Per the study design, you should only be able to find a maximum of 33 interventions. Could you clarify this? (According to Appendix figure 1, you found studies covering 32 of the 33 eligible interventions, and in total there were 265 instances across the 32 interventions).**

Table 2 shows a list of unique interventions that were eligible for inclusion. In the results, we report total number (instead of unique type of interventions) of NCD interventions evaluated in 222 included studies. We found that 265 interventions were evaluated across 222 included studies, meaning that there were some studies that evaluated more than one intervention.

**Appendix figure 1. The figure says "Fiabetes management", please review.**

Thank you for pointing this out. We have fixed it

**How do figure 2 and appendix figure 1 relate? The numbers seem not to match.**

Figure 2 shows distribution of priority NCD prevention and control interventions by type of NCD and their risk factors; where Appendix Figure 1 (is now Appendix Figure 2 in the revised submission) shows only distribution of priority NCD prevention and control interventions. As such, the proportions do not match. For example, in Figure 2, the top bar represents the proportion of all interventions (including screening, prevention and treatment) that focused on cervical cancer; whereas, in Appendix Figure 1 (is now Appendix Figure 2 in the revised submission), the top bar shows just the proportion of interventions that focused on cervical cancer screening.

**Appendix table 5 should have a column with country income category. The column entitled "proportion" should read "percentage".**

We have fixed it.

**Sorry to make an editorial comment, but please make sure the order of figures and tables are correct (for example, Appendix figure 2 appears in the text before Appendix figure 1).**

Thank you. We have updated the order

**You state that "The NCD conditions targeted varied by countries" and refer to Appendix Figure 3, but the figure only shows how the number of interventions targeting each condition (though you write number of conditions evaluated) varied by country income category. Please revise the text and figure.**

Thank you. We have updated as suggested.

**Could you clarify what you mean by "The NCD interventions varied by conditions and type" - in what way did interventions vary? Perhaps you mean that the number of identified interventions varied?**

Yes, thank you. We did mean that the number of identified interventions differed by conditions and type of intervention.

**When you state "The intervention focus appears to vary by countries" you again seem to be referring to the country income category rather than country.**

Thank you. Yes, we are referring to variation by income category. We have revised accordingly.

**The three types of intervention focus brought up in results (screening, treatment and prevention) do not appear to be presented neither in the methods nor in the appendix - where Appendix table 4, row 22. "intervention type" refers back to Appendix table 1, where in column "category of interventions" you only have primary, secondary or tertiary prevention. Please make sure that all methods used are presented in the methods and that the same terminology is used throughout.**

We have added a note to clarify what was counted as prevention, screening and treatment.

**Please include a definition and reference for what you mean by "level of health system" (micro, meso, macro) in the methods as this is not clear to the reader.**

We have included that as suggested (please see a note under Appendix Table 4, cited in the Methods Section).

**Each time you write that study characteristics varied by income category or condition etc. I wonder whether you did any simple statistical test to assess whether there were significant differences, or did you decide against this?**

We drew attention to substantial discrepancies in number of identified studies/interventions as can be easily seen when looking at relevant figures, but we did not do statistical tests.

**I would suggest mentioning the equity lens in the methods since you bring it up in the results (even though it is presented in Appendix table 4).**

We have added a note on equity lens in the methods (please see the subsection of data analysis and extraction).

**Figure 5A presents the number of publications by year, but it generally takes years from research to publication - do you have any sense of when research was carried out, not only when it was published?**

We reported year of publication and not time of when study/implementation was conducted.

**-- Discussion**

**I am not sure I agree with this statement: "These studies therefore represent the state of the science today on how to scale up the response of the health system to the growing burden of NCDs in the countries bearing the bulk of disease burden from non-communicable conditions." - nowhere in your study design do you mention a focus on "how to scale up the response of the health system", or is this implicit somehow?**

Thank you. We have revised the statement to read as “These studies therefore represent the state of the implementation science in prevention and control of NCDs by health systems to in the countries bearing the bulk of disease burden from non-communicable conditions”.

**Please rephrase as discussed above: "The studies described 265 different NCD interventions, ranging from screening to prevention to treatment and palliation."**

Please see our response above.

**The comparison between number of studies and disease prevalence is interesting but I would recommend adding it to results (and methods) rather than introducing this analysis in the discussion.**

We have moved this analysis in the results section.

**Some of the statements in the discussion appear to be new information, i.e. "We found that only 31 of the studied interventions addressed prevention with nearly 80% of these tackling primary prevention (e.g., tobacco use, inactivity, unhealthy diet)." - could you please make sure that you do not introduce new information in the discussion?**

We have updated the results to include this information.

**You write: "arguably WHO- proposed interventions are well established and a range of implementation models abound" - please see my earlier comment about the fact that the list of interventions has been questioned in its own right. I also note that you contradict yourself when you write that "a range of implementation models abound", while in the introduction you noted that "care delivery models and means of scaling these up to entire populations in need in heterogeneous and resource-constrained health systems are not".**

We have revised to read as arguably WHO-proposed interventions are well established and evidence on (clinical) effectiveness abound as. We are talking about clinical effectiveness including therapeutic models that are available.

**"the micro-level of the health system— targeting patient, provider, or clinic levels", this is the first time in tha manuscript that you explain what you mean by the micro level, see comment above.**

We have added a note explaining these terminologies (please see our earlier response)

**"Nearly one in five tested a new technology, despite evidence that technology adoption without substantial integration into policies, data, and workflows is typically ineffective in transforming care" - are you here implying that this was not the case in the included studies?**

No, as noted earlier, we did not look at the effectiveness of NCD interventions in studies included in this review.

**"The lack of domestic support is unlikely to be offset by global funding going forward; a recent analysis showed that NCDs were under prioritized in bilateral agency portfolios relative to their health impacts." - I would argue that this situation is changing, besides, it may be more useful to think about this in terms of health systems strengthening than as a vertical NCD silo. You may want to touch on that aspect in the discussion.**

We are not arguing for standalone intervention focused on NCDs, but rather to strengthen the health systems to provide optimal care for NCDs.

**You write "We had no language restrictions permitting a comprehensive assessment of the published literature" - did you do any searches in other languages? Did you include studies in other languages?**

While our search in databases was not restricted to any language, during study screening / review processes we only retained eligible studies that were in six official languages of the United Nations (ie, Arabic, Chinese, English, French, Russian and Spanish).

**I would suggest adding a few sentences to the limitations about the fact that you did not assess quality or usefulness of the included studies. (Indeed, you begin the conclusion by stating that "High quality implementation science can play a key role in informing effective delivery of health system interventions", yet your study only quantifies the number of studies, not their quality.)**

We have revised the limitations to reflect your suggestions

**Limitations should also, importantly, discuss the fact that there may be significant implementation research conducted which is never published which may introduce important selection bias in your study.**

While we used robust search strategies in key recommended databases, some studies may have been missed. We have acknowledged that in our limitations.

**The paragraph starting with "Over 50 of the 222 included studies used an experimental research design." seems misplaced after the "strength and limitations" section.**

We have revised the Discussion Section and moved this paragraph before the limitations.

**-- Conclusion
You should generally avoid adding new arguments and references in the conclusion, but rather conclude what you found, relating back to your study aim. Your current conclusion reads more like a conclusion about the usefulness and need for implementation more broadly, rather than about your study findings. Several of the conclusions are not supported by the study findings and should be brought up in the discussion rather than the conclusion. Please revise the conclusion accordingly.**

We have revised the conclusion as suggested.
