# Supplementary material for: Implementation research on noncommunicable disease prevention and control interventions in low- and middle-income countries: A systematic review
Source: PLoS Med. 2022 Jul 25;19(7):e1004055. doi: 10.1371/journal.pmed.1004055 (PMC9359585; doi:10.1371/journal.pmed.1004055)
Supplement: Attachment — Submitted filename: Responses to editors and reviewers May 02.docx [file pmed.1004055.s006.docx]

**Responses to Academic Editor**

**This is an interesting study, but I think two things are missing and could make it much better.

First, there is a notable lack of attention in systematically capturing categorizing the strategies. While the categorization of strategies is a moving target in implementation science, it is nevertheless a crucial "ingredient" in implementation science and our limited ability to describe the strategy (which is the exposure or treatment if you will in this field) limits what we learn from it. There are many different ways of classifying strategies, that range from the original Proctor Actor-Action etc., to ERIC to Cochrane EPOC to Waltz et al. - none of them are perfect but they can still be useful. At a minimum, who in the health system is carrying out the strategy, what are the actions, and who are the actions meant to target are critical. In truth, some of the strategies are going to be difficult to categorize because the primary sources do not say what they are, but it would nevertheless be of interest say how many did or did not. The authors are undoubtedly familiar
with both the challenges and the importance of describing strategies used in these studies, so whatever direction they take, some discussion and justification of it in the discussion would be helpful.**

Thank you for your suggestions. While we had already included data on implementation outcomes of the strategies included in this review, to address your comments we drew on the Proctor et al framework (you suggested) to extract additional data. We were able to additionally extract data on the actor, action target and recipients of the strategies; details of other implementation strategies were not sufficiently described to permit extraction. We have updated the Methods Section to explain this. We have also updated the Results (including Table 3) and Discussion sections.

**Some additional analysis of the implementation outcomes might be useful, with more description on where in the health system (at the patient, the hcw, the organization, the system) etc., the strategies targeted. How has this changed over time? does it differ by disease condition? what about by donor? Likewise this could be done with the strategies as well, but understanding what kind of targets implementation science addressing NCD's seek would be helpful. What I would be concerned about in general is the phenomenon where clinical research and patient level outcomes in clinical medicine drive outcome selection and measurement, and even though much of the action is above the level of the patient, the outcomes - even those considered implementation outcomes - are reported at the level of the patient, and if so would be of limited informativeness.**

Thank you for this suggestion; we agree and we have already included the level of health systems targeted by the strategies. Specifically, we described the three levels of health systems (micro, meso and macro). We reported data on these levels in our main Table 3 (overview of study characteristics) showing distribution of strategies by levels of health systems. We also added a foot note to explain what these levels of health systems refer to. Additionally, we discussed this in our results and discussion sections. Further, we provided an appendix figure showing distribution of NCD conditions/risk factors studied by levels of health systems (please see Appendix Figure 8. Variation by level of health system).

**Responses to comments from the reviewers:

Reviewer #1: The authors have met most of my points especially about the figures although I still think Figure 4 is not really a good use of ink and space.**

We believe the figure helps the reader make sense of the complex data in the study but would be happy to reformat the figure or replace it with a table if the editorial team think it is advisable.

**I thank the authors for the offprint about comparison of databases but I do not draw the same optimistic message from it as they do. Unfortunately it does not give us the breakdown of the intersections of the coverage so we cannot tell if searching a new database would give us additional references. Just because Embase has, say 10%, and AIM 5% does not imply the 5% are a proper subset of the 10%. They might all be different. I still think the search is rather limited.**

Thank you. We agree that the extent of overlap is unclear but we believe it is quite substantial. However, we discuss the database issues in our limitation section: specifically that studies published in journals not indexed in MEDLINE and EMBASE were not considered in our review.


**Reviewer #3: Thank you for your thorough and thoughtful responses to reviewers' and editors' comments. As this is a re-review, I present my remaining concerns below.

You state that "We have already clarified in our methods section and in the protocol that narrative descriptive analysis that would be used in this review refers to descriptive statistics including summary statistics of type of interventions, study designs, and implementation outcomes". My point is that you cannot do descriptive statistics and call it narrative synthesis as those are two different things. Please revise the manuscript accordingly, possibly including an explanation why you did not do a narrative synthesis as you had planned to do according to the study protocol.**

Thanks for clarifying. We have removed the wording narratively to avoid any confusions.

**You state that "Table 2 shows a list of unique interventions that were eligible for inclusion. In the results, we report total number (instead of unique type of interventions) of NCD interventions evaluated in 222 included studies. We found that 265 interventions were evaluated across 222 included studies, meaning that there were some studies that evaluated more than one intervention.". I do not think you have addressed the problem I raised regarding the terminology used, namely that you use the word "intervention" to mean both the 33 priority NCD interventions included in this study and the 265 studied instances of those interventions being used. This is particularly confusing since you actually found 222 studies covering 32 of the 33 interventions. I suggest you consider an alternative terminology which separates between the 33 priority interventions and the 265 instances of interventions being used.**

Thank you. We appreciate this can cause confusion. To this end, we have called the 33, “intervention categories,” and the 265 “interventions” as the latter are specific activities that fall within the broader categories from the WHO. We clarify this throughout the manuscript.

**Regarding your comment that "We reported year of publication and not time of when study/implementation was conducted." I would suggest you make a comment about this in the limitations.**

We have noted this in our limitation section.
