# Supplementary material for: Implementation research on noncommunicable disease prevention and control interventions in low- and middle-income countries: A systematic review
Source: PLoS Med. 2022 Jul 25;19(7):e1004055. doi: 10.1371/journal.pmed.1004055 (PMC9359585; doi:10.1371/journal.pmed.1004055)
Supplement: Attachment — Submitted filename: Responses to Requests from Editors JUN01.docx [file pmed.1004055.s007.docx]

**Responses to Requests from Editors:**

1) **Figure 3 - At the top of each column, please indicate the meaning of numbers in each cell/ row (e.g., “=5”). If this is the N of studies, please clearly indicate this at the top of each column.**

Thank you. We assume the Editors are referring to Table 3 (not Figure 3). In Table 3, the number refers to N of NCD interventions evaluated. We have added N in each column as suggested. We also add a footnote explaining that N refers to number of NCD interventions.

**2)      References 
a)      Please ensure that journal name abbreviations consistently match those found in the National Center for Biotechnology Information (NCBI) databases.** [**https://journals.plos.org/plosmedicine/s/submission-guidelines#loc-references**](https://journals.plos.org/plosmedicine/s/submission-guidelines#loc-references)**. 
b)      Please ensure that an access date is provided for all references with weblinks.**

We have addressed these as suggested.
