# Supplementary material for: Implementation research on noncommunicable disease prevention and control interventions in low- and middle-income countries: A systematic review
Source: PLoS Med. 2022 Jul 25;19(7):e1004055. doi: 10.1371/journal.pmed.1004055 (PMC9359585; doi:10.1371/journal.pmed.1004055)
Supplement: Attachment — Submitted filename: Responses to Requests from Editors.docx [file pmed.1004055.s008.docx]

**Responses to Requests from Editors:

- In the abstract, methods and discussion sections, please clearly mention that the implementation strategies are not specified well enough. This should be highlighted as one of the limitations.**

Thank you for the suggestion. We have revised the abstract, methods and discussion sections accordingly.
